# Integrative In Silico and Experimental Characterization of Endolysin LysPALS22: Structural Diversity, Ligand Binding Affinity, and Heterologous Expression

**DOI:** 10.3390/ijms26178579

**Published:** 2025-09-03

**Authors:** Nida Nawaz, Shiza Nawaz, Athar Hussain, Maryam Anayat, Sai Wen, Fenghuan Wang

**Affiliations:** 1 Beijing Advanced Innovation Center for Food Nutrition and Human Health, Beijing Technology & Business University (BTBU), Beijing 100048, China; nidanawaz771@yahoo.com (N.N.); shizanawaz59@yahoo.com (S.N.); maryamanayat135@gmail.com (M.A.); 2 School of Light Industry Science and Engineering, Beijing Technology & Business University (BTBU), Beijing 100048, China; 3 Department of Food Science and Technology, School of Food and Agricultural Sciences (SFAS), University of Management and Technology (UMT), Lahore 54000, Pakistan; athar.hussain@umt.edu.pk

**Keywords:** antimicrobials, multi-drug resistant Gram-negative and Gram-positive pathogens, bacteriophage, endolysin LysPALS22, molecular docking

## Abstract

Endolysins, phage-derived enzymes capable of lysing bacterial cell walls, hold significant promise as novel antimicrobials against resistant Gram-positive and Gram-negative pathogens. In this study, we undertook an integrative approach combining extensive in silico analyses and experimental validation to characterize the novel endolysin LysPALS22. Initially, sixteen endolysin sequences were selected based on documented lytic activity and enzymatic diversity, and subjected to multiple sequence alignment and phylogenetic analysis, which revealed highly conserved catalytic and binding domains, particularly localized to the N-terminal region, underscoring their functional importance. Building upon these sequence insights, we generated three-dimensional structural models using Swiss-Model, EBI-EMBL, and AlphaFold Colab, where comparative evaluation via Ramachandran plots and ERRAT scores identified the Swiss-Model prediction as the highest quality structure, featuring over 90% residues in favored conformations and superior atomic interaction profiles. Leveraging this validated model, molecular docking studies were conducted in PyRx with AutoDock Vina, performing blind docking of key peptidoglycan-derived ligands such as N-Acetylmuramic Acid-L-Alanine, which exhibited the strongest binding affinity (−7.3 kcal/mol), with stable hydrogen bonding to catalytic residues ASP46 and TYR61, indicating precise substrate recognition. Visualization of docking poses using Discovery Studio further confirmed critical hydrophobic and polar interactions stabilizing ligand binding. Subsequent molecular dynamics simulations validated the stability of the LysPALS22–NAM-LA complex, showing minimal structural fluctuations, persistent hydrogen bonding, and favorable interaction energies throughout the 100 ns trajectory. Parallel to computational analyses, LysPALS22 was heterologously expressed in *Escherichia coli* (*E. coli*) and *Pichia pastoris* (*P. pastoris*), where SDS-PAGE and bicinchoninic acid assays validated successful protein production; notably, the *P. pastoris*-expressed enzyme displayed an increased molecular weight (~45 kDa) consistent with glycosylation, and achieved higher volumetric yields (1.56 ± 0.31 mg/mL) compared to *E. coli* (1.31 ± 0.16 mg/mL), reflecting advantages of yeast expression for large-scale production. Collectively, these findings provide a robust structural and functional foundation for LysPALS22, highlighting its conserved enzymatic features, specific ligand interactions, and successful recombinant expression, thereby setting the stage for future in vivo antimicrobial efficacy studies and rational engineering efforts aimed at combating multidrug-resistant Gram-negative infections.

## 1. Introduction

Endolysins, also called lysins, are highly specialized hydrolytic enzymes synthesized by bacteriophages, viruses that infect bacterial cells. These enzymes have gained considerable attention as a novel class of antimicrobial agents [1], mainly due to their ability to break down peptidoglycan (PG), the primary structural component of bacterial cell walls, thereby facilitating their potent bactericidal effects [2]. Although traditional phage therapy, which employs whole bacteriophages, has demonstrated promising outcomes in Eastern Europe for addressing infections caused by antibiotic-resistant bacteria, its application is constrained by specific challenges [3]. These include the potential for viral adaptation to non-target bacterial strains, the transfer of virulence genes via transduction, and the emergence of bacterial resistance to the phage itself [4]. Consequently, isolated phage-encoded enzymes, particularly endolysins, are increasingly viewed as safer and more controllable alternatives that can also address the escalating problem of multidrug-resistant Gram-positive and Gram-negative pathogens [5]. Endolysins have already found wide-ranging applications, including use in agriculture to generate transgenic plants that are resistant to specific bacterial pathogens, as well as in the food industry to safeguard against contamination by foodborne bacteria [6]. For instance, transgenic tomato plants expressing the endolysin from the CMP1 phage, which confers resistance to *Clavibacter michiganensis* (the causative agent of anthracnose) [7], and transgenic potatoes expressing T4 lysozyme [8], which are resistant to *Pectobacterium carotovora*, have been successfully developed [9]. Moreover, endolysins’ specificity and rapid bactericidal mode of action make them valuable agents in clinical settings, offering possibilities for reducing biofilms and enhancing or replacing traditional antibiotic therapies [10]. These enzymes can be classified according to the chemical bonds they cleave in the PG structure—amidases, endopeptidases, lysozymes, glycosaminidases, and lytic transglycosylases—and exhibit substantial structural diversity reflective of their distinct host specificities [11].

Furthermore, structural analyses reveal that endolysins derived from phages infecting Gram-positive bacteria often display a modular organization comprising an enzymatically active domain (EAD) and one or more cell wall binding domains (CBDs), connected by flexible linkers [12]. This structural design facilitates precise recognition and hydrolysis of PG, with the EAD governing substrate cleavage and the CBD ensuring specificity by targeting particular epitopes on bacterial cell surfaces [13,14]. By contrast, Gram-negative endolysins tend to be smaller globular proteins that frequently rely on a single catalytic domain, although multidomain variants have also been reported [15]. Additional structural adaptations, such as the inclusion of hydrophobic amino acid residues or the engineering of flexible linkers, can further enhance the bactericidal efficiency of these enzymes against antibiotic-resistant strains [16]. Given the expanding utility of endolysins, modern approaches increasingly integrate in silico methods—ranging from modeling and molecular docking to protein–ligand interaction analysis—to dissect active sites, predict substrate specificity, and evaluate thermodynamic properties under various conditions. These computational approaches help to overcome the challenges of crystallizing endolysins with bacterial PG components, complementing traditional wet-lab experiments and accelerating the discovery of novel or engineered endolysins with improved therapeutic potential [17].

Equally important is the breadth of activity that certain endolysins can display against diverse bacterial taxa, including Gram-negative species from the ESKAPE group (*Enterococcus faecium*, *Staphylococcus aureus*, *Klebsiella pneumoniae*, *Acinetobacter baumannii*, *Pseudomonas aeruginosa* and *Enterobacter* spp.), whose escalating antibiotic resistance poses a significant threat to public health. The adaptability of endolysins to cleave structurally distinct PG types illustrates their potential as next-generation antimicrobials [18]. Because their catalytic mechanism rapidly lyses bacterial cells, endolysins can often act faster than conventional antibiotics, while also posing a lower risk of resistance development [19]. This dual advantage has spurred efforts to design fusion constructs that combine distinct domains, thereby potentially expanding the bacterial host range and reinforcing cell wall binding under varied environmental or physiological conditions. Therefore, bioinformatic mining of phage genomes, structural analyses, and molecular simulations all serve as complementary strategies for identifying and optimizing novel lysins. These cutting-edge methods promise to deliver robust, targeted therapeutics that address current gaps in antimicrobial treatment regimens, especially as the burden of multidrug resistance escalates worldwide [20]. Despite growing interest in endolysins as antimicrobial agents, detailed comparative analyses combining in silico and experimental approaches remain limited. Here, we hypothesize that an integrative study of selected endolysins, focusing on their structural diversity and binding affinities, alongside experimental expression and characterization of LysPALS22, will elucidate key functional features relevant for therapeutic development.

Thus, the present study applies an integrative approach combining in silico analyses and experimental validation to identify, characterize, and compare endolysins. The computational methodology encompasses multiple steps, including sequence assembly and open reading frame selection, phylogenetic assessments, physicochemical property calculations, protein structure modeling, and interaction energy analyses. Comparative molecular docking studies are then performed to explore potential ligand interactions, providing critical insights into substrate specificity and binding affinity. Complementing these computational investigations, the study also includes experimental cloning, heterologous expression, and purification of the LysPALS22 endolysin in *Escherichia coli* (*E. coli*) and *Pichia pastoris* (*P. pastoris*) expression systems. This combined workflow enables validation of the predicted protein characteristics and post-translational modifications, such as glycosylation, while assessing expression yields in different hosts. This integrative computational and experimental strategy is designed to inform the rational design and engineering of potent endolysins for diverse applications, including combating pathogenic bacteria and enhancing existing antimicrobial therapies.

## 2. Result

### 2.1. Identification and Physicochemical Properties

A total of 16 endolysins, targeting various pathogens such as *Acinetobacter baumannii*, *Bacillus cereus*, *Staphylococcus aureus*, *E. coli*, *Pseudomonas aeruginosa*, *Klebsiella pneumoniae*, and *Enterococcus faecalis*, were included in the comparative analysis (Table S1). The longest open reading frame for the Endo_LysPALS22_Ec and Endo_LysPALS22_Pp genes is illustrated in Figures S1 and S2. Moreover, the physicochemical analysis of the endolysins revealed substantial variation in their characteristics. Our cloned proteins, Endo_LysPALS22_Ec (expressed in *E. coli*) and Endo_LysPALS22_Pp (expressed in *P. pastoris*), exhibited distinct features compared to the other proteins listed. Endo_LysPALS22_Ec consists of 322 amino acids with a molecular weight (MW) of 35.784 kDa, a theoretical pI of 9.4, and an instability index of 28.57, indicating moderate stability. It also has the lowest aliphatic index (56.96) and the most negative grand average of hydropathy (GRAVY) score (−0.663) among the proteins. In contrast, Endo_LysPALS22_Pp is larger, containing 412 amino acids, with a MW of 45.251 kDa, a lower theoretical pI of 8.63, and a comparable instability index of 27.9. This variant has a higher aliphatic index (64.68) and a less harmful GRAVY score (−0.476), indicating a moderately hydrophilic character. Other endolysins, such as Endo_LysPA26 and Endo_LysAB-vT2, exhibited higher aliphatic indices (98.07 and 92.25, respectively) and moderately hydrophilic profiles. Endo_LysECD7 and Endo_LysCSA13 were notable for their lower molecular weights (14.742 kDa and 28.363 kDa, respectively), with Endo_LysECD7 showing the lowest instability index (10.63), reflecting exceptional stability.

**Figure 1:**
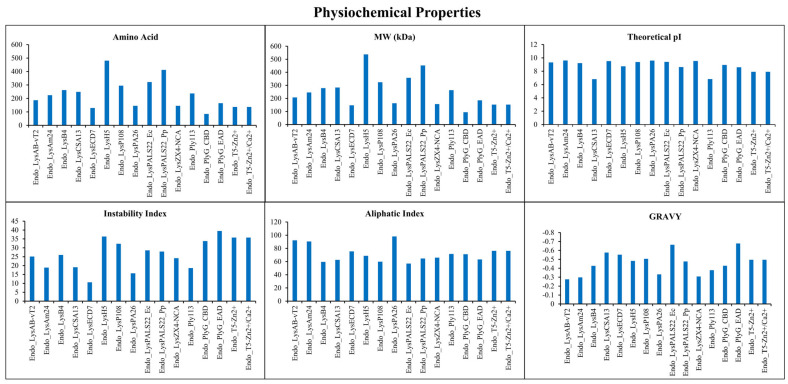
The bar graphs illustrate comparative physicochemical properties. These include amino acid length, molecular weight (MW, kDa), theoretical isoelectric point (pI), instability index, aliphatic index, and GRAVY scores across the sixteen endolysins. Proteins analyzed include the cloned Endo_LysPALS22_Ec (expressed in *E. coli*) and Endo_LysPALS22_Pp (expressed in *P. pastoris*), alongside various other characterized endolysins.

This comparative analysis highlights significant diversity in structural size, stability, hydrophobicity, and solubility profiles among these endolysins, factors critical to their functional expression and potential applications in antimicrobial strategies. These differences are likely shaped by evolutionary pressures and distinct host environmental factors, offering valuable insights into their biochemical behaviors and industrial and therapeutic potential (Figure 1, Table S2).

### 2.2. Multiple Sequence Alignment and Conserved Domains

**Figure 2:**
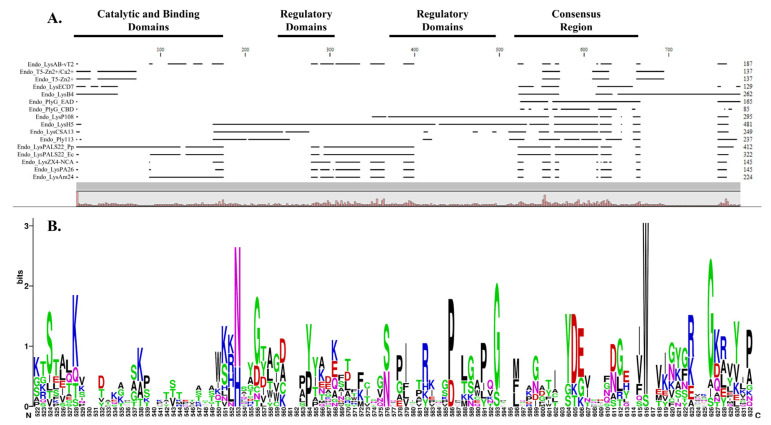
(**A**) Multiple sequence alignment of selected endolysins highlighting conserved catalytic and binding domains, regulatory domains, and a consensus region. (**B**) WebLogo representation of the consensus region (amino acids 522–632) displaying sequence conservation and variability. Larger letters indicate highly conserved residues, emphasizing their importance in maintaining structural integrity and functionality of the endolysins.

The multiple sequence alignment (MSA) of selected endolysins demonstrated significant conservation of specific functional regions, highlighting catalytic, binding, regulatory, and consensus domains across the analyzed proteins. The alignment revealed prominent catalytic and binding domains, consistently observed at the N-terminal region in multiple sequences, crucial for endolysins enzymatic activity and target specificity. Adjacent to this region, two regions showing distinct regulatory domains were identified, suggesting roles in the modulation of enzymatic activity or structural stability. The alignment also delineated a clearly defined consensus region, indicative of highly conserved sequences likely critical for the structural integrity and fundamental enzymatic functions of these proteins. Notably, our cloned proteins, Endo_LysPALS22_Ec and Endo_LysPALS22_Pp, exhibited these conserved domains strategically positioned within commonly observed regions (Figure 2A, Tables S3–S5).

Furthermore, the WebLogo depiction of the consensus region (amino acids 522–632) highlights the degree of sequence conservation and variability among the analyzed endolysins. Prominent residues with high frequency and conservation appear as larger letters in the WebLogo, reflecting their likely importance in maintaining structural integrity and functional roles of these proteins. The presence of conserved polar residues, such as serine (S), lysine (K), and arginine (R), suggests critical involvement in catalytic activity or binding interactions essential to enzymatic function. The consistent conservation of acidic residues such as aspartate (D) and glutamate (E) underscores potential catalytic or structural functions pivotal to protein stability. Additionally, the prevalence of conserved hydrophobic residues, including glycine (G), proline (P), and alanine (A), likely contributes to maintaining the structural framework necessary for functional conformation (Figure 2B). Overall, this consensus region visualization reinforces these residues’ functional significance and evolutionary conservation.

### 2.3. Phylogenetic Tree

**Figure 3:**
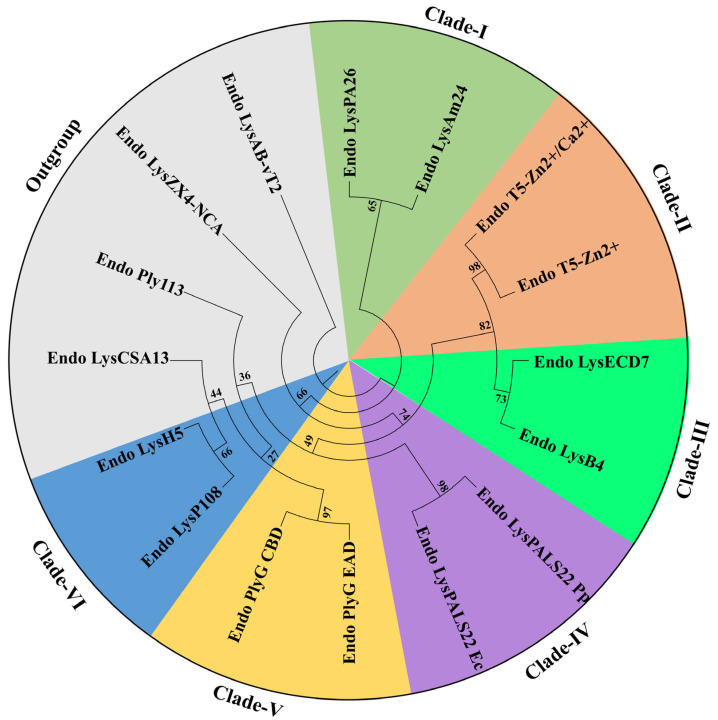
Phylogenetic tree of endolysins, illustrating their evolutionary relationships. The tree is divided into six distinct clades (I–VI), with bootstrap values indicated at branch nodes. The bootstrap values represent the confidence level of each branch, with higher values indicating more substantial support.

The phylogenetic analysis of the selected endolysins revealed their clustering into six distinct clades, underscoring sequence diversity and putative functional relationships among these bacteriophage-derived lytic enzymes. As shown in the circular tree, Clade I, supported by moderate-to-high bootstrap values, includes Endo_LysPA26 and Endo_LysAm24. Clade II comprises Endo_T5-Zn2+ and Endo_T5-Zn2+/Ca2+, demonstrating close evolutionary proximity likely correlated with metal ion coordination sites in their catalytic domains, as suggested by the high similarity in their inferred amino acid sequences. In Clade III, Endo_LysECD7 and Endo_LysB4 form a robust branch (indicated by bootstrap support over 70%), highlighting structural parallels in their peptidoglycan binding modules. Clade IV, which groups Endo_LysPALS22_Ec and Endo_LysPALS22_Pp, exhibits a substantial bootstrap value (90%), suggesting that these proteins, derived from distinct phage isolates but harboring comparable catalytic residues, may have convergently evolved to target a similar range of bacterial hosts. Meanwhile, Clade V (comprising Endo_PlyG_EAD and Endo_PlyG_CBD) indicates a bifunctional architecture where enzymatic and binding regions are likely segregated, reflected by their high pairwise sequence identity in the C-terminal region. Clade VI includes Endo_LysH5 and Endo_LysP108. Finally, Endo_LysAB-vT2, Endo_Ply113, Endo_LysCSA13, and Endo_LysZX4-NCA formed an outgroup with low bootstrap values (36 and 44), indicating high sequence divergence compared to each other and the other endolysins discussed in this study (Figure 3).

### 2.4. Domains and Motifs

**Figure 4:**
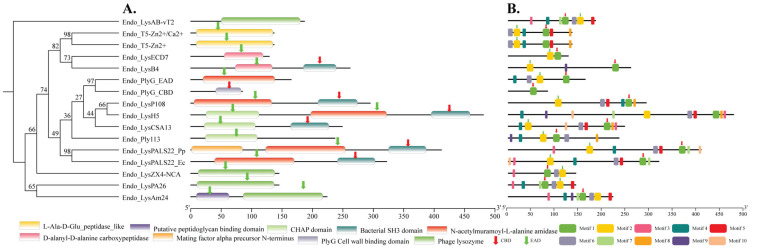
(**A**) Domain architecture (based on InterProScan) and phylogenetic relationships of sixteen selected endolysins. Domains are indicated in the legend with unique coloring. Domains include Phage lysozyme, Putative peptidoglycan binding domain, D-alanyl-D-alanine carboxypeptidase, Bacterial SH3 domain, CHAP domain, N-acetylmuramoyl-L-alanine amidase, mating factor alpha precursor N-terminus, PlyG Cell wall binding domain, L-Ala-D-Glu_peptidase_like. (**B**) shows conserved motifs identified using MEME motif analysis, reflecting functional and structural diversity across the analyzed proteins. The CBD and EAD domains have been highlighted with arrows; red arrow represents CBD, while green arrow indicates EAD with these endolysins.

Domain annotation revealed a modular organization of both enzymatically active domains (EADs) or core catalytic domains and cell wall-binding domains (CBDs) across the analyzed endolysins, with distinct variations in their domain combinations. While single-domain enzymes such as Endo_LysAB-vT2 and Endo_LysPA26 carried only a glycoside hydrolase domain (PF00959; residues 50–181 and 10–138, respectively), others such as Endo_LysAm24 combined an N-terminal peptidoglycan-binding-like domain (PF01471; residues 9–64) with a C-terminal glycoside hydrolase domain (PF00959; residues 86–218). Similarly, Endo_LysB4 and Endo_LysCSA13 displayed modularity with catalytic peptidase or CHAP domains followed by SH3-like CBDs, whereas Endo_LysH5 was notably multi-modular, harboring a CHAP domain (PF05257; residues 25–113), an amidase domain (PF01510; residues 197–323), and a C-terminal SH3-like CBD (PF08460; residues 395–460). In comparison, Endo_LysP108 contained a single amidase domain (PF01510; residues 5–134) and a SH3-like CBD (PF08460; residues 209–275), while Endo_Ply113 and Endo_PlyG_EAD carried CHAP or amidase domains, with Endo_PlyG_CBD restricted to a peptidoglycan-binding domain (PF12123; residues 41–84). Both Endo_T5-Zn2+ and Endo_T5-Zn2+/Ca2+ encoded L-Ala-D-Glu peptidase-like catalytic domains (cd14845; residues 9–136), further emphasizing conservation among phage endolysins. Notably, our target enzyme Endo_LysPALS22_Ec carried a catalytic amidase domain (PF01510; residues 39–171; E-value: 8.40 × 10^−16^) together with a C-terminal SH3-like CBD (PF08460; residues 241–304; E-value: 1.10 × 10^−10^). Its recombinant counterpart, *Endo_LysPALS22_Pp*, retained this core modular architecture with an amidase domain (PF01510; residues 123–255; E-value: 1.40 × 10^−15^) and a SH3-like CBD (PF08460; residues 325–388; E-value: 1.60 × 10^−10^), but in addition contained an N-terminal mating factor alpha precursor domain (PF05436; residues 1–86; E-value: 6.00 × 10^−26^), representing a lineage-specific insertion unique to the cloned construct. Together, these annotations highlight the conserved presence of catalytic domains across all enzymes while underscoring structural variability in CBD composition and additional N-terminal insertions, particularly in Endo_LysPALS22_Pp (Figure 4A, Tables S4 and S6).

Motif analysis further reinforced the modular diversity among the analyzed endolysins, revealing both conserved catalytic signatures and unique accessory elements. Simpler enzymes such as Endo_LysAB-vT2 and Endo_LysPA26 exhibited only a limited number of motifs corresponding to their single catalytic domains, whereas multi-domain endolysins like Endo_LysH5 and Endo_LysCSA13 displayed multiple conserved motifs associated with CHAP, amidase, and SH3-like domains, reflecting their complex organization. Endo_LysAm24 and Endo_LysB4 each carried characteristic peptidoglycan-binding and catalytic motifs, consistent with their dual-domain architecture, while Endo_PlyG was split into CBD- and EAD-specific motifs, highlighting functional partitioning. In contrast, Endo_T5-Zn2+ and Endo_T5-Zn2+/Ca2+ were enriched with motifs associated with L-Ala-D-Glu peptidase-like catalytic activity, indicating conservation of zinc- and calcium-dependent catalytic residues across both constructs. Strikingly, our target enzyme Endo_LysPALS22_Ec contained strongly conserved amidase motifs (residues 84–98; E-value: 2.70 × 10^−7^; sequences CIICVPLDEKAWHVM) and a SH3-like CBD motif (residues 281–295; E-value: 7.60 × 10^−3^; sequences EIQDFNGYIWVSGKF), aligning well with its predicted domain annotation. Its recombinant variant, Endo_LysPALS22_Pp, retained these motifs but also harbored an additional N-terminal signal-associated motif within the mating factor alpha precursor domain, distinguishing it from all other analyzed endolysins. This insertion suggests a potential role in secretion or processing in *P. pastoris*, thereby functionally differentiating Endo_LysPALS22_Pp from the native Endo_LysPALS22_Ec. Collectively, the motif patterns emphasize conservation of core catalytic features across all endolysins while highlighting the unique structural innovation present in Endo_LysPALS22_Ec (Figure 4B, Tables S6 and S7).

### 2.5. Protein Structure Comparison

**Figure 5:**
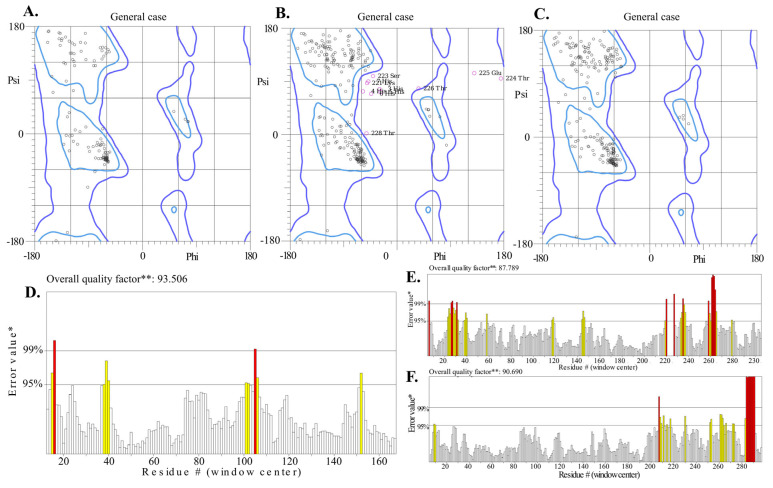
(**A**–**C**) Ramachandran plot of the Endo_LysPALS22_Ec, Endo_PALS22_Ec_AlphaFold, and AF-A0A855GMK5-F1-model_v4 models illustrating the distribution of backbone dihedral angles (φ and ψ). Most residues fall within the core, allowed, and generous regions, respectively, indicating favorable stereochemistry and backbone conformations. (**D**–**F**) ERRAT quality assessment graph of the three endolysins showing the overall quality factor, reflecting the reliability of non-bonded atomic interactions across the protein structure. The red and yellow bars denote regions with the highest error values, suggesting minor local structural deviations. Error value * represents the local quality of individual residues, whereas the Overall quality factor ** indicates the global percentage of residues with acceptable non-bonded atomic interactions (higher values correspond to better overall structural reliability).

The comparative validation of Endo_LysPALS22_Ec structures predicted by Swiss-Model, AlphaFold Colab, and AlphaFold EMBL-EBI revealed clear differences in stereochemical quality and overall model reliability (Table S8). Among the three models, the Swiss-Model prediction consistently demonstrated the most balanced validation profile. It achieved the highest ERRAT score (93.5%) and a favorable VERIFY3D compatibility value (79.8%), while maintaining excellent stereochemical parameters, including 93.2% of residues in favored Ramachandran regions, only 0.62% outliers, and no bad bonds. MolProbity analysis further supported its reliability, with a clashscore of 5.17 (93rd percentile) and a global score of 1.72 (89th percentile), indicating robust stereochemical geometry and minimal steric conflicts. Furthermore, the AlphaFold Colab model was characterized by a high intrinsic confidence (average pLDDT = 87.6) and the strongest VERIFY3D score (87.6%). However, it showed weaker stereochemistry compared to the Swiss-Model, including a higher proportion of Ramachandran outliers (3.44%), elevated clashscore (26.2; 19th percentile), and 3.6% bad bonds, which together suggested less optimal atomic-level accuracy. Contrarily, the EMBL-EBI AlphaFold model achieved the highest stereochemical accuracy by Ramachandran statistics (94.9% favored residues, 0.33% outliers) and the strongest MolProbity validation (clashscore 1.92, 99th percentile; global score 1.30, 98th percentile). However, it performed poorly in VERIFY3D (77.4%), indicating lower agreement between the atomic environment and amino acid sequence. Despite its high average pLDDT score (89.4), this reduced environmental compatibility limited its suitability for downstream docking studies (Figure 5A–F and Figure S3, Table S8).

Taken together, the Swiss-Model structure provided the best compromise across all independent validation metrics, outperforming the AlphaFold models in terms of overall stereochemical quality, non-bonded interaction accuracy, and environmental compatibility. For this reason, the Swiss-Model prediction of Endo_LysPALS22_Ec was selected as the reference model for molecular docking.

**Figure 6:**
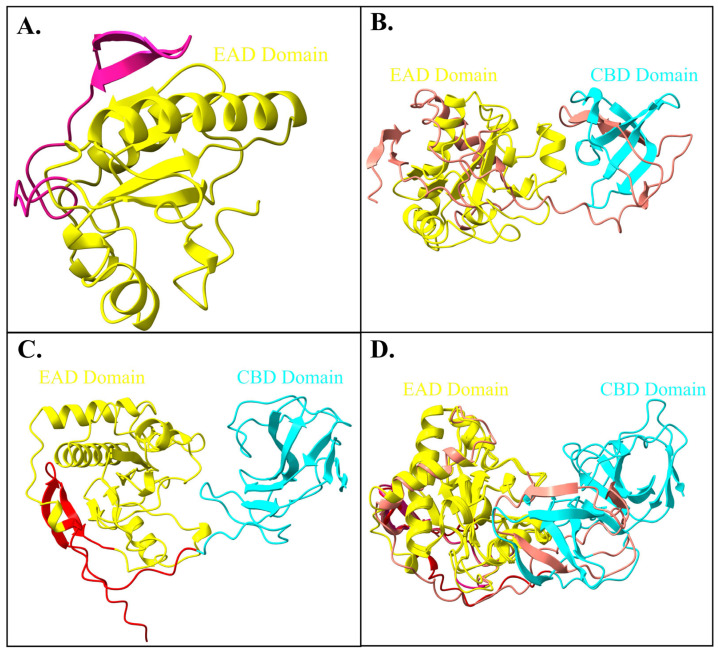
(**A**) represents the 3D structure of Endo_PALS22_Ec with yellow color highlighting the EAD, (**B**) shows the 3D structure of AF-A0A855GMK5-F1-model_v4 (yellow; EAD, cyan; CBD), (**C**) depicts the 3D structure of Endo_PALS22_Ec_AlphaFold, and (**D**) displays a structural comparison of AF-A0A855GMK5-F1-model_v4 and Endo_PALS22_Ec with the reference (Endo_PALS22_Ec_AlphaFold) using the ChimeraX program’s MatchMaker utility.

Subsequently, the structural alignments of Endo_LysPALS22_Ec (obtained from Swiss-Model) (Figure 6A) against two models (obtained from the EMBL-EBI tool; AF-A0A855GMK5-F1-model_v4 (Figure 6B) and AlphaFold colab utility; Endo_PALS22_Ec_AlphFold (Figure 6C) were performed. The first alignment between Endo_LysPALS22_Ec_AlphaFold and AF-A0A855GMK5-F1-model_v4 produced a sequence alignment score of 843.3, with an RMSD of 0.560 Å across 184 pruned atom pairs and 16.059 Å across all 289 pairs, indicating strong local similarity within the aligned core but notable divergence in non-conserved regions. In contrast, the alignment of Endo_LysPALS22_Ec_AlphaFold with the Endo_LysPALS22_Ec structure yielded a slightly higher sequence alignment score of 869.7, with an RMSD of 0.510 Å for 160 pruned atom pairs and 0.615 Å across all 163 pairs, reflecting overall higher structural agreement with minimal global deviations (Figure 6D, Table S9).

**Figure 7:**
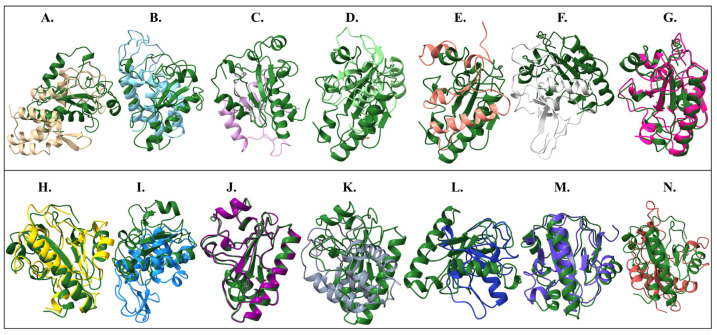
Structural alignment of Endo_PALS22_Ec_AlphaFold_EAD (shown in green) with the EADs of endolysins. The green structure serves as the reference in all alignments. (**A**). Endo_LysAB-vT2_EAD, (**B**). Endo_LysAm24_EAD, (**C**). Endo_LysB4_EAD, (**D**). Endo_LysCSA13_EAD, (**E**). Endo_LysECD7_EAD, (**F**). Endo_LysH5_EAD1, (**G**). Endo_LysH5_EAD2, (**H**). Endo_LysP108_EAD, (**I**). Endo_LysPA26_EAD, (**J**). Endo_LysPALS22_Pp_EAD, (**K**). Endo_LysZX4-NCA_EAD, (**L**). Endo_Ply113_EAD, (**M**). Endo_PlyG_EAD, (**N**). Endo_T5-Zn2+_EAD.

To highlight conserved catalytic cores, structural alignments were performed using the Endo_LysPALS22_Ec_AlphaFold_EAD model as the reference against the EADs of other endolysins. Endo_LysPALS22_Pp_EAD (our recombinant endolysin expressed in *P. pastoris*) exhibited the highest degree of similarity to the reference, with a sequence alignment score of 748.5 and excellent agreement across 138 pruned atom pairs (RMSD = 0.509 Å; all-pairs RMSD = 0.63 Å), confirming that the cloned variant retained a near-identical catalytic fold. High structural conservation was also observed for Endo_PlyG_EAD and Endo_LysP108_EAD, which yielded alignment scores of 227.5 and 149.8, respectively, with RMSDs close to 1.1 Å across 65–97 pruned pairs, suggesting well-preserved core architectures. Similarly, Endo_LysH5_EAD2 aligned closely (score 177.7; RMSD = 0.973 Å over 69 pruned pairs), indicating that one of its EAD modules is structurally consistent with the reference catalytic domain. In contrast, several other endolysins, including Endo_LysAB-vT2_EAD, Endo_LysAm24_EAD, Endo_LysCSA13_EAD, and Endo_LysECD7_EAD, exhibited modest alignment scores (20–35) and only a handful of pruned pairs (4–8), with RMSDs around 1.1–1.5 Å in the aligned regions but substantially higher values when all residues were considered (>11–20 Å). This indicates that only small conserved fragments of their catalytic domains aligned well with the reference, while much of their structure diverged. Other cases, such as Endo_Ply113_EAD, Endo_LysB4_EAD, and Endo_ZX4-NCA_EAD, showed similarly limited overlap (alignment scores <25; 5–7 pruned pairs). Altogether, these domain-focused alignments emphasize the high conservation of the catalytic fold between Endo_LysPALS22_Ec and Endo_LysPALS22_Pp, while revealing varying degrees of structural similarity across other endolysins, with the greatest preservation observed among Endo_PlyG_EAD, Endo_LysP108_EAD, and one catalytic module of Endo_LysH5 (Figure 7A–N, Table 1 and Table S10).

**Figure 8:**
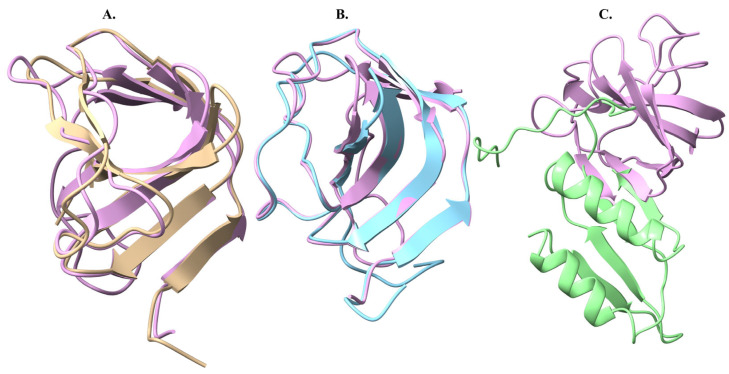
Structural comparison on the basis of CBD. (**A**). Pink (Endo_PALS22_Ec_AlphaFold_CBD) vs. Endo_LysCSA13_CBD, (**B**). Endo_LysH5_CBD, (**C**). Endo_PlyG_CBD_CBD.

Moreover, structural comparisons of the CBDs, using Endo_LysPALS22_Ec_AlphaFold_CBD as the reference, revealed strong conservation with Endo_LysH5_CBD (alignment score 175; RMSD = 0.628 Å across 68 pruned pairs; all-pairs RMSD = 2.443 Å) and Endo_LysCSA13_CBD (alignment score 145; RMSD = 0.717 Å across 63 pruned pairs; all-pairs RMSD = 1.371 Å), indicating that these endolysins share a highly similar cell wall-binding architecture. In contrast, Endo_PlyG_CBD showed only minimal overlap with the reference (alignment score 13.4; 4 pruned pairs), with higher RMSD values (1.342 Å for pruned pairs; 8.431 Å overall), suggesting that its CBD adopts a distinct structural conformation (Figure 8, Table S11).

### 2.6. Solubility, Interaction Energy, and Charge Distribution of Endo_LysPALS22_Ec

The solubility prediction of Endo_PALS22_Ec showed that the protein’s scaled solubility (QuerySol) is 0.442, slightly below the population average of 0.45, indicating marginally reduced solubility. Meanwhile, the deviations from the population average highlighted fluctuations in residues like Lysine (K) and Glutamic acid (E), with positive deviations (green) contributing to higher solubility and negative deviations (yellow), potentially hindering it. The windowed charge score per amino acid revealed positive and negative charges, with a significant portion of negative charges, suggesting regions involved in electrostatic interactions affecting protein stability. Finally, the fold propensity analysis indicated regions with high (orange) and low (blue) folding potential, where low folding propensity areas may contribute to instability and aggregation (Figure S4A–D, Table S12).

Moreover, the energy heatmap analysis showed that the protein’s interaction energy significantly varies with pH and ionic strength. The protein exhibits higher energy at low ionic strength (0.005 M), indicating stronger repulsive interactions, especially at acidic pH levels (2.0–3.0). As ionic strength increases, interaction energy decreases, suggesting more stable interactions, with lower energies observed at higher ionic strengths (0.3 M), particularly around pH 5.0–6.0. The heatmap transitions from red (higher energy) to green (lower energy) to reflect these changes. The bar graphs highlight a consistent trend of decreasing energy with increasing ionic strength, showing distinct profiles for the query protein and Fab set across pH values (Figure S5, Table S13). Notably, the predicted stability window around pH 5–6 and ionic strength 0.3 M coincides with conditions commonly encountered in mildly acidic intracellular compartments and in formulation buffers designed to minimize aggregation. This contextual alignment suggests that the observed solubility and interaction patterns may have practical implications for both physiological stability and formulation strategies.

On the other hand, the charge heatmap analysis revealed that the protein’s charge per amino acid is sensitive to pH and ionic strength. At low ionic strength (0.005 M), the protein retains a negative charge at lower pH values (2.0–3.0), becoming more neutral at higher pH values. With higher ionic strengths (0.3 M), the protein’s charge shifts toward neutrality, particularly at higher pH values. The charge heatmap, color-coded from dark blue (negative charge) to red (positive charge), clearly illustrates how the protein’s charge varies with pH and ionic strength. The charge profile graphs show a consistent trend of decreasing charge per amino acid with increasing pH, with the Fab set consistently exhibiting a more negative charge than the query protein at lower pH (Figure S6, Table S14).

### 2.7. Insights into Protein–Ligand Docking

**Figure 9:**
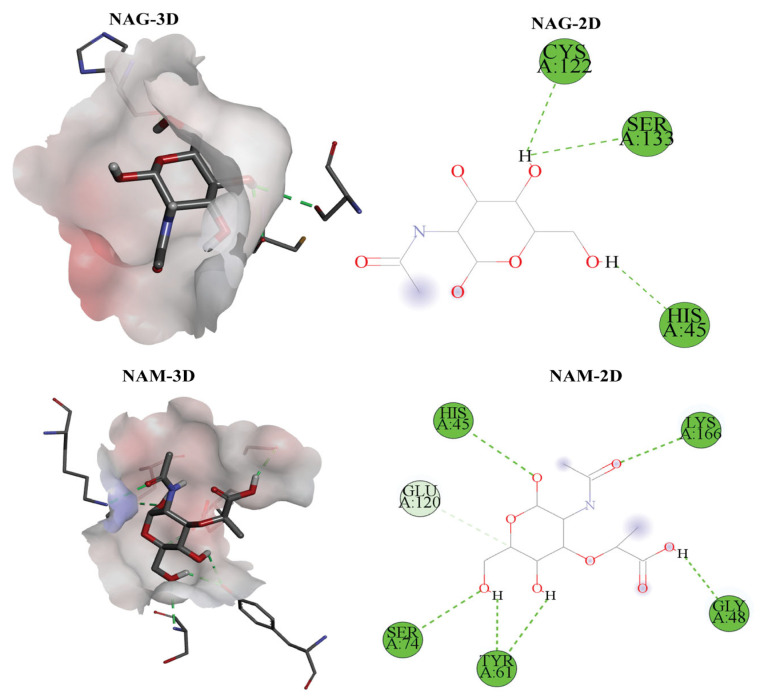
Visual representation of docking interactions between Endo_LysPALS22_Ec and two ligands: N-Acetylglucosamine (NAG) and N-Acetylmuramic Acid (NAM). The 3D panels (**left**) show ligand binding within the active site. At the same time, the 2D interaction diagrams (**right**) illustrate key contacts. Green lines represent conventional hydrogen bonds and light green indicates carbon–hydrogen bonds. Visualization was performed using Discovery Studio Visualizer.

The molecular docking analysis of six biologically relevant ligands against Endo_LysPALS22_Ec revealed significant binding affinities (Vina empirical scores) and interaction profiles. The selection of Endo_LysPALS22_Ec for molecular docking was based on its high sequence conservation, favorable physicochemical properties, and stable 3D structure as predicted during modeling and validation. Additionally, its origin from a well-characterized bacteriophage infecting Gram-positive and Gram-negative bacteria made it a suitable candidate for structure-based ligand interaction analysis. Among all modeled endolysins, this protein exhibited the most promising quality metrics, including the highest percentage of residues in favored regions of the Ramachandran plot, suggesting it as a reliable template for docking simulations. Among the ligands, NAM-L-alanine (NAM-LA) exhibited the strongest binding affinity with a docking score of −7.3 kcal/mol, interacting with key residues such as ASP A:46, THR A:47, and TYR A:61. NAG-NAM also showed strong binding (−6.4 kcal/mol) and shared some contact residues with NAM-LA. Other ligands, including NAG and NAM, displayed moderate binding affinities, with NAG showing interactions with HIS A:45 and NAM interacting with HIS A:45 and TYR A:61. Muramic Acid (MA) and Pentaglycine (PG) both scored −6.0 kcal/mol, with PG showing interactions with HIS A:45 and TYR A:61. Three-dimensional visualizations indicated that the ligands were deeply embedded in the binding pocket, with well-defined hydrogen bonding and minimal unfavorable clashes (Figure 9, Figures S7 and S8, Tables S15 and S16). The native ligand docking results demonstrated strong binding affinities, with NAM-L-alanine exhibiting the lowest binding energy, indicating a highly favorable interaction. Key residues such as ASP A:46 and TYR A:61 formed stable hydrogen bonds with the ligand, consistent with known catalytic and substrate recognition sites in related endolysins. These interactions support the predicted specificity of Endo_LysPALS22_Ec for peptidoglycan fragments and validate the docking methodology as a reliable predictor of biologically relevant binding.

When comparing Endo_LysPALS22_Ec to other endolysins, it demonstrated the strongest affinity for NAM-LA (−7.3 kcal/mol), higher than most other endolysins, including Endo_LysAB-vT2, Endo_LysCSA13, and Endo_LysECD7. This affinity was second only to Endo_Ply113’s interaction with NAM-LADIG (−7.6 kcal/mol). Endo_LysPALS22_Ec’s docking with NAM-LA involved stabilizing interactions with residues such as ASP A:46 and TYR A:61, distinguishing it from other endolysins interacting with different residues. Comparisons with Endo_PlyG_CBD, Endo_PlyG_EAD, Endo_T5-Zn2+, and Endo_T5-Zn2+Ca2+ further emphasized Endo_LysPALS22_Ec’s superior binding to NAM-LA, suggesting it as one of the more robust systems for binding key peptidoglycan fragments, although sharing some structural similarities with other endolysins (Figures S9–S20, Tables S15 and S16).

### 2.8. Molecular Dynamics Simulation

**Figure 10:**
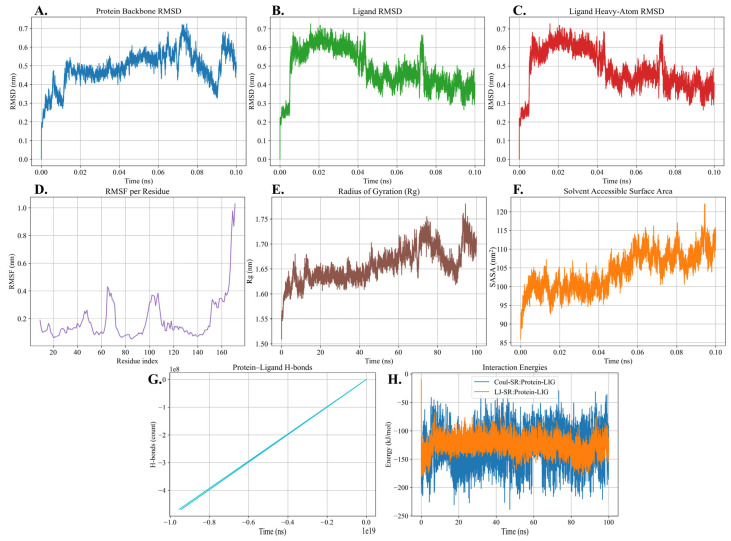
MD simulation results of Endo_LysPALS22_Ec with NAM-L-alanine (NAM-LA). (**A**). Protein Backbone RMSD shows structural stability. (**B**). Ligand RMSD indicates ligand positioning. (**C**). Ligand Heavy-Atom RMSD reflects ligand conformational changes. (**D**). RMSF per Residue highlights residue flexibility. (**E**). Radius of Gyration (Rg) shows protein compactness. (**F**). Solvent Accessible Surface Area (SASA) indicates solvent exposure. (**G**). Protein–Ligand H-bonds count over time. (**H**). Interaction Energies (Coul-SR and LJ-SR) show binding strength.

The backbone RMSD of the protein stabilized after the initial equilibration phase and remained within the range of 0.3–0.7 nm throughout the trajectory (Figure 10A). The ligand RMSD showed a similar stabilization pattern, maintaining values around 0.4–0.6 nm after equilibration (Figure 10B). The heavy-atom RMSD of the ligand also remained stable, fluctuating between 0.3 and 0.6 nm during the simulation (Figure 10C). The RMSF analysis revealed low flexibility across most residues, with average fluctuations below 0.25 nm, while higher fluctuations were observed in specific loop regions and at the terminal residues (Figure 10D). Furthermore, the radius of gyration remained stable, ranging from 1.6 to 1.7 nm during the trajectory (Figure 10E). The solvent accessible surface area (SASA) showed a gradual increase from approximately 95 nm^2^ to 115 nm^2^ (Figure 10F). Protein–ligand hydrogen bond analysis demonstrated the presence of hydrogen bonding interactions that persisted throughout the simulation (Figure 10G). Finally, the interaction energy profile showed consistently negative values for both Coulombic and Lennard-Jones interactions, with Coulombic energies ranging between −100 to −200 kJ/mol and Lennard-Jones energies between −80 to −120 kJ/mol (Figure 10H).

### 2.9. Molecular Verification, Expression, and Purification of Recombinant LysPALS22 in Heterologous Hosts

**Figure 11:**
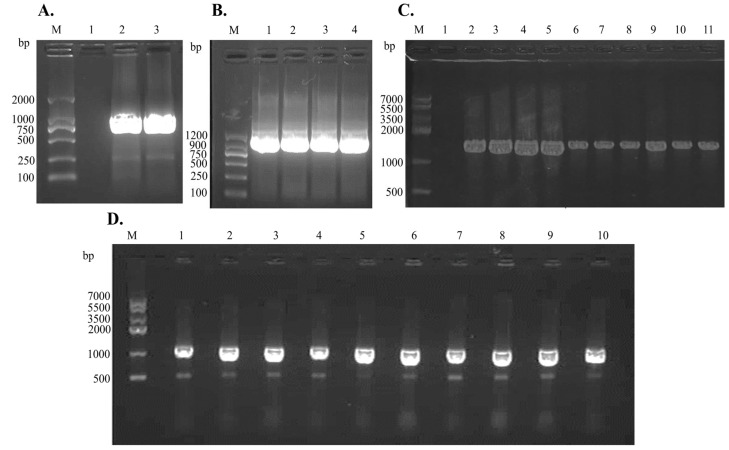
Verification of LysPALS22 cloning and transformants. (**A**) PCR amplification of the 981 bp LysPALS22 ORF for the *E. coli* pET28a construct. (**B**) PCR amplification of the same 981 bp fragment for the *P. pastoris* pPICZαA construct. (**C**) Agarose gel electrophoresis from colonies carrying the recombinant plasmid LysPALS22-pPICZαA. M is the DNA marker, channels 2–11 are the confirmed LysPALS22-pPICZαA genes. The chosen colonies had a DNA fragment that was 1569 bp long, as primer AOX1 was used, so the endolysin gene size (981) was added with the AOX1 gene (588 bp). (**D**) Agarose gel electrophoresis from *E. coli* BL21 colonies carrying the recombinant plasmid LysPALS22-pET28a. M is the DNA marker, channels 1–10 are the confirmed LysPALS22-pET28a genes. The size of each LysPALS22 gene is almost 981 bp.

PCR screening confirmed that every construct generation and strain selection step was successful (Figure 11). A single 981 bp band was amplified from the LysPALS22 open-reading frame and inserted into pET28a (Figure 11A) and pPICZαA (Figure 11B), demonstrating that the gene was intact in both vectors. Colony PCR of ten *E. coli* transformants harboring pET28a-LysPALS22 yielded the same 981 bp fragment in every lane (Figure 11D, lanes 1–10), verifying homogeneous uptake of the plasmid. Likewise, ten *P. pastoris* transformants carrying pPICZαA-LysPALS22 produced a 1569 bp product—representing the 981 bp insert plus 588 bp of the AOX1 junction sequence—in all screened colonies (Figure 11C, lanes 2–11). These four panels confirm correct cloning, vector integrity, and successful transformation in both heterologous hosts.

**Figure 12:**
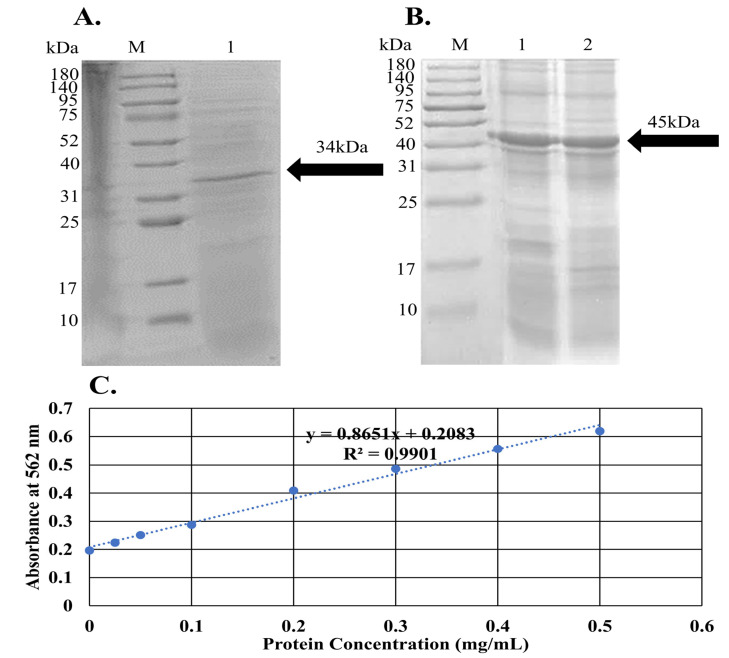
Purification and quantification of recombinant LysPALS22. (**A**) Sodium dodecyl-sulfate polyacrylamide gel electrophoresis (SDS-PAGE) of LysPALS22 expressed in *E. coli*, where channel M indicates the protein marker, channel 1 indicates the ultra-purified endolysin at 34 kDa. (**B**) SDS-PAGE of LysPALS22 expressed in *P. pastoris*, where channel M indicates the protein marker, channel 1 and 2 indicate the ultra-purified endolysin at 45 kDa. (**C**) The standard curve for the BCA (bicinchoninic acid) quantifying protein concentration. The protein concentration is displayed on the X-axis at mg/mL, while the absorbance at 562 nm is displayed on the Y-axis.

Furthermore, affinity-purified recombinant protein from the two expression systems exhibited the expected but host-specific electrophoretic behavior (Figure 12). The *E. coli* preparation resolved as a single dominant band at approximately 34 kDa (Figure 12A, lane 1), matching the calculated mass of His-tagged LysPALS22. In contrast, the *P. pastoris* product migrated at ~45 kDa (Figure 12B, lane 2), indicating an ∼11 kDa shift consistent with post-translational modification in yeast. The comparative analysis of the in silico and wet lab results reveals both congruence and divergence in characterizing the recombinant LysPALS22 proteins expressed in *E. coli* and *P. pastoris*. Bioinformatics analysis of the sequenced Endo_LysPALS22_Ec and Endo_LysPALS22_Pp proteins predicted molecular weights of approximately 35.784 kDa and 45.251 kDa, respectively, with distinct physicochemical properties, including differences in aliphatic index and GRAVY score. These predictions were largely corroborated by the electrophoretic behavior observed in the wet lab, where Endo_LysPALS22_Ec resolved as a band near 34 kDa, consistent with the calculated mass for the His-tagged protein. In contrast, the Endo_LysPALS22_Pp protein exhibited a higher molecular weight of ~45 kDa, aligning with the in silico prediction and suggesting a post-translational modification, likely due to glycosylation in *P. pastoris*, which was not observed in the *E. coli*-expressed protein. These findings highlight the accuracy of the bioinformatics analysis in predicting the molecular features of the recombinant proteins, while emphasizing the impact of host-specific modifications, such as glycosylation in yeast, which were evident in the wet lab results. Moreover, protein concentrations were determined by a bicinchoninic-acid assay whose standard curve displayed excellent linearity (R^2^ = 0.9901; Figure 12C); final yields were 1.31 ± 0.16 mgmL^−1^ (n = 3) for the bacterial preparation and 1.56 ± 0.31 mgmL^−1^ (n = 3) for the yeast preparation. These data confirm that both hosts deliver highly enriched LysPALS22, with *P. pastoris* providing a modestly higher volumetric yield and a glycosylated enzyme form.

#### PNGase F Deglycosylation Confirms Post-Translational Modifications of the *P. pastoris*-Expressed LysPALS22

**Figure 13:**
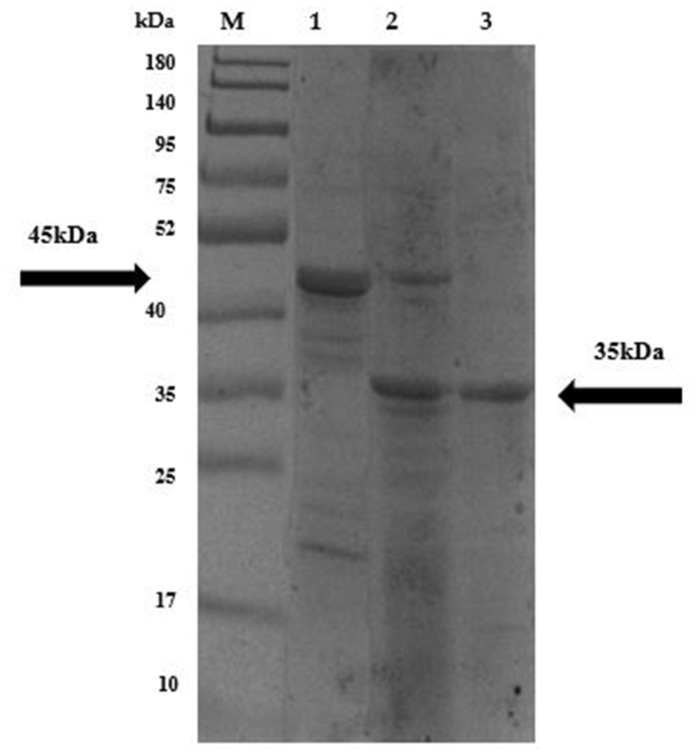
PNGase F deglycosylation of LysPALS22. Lane M, protein marker; Lane 1, *P. pastoris*-expressed LysPALS22 (untreated 45 kDa); Lane 2, PNGase F-treated LysPALS22 showing collapse to the predicted mass (~35 kDa); Lane 3, *E. coli*-expressed LysPALS22, (unglycosylated 34 kDa).

The untreated PNGase F LysPALS22 (*P. pastoris*–expressed) migrated at ~45 kDa, higher than the predicted size, suggesting glycosylation. After PNGase F treatment, the endolysin shifted downward to the predicted molecular mass (~35 kDa), consistent with removal of N-linked glycans (Figure 13). These results confirmed that the recombinant enzyme undergoes yeast-specific N-glycosylation, and deglycosylation resulted in a mobility shift of LysPALS22 from ~45 kDa to ~34 kDa, matching the mass of the unglycosylated protein expressed in *E. coli*.

### 2.10. Antimicrobial Activity of LysPALS22

**Figure 14:**
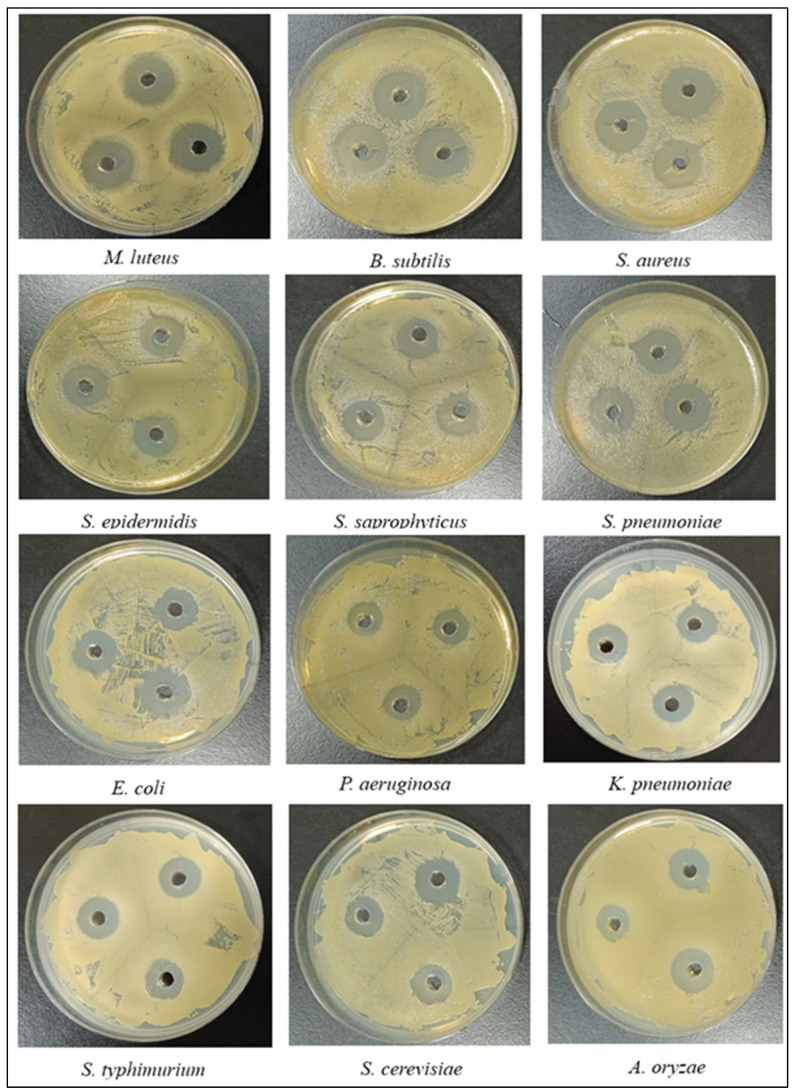
Zone of Inhibition (ZOI) of different Gram-positive and Gram-negative bacteria, yeast, and fungi.

The antimicrobial activity of LysPALS22 against bacterial, fungal and yeast strains compared to Ciprofloxacin (CIP) and Ampicillin (Amp) are summarized in Table 2. The largest inhibition zone was observed against *Micrococcus luteus* ATCC 4698 (22 mm) whereas the smallest was against *Salmonella typhimurium* ATCC 14028 (13 mm) (Figure 14, Table 2). MIC and MBC values were determined in triplicate, and the most frequently observed is reported. The lowest MIC/MBC value (30 μg/mL) was recorded for *M. luteus* ATCC 4698, while the highest MIC/MBC (111 μg/mL) was for *S. typhimurium* ATCC 14028. AMI values for LysPALS22 compared to Ciprofloxacin ranged from 0.88 to 1.1, and compared to Ampicillin from 0.77 to 1.04, indicating greater efficacy for LysPALS22 in certain strains (e.g., *M. luteus*, *S. aureus*) and lesser efficiency in others (e.g., *P. aeruginosa*, *S. epidermidis*). Overall, LysPALS22 exhibited the strongest bactericidal activity against Gram-positive rather than Gram-negative bacteria. Compared with *Aspergillus oryzae* (MIC/MFC; 105 μg/mL), a higher concentration of endolysin LysPALS22 was required to control the growth of *Saccharomyces cerevisiae* (MIC/MFC; 140 μg/mL).

#### Turbidity Reduction and Bacterial Lysis

**Figure 15:**
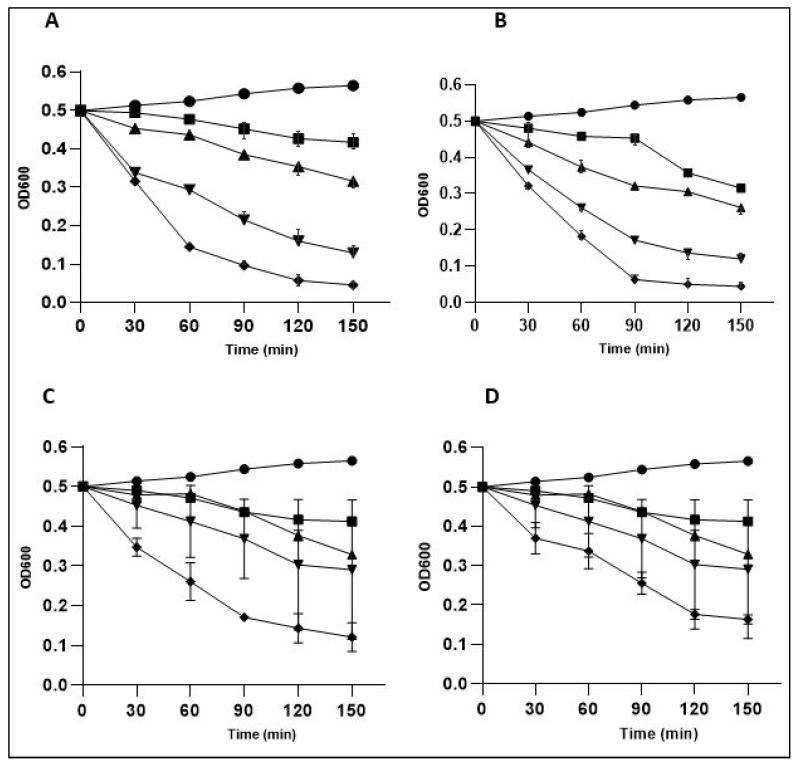
The OD reduction of LysPALS22 at different concentrations against (**A**) *Staphylococcus aureus* ATCC 25923, (**B**) *M. luteus* ATCC 4698, (**C**) *E coli* ATCC 25922, and (**D**) *Klebsiella pneumonia* ATCC 13883. Recombinant LysPALS22 concentrations: 0 μg/mL Control (●), 25 μg/mL (◼), 50 μg/mL (▼), 100 μg/mL (◆), 150 μg/mL (▲). The error bars show the standard error of the mean (n = 3).

**Figure 16:**
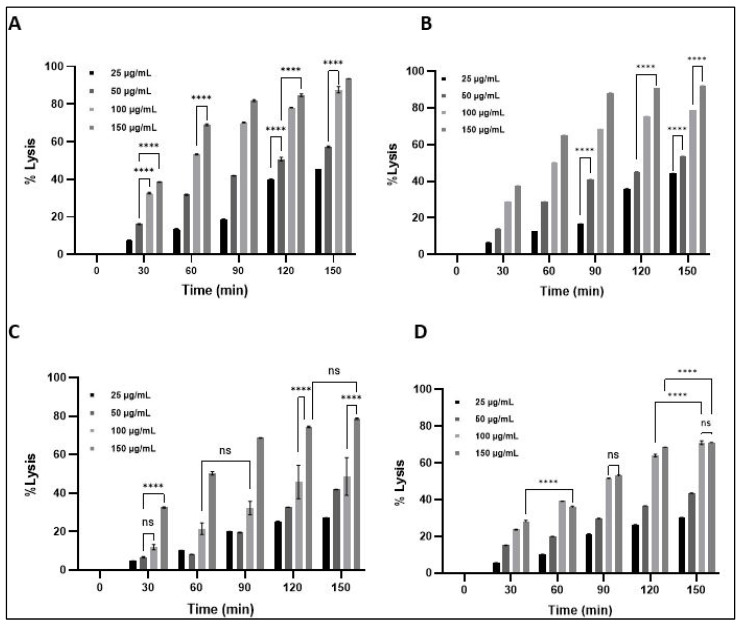
Percent lysis of different bacteria treated with LysPALS22. (**A**) *Staphylococcus aureus* ATCC 25923, (**B**) *M. luteus* ATCC 4698, (**C**) *E coli* ATCC 25922, and (**D**) *Klebsiella pneumonia* ATCC 13883. Bacterial suspensions were treated with different concentrations of LysPALS22 (25, 50, 100, and 150 µg/mL), Data are presented as mean ± SD of triplicate experiments. Statistical significance is indicated (**** *p* < 0.0001; ns, not significant).

The antibacterial activity of LysPALS22 was assessed against *S. aureus*, *M. luteus*, *E. coli*, and *K. pneumoniae* by monitoring bacterial turbidity and calculating percent lysis over time (Figure 15 and Figure 16). LysPALS22 induced a clear concentration- and time-dependent bacteriolytic effect, with Gram-positive strains (*S. aureus*, *M. luteus*) showing a rapid decline in OD600 and >80% at 150 μg/mL within 150 min. In contrast, Gram-negative bacteria (*E. coli*, *K. pneumoniae*) showed slower OD reduction and only partial lysis under the same conditions, with a maximum value of ~40–50%, indicating lower susceptibility. The untreated control maintained stable or slightly increased OD600. These results indicate that LysPALS22 exerts stronger bacteriolytic activity against Gram-positive than Gram-negative bacteria (Figure 15 and Figure 16).

## 3. Discussion

Endolysin enzymes have emerged as promising therapeutic agents amid growing antibiotic resistance concerns [21]. Their distinct mechanisms—targeted and rapid disruption of bacterial cell integrity—position them as effective alternatives or adjuncts to conventional antibiotics [22]. Unlike traditional antibiotics, endolysins demonstrate high specificity, reduced likelihood of resistance development, and substantial efficacy against multidrug-resistant and biofilm-associated pathogens [21]. Despite these advantages, endolysins exhibit significant variability in structural properties, functional domains, solubility profiles, and substrate specificities, necessitating detailed biochemical and computational studies for optimization and therapeutic development.

The present study provides a comprehensive in silico characterization of Endo_LysPALS22 structural, physicochemical, and functional properties. Comparative analysis of diverse endolysins reveals significant variation in molecular weight, stability, hydropathicity, and isoelectric points, likely reflecting evolutionary adaptation to host environments [23]. Endo_LysPALS22 shows strong hydrophilicity, indicated by a negative GRAVY score and low instability index, correlating with favorable solubility and ease of recombinant expression [21]. In contrast, proteins with higher aliphatic indices, such as Endo_LysPA26 and Endo_LysAB-vT2, may offer enhanced thermal stability suitable for industrial applications. Meanwhile, the multiple sequence alignment and conserved domain analysis provided critical insights into functional conservation among endolysins. Conserved catalytic and binding domains, consistently present at the N-terminal regions, reflect evolutionary pressures to retain essential enzymatic activities and specificities. Consistent with [18], who identified diverse functional domain combinations across *Streptomyces* phage endolysins, our study highlights conserved catalytic and binding domains in LysPALS22 and related proteins, underscoring the structural basis for enzymatic activity. These findings also align with a previous study [23]. The conserved residues identified in the consensus regions—particularly serine, lysine, arginine, aspartate, and glutamate—emphasize their essential roles in catalytic activity and structural integrity. The observed structural and sequence conservation, particularly in strategically important regions, suggests that endolysins maintain a core functional architecture essential for their lytic activity despite variations. This implies that subtle variations or mutations within these conserved regions could profoundly affect these enzymes’ specificity, activity, or stability, potentially influencing their antimicrobial efficacy.

Subsequently, evolutionary links between the endolysins were discovered using phylogenetic analysis. Based on functional domains and sequence similarity, the phylogenetic clustering revealed discrete clades and outgroups. Proteins of the same clade most likely have comparable functions and evolutionary predecessors. Endo_LysPALS22_Ec and Endo_LysPALS22_Pp have been discovered to cluster together, indicating that they have independently evolved comparable catalytic properties. This could be due to similar selective pressures in their different bacteriophage–host settings. Furthermore, it is demonstrated that some enzymatic processes are independently conserved across various evolutionary lineages by the existence of metalloprotease-related endolysins in distinct clades. Functional diversification among the endolysins under study is thus further highlighted by thoroughly examining domain architecture and conserved motifs. These proteins’ predominant amidase, lysozyme-like, and CHAP domains highlight their crucial function in bacteriolytic activities [23]. The presence of specific motifs such as SH3 and PGRP, frequently linked to bacterial peptidoglycan recognition, indicates potential specificity toward specific bacterial hosts or cell-wall structures [24]. Moreover, domain variations, such as the unique secretion signals identified in Endo_LysPALS22_Pp for *P. pastoris* expression, point toward host-specific adaptations to ensure efficient enzyme processing and secretion. This diversity of domain organization and motif presence provides a platform for rational design and engineering novel endolysins with tailored antibacterial or anti-biofilm properties. Endo_LysPALS22_Ec’s solubility analysis and structural comparison provided crucial information on its biophysical properties [25]. The structural alignment revealed striking similarities between the models anticipated using various computational techniques. It emphasizes the reliability of these structures for ligand binding assessments and functional research. Marginally lower solubility predictions relative to the *E. coli* soluble protein average indicate slight challenges in recombinant expression or formulation [26]. However, regions exhibiting positive deviations, specifically residues such as lysine and glutamic acid, could be manipulated to enhance solubility and stability, potentially improving therapeutic formulation. Moreover, regions with pronounced negative charges could be critical for interaction stability and enzymatic activity, particularly in response to environmental conditions like pH and ionic strength fluctuations. On the one hand, interaction energy and charge distribution analyses highlighted how the stability and function of Endo_LysPALS22_Ec could significantly vary under different physicochemical conditions. The observed reduction in repulsive interactions with increased ionic strength suggests potential functional stabilization under physiological or therapeutic conditions, typically featuring moderate ionic strengths [27]. This stabilization might enhance enzyme activity and antimicrobial efficacy at specific physiological pH ranges. Meanwhile, charge sensitivity to pH variations underscores the importance of optimizing enzyme activity conditions, especially when applied in diverse therapeutic or industrial contexts. Tailoring environmental conditions to exploit the identified attractive interactions could enhance the overall performance and specificity of the enzyme.

The molecular docking analysis provided a comprehensive understanding of ligand-binding capabilities and interaction dynamics of Endo_LysPALS22_Ec. The exceptional affinity of NAM-L-alanine (NAM-LA) to this endolysin indicates a particular and potent binding site, likely reflecting biological relevance toward peptidoglycan-derived substrates [23]. The observed strong binding affinity of LysPALS22 towards peptidoglycan fragments parallels the PG-degrading activities reported for experimentally validated endolysins such as LL35lys and Tb42lys [18], reinforcing the functional relevance of our docking predictions. Strong interactions with residues such as ASP A:46, THR A:47, and TYR A:61 suggest targeted mutagenesis at these sites could enhance or alter substrate specificity and catalytic efficiency [17]. The consistently moderate-to-strong binding affinities for other ligands, particularly NAG-NAM and muramic acid derivatives, imply broader substrate recognition capabilities, advantageous for targeting diverse bacterial species or mixed microbial populations [17,23]. These insights could significantly inform enzyme engineering strategies, enabling the creation of novel endolysin variants with tailored antimicrobial activities [17,23]. Additionally, future studies will incorporate molecular dynamics simulations to complement the docking results and experimental data to evaluate the dynamic stability and flexibility of LysPALS22 and its complexes, including RMSD, RMSF, SASA, Rg, and hydrogen bonding analyses [20,28]. In addition, recent studies, such as [29], have demonstrated that fusion of antimicrobial peptides like cecropin A to lysins can significantly enhance their antibacterial activity against Gram-negative pathogens, including *Acinetobacter baumannii*. Such engineering approaches address the challenge of outer membrane penetration, which is a critical barrier for lysin efficacy in Gram-negative bacteria. Our structural and expression analyses of LysPALS22 provide a foundational framework that could be leveraged in similar peptide-fusion strategies to optimize antimicrobial potency and broaden the enzyme’s spectrum [30]. Furthermore, the molecular dynamics simulation demonstrated that the protein–ligand complex achieved a stable conformation after equilibration, with backbone RMSD (0.3–0.7 nm) and ligand RMSD (0.3–0.6 nm) remaining steady, indicating structural integrity and a consistent binding pose. RMSF values showed limited flexibility outside of terminal loops, while the radius of gyration (1.6–1.7 nm) confirmed overall compactness. A gradual SASA increase (~95–115 nm^2^) suggested minor surface adjustments without structural disruption. Crucially, persistent hydrogen bonds and consistently favorable interaction energies (Coulombic −100 to −200 kJ/mol; Lennard-Jones −80 to −120 kJ/mol) highlighted stable and energetically favorable binding, driven by complementary electrostatic and van der Waals interactions.

The molecular verification purification results collectively confirmed that the LysPALS22 open-reading frame can be functionally expressed in *E. coli* and *P. pastoris* host [31]. Similarly to [18], who demonstrated distinct antimicrobial spectra for different endolysins against Gram-positive and Gram-negative ESKAPE relatives, our study’s experimental validation of LysPALS22 expression in *E. coli* and *P. pastoris* lays the groundwork for future functional assays to determine LysPALS22’s activity and spectrum. In line with Kai-Sheng Shen et al. (2022), who characterized LysJN01 from phage JN01 [32], our findings support the use of *E. coli* and *P. pastoris* as robust hosts for producing functional endolysins. Both studies highlight the successful recombinant expression of endolysins, with LysJN01 exhibiting a molecular mass of approximately 17.2 kDa and our LysPALS22 endolysin expressed in *E. coli* and *P. pastoris* showing molecular weights of ~34 kDa and ~45 kDa, respectively, consistent with bioinformatics predictions. From a production perspective, *E. coli* offers rapid, cost-effective intracellular expression (1.31 ± 0.16 mg/mL), while *P. pastoris* facilitates secretion with higher yields (1.56 ± 0.31 mg/mL), simplifying purification and reducing contaminants. Thus, these systems provide complementary platforms: *E. coli* for rapid mutagenesis and *P. pastoris* for scalable production of a potentially more stable enzyme. Future work should focus on assessing catalytic efficiency differences due to glycosylation, stability profiles, and optimization of induction conditions. Supporting these findings, [33] reported a *Salmonella* phage endolysin produced in *E. coli* with high yield, broad Gram-negative activity, and thermal stability; notably, its combination with chitosan enabled spontaneous bacterial lysis, highlighting opportunities for therapeutic enhancement. Together, these studies underscore the promise of phage endolysins like LysPALS22 for antimicrobial development.

In positioning LysPALS22 relative to clinically advanced endolysins such as CF-301 (Exebacase) and SAL200, it is important to recognize that our study is based on in silico analyses, whereas these clinical candidates have undergone extensive experimental and preclinical validation. Nevertheless, the computational characterization of LysPALS22 provides valuable insights into its potential. For example, the predicted structural stability, favorable Ramachandran statistics, and accessible catalytic residues suggest that LysPALS22 possesses the fundamental features required for robust enzymatic activity. These attributes align with properties observed in endolysins that have progressed to clinical evaluation, indicating that LysPALS22 may represent a promising scaffold for further development. At the same time, it is clear that in silico predictions cannot fully substitute for empirical validation. Key aspects such as lytic spectrum, activity in physiological conditions, immunogenicity, and pharmacokinetics remain to be established experimentally. Thus, while LysPALS22 appears to compare favorably at a structural and theoretical level with well-characterized clinical candidates, further in vitro and in vivo studies are essential to confirm its potential and to identify areas where optimization may be required.

LysPALS22 demonstrated potent antimicrobial activity, particularly against Gram-positive bacteria like *Micrococcus luteus*, which showed the largest inhibition zone (22 mm) and lowest MIC/MBC (30 μg/mL). In contrast, Gram-negative strains such as *Salmonella typhimurium* were less susceptible, with higher MIC/MBC values (111 μg/mL) and smaller inhibition zones. Although MIC/MBC values of LysPALS22 were higher than usual, they are consistent with reported ranges for other endolysins, such as LysECD7 (>1000 µg/mL) and LysSS (≥150–200 µg/mL). LysPALS22 showed strain-dependent activity when compared to Ciprofloxacin and Ampicillin, demonstrating superior effectiveness against M. luteus (PAI > 100%, AMI > 1) but inferior activity against *P. aeruginosa* (PAI < 100%, AMI < 1). These results suggest LysPALS22’s potential as an alternative antimicrobial agent, particularly against Gram-positive bacteria. However, its reduced effectiveness against Gram-negative strains underscores the need for further optimization.

Turbidity and lysis assays further confirmed the enzyme’s stronger, concentration-dependent bacteriolytic effect on Gram-positive bacteria, achieving over 80% lysis, while Gram-negative strains exhibited only partial lysis (~40–50%). This study was conducted using ATCC reference strains, which are antibiotic-susceptible quality control organisms. The ZOI values in Table 2 confirm that the ATCC reference strains are susceptible to Ciprofloxacin and Ampicillin, indicating no antibiotic resistance. Although these results provide proof-of-concept for LysPALS22 activity, future studies will be needed to evaluate its efficacy against multi-drug-resistant clinical isolates.

The findings presented in this study lay important groundwork for future research into endolysins, particularly in understanding and harnessing their antimicrobial and anti-biofilm potentials. Comprehensive characterization of physicochemical properties, conserved domains, structural conformations, solubility profiles, and ligand-binding capabilities will aid in rational engineering of endolysins with improved stability, specificity, and therapeutic efficacy. Furthermore, this study provides a robust computational framework to rapidly screen, predict, and optimize endolysin properties, significantly expediting the discovery and development of potent enzybiotics. Future research could leverage these insights to create tailored antimicrobial solutions, combating multidrug-resistant bacterial strains and biofilm-associated infections with improved efficiency and precision.

## 4. Methods and Materials

### 4.1. Sequence Assembly and Contig Generation Using BioEdit

The forward and reverse sequence read files were obtained from the sequencing company for both *Escherichia coli (E. coli)* and *Pichia pastoris (P. pastoris)* constructs. To ensure sequence accuracy and eliminate potential sequencing errors, contig assembly was performed using the CAP3 Contig Assembly Program in BioEdit version 7.7.1. The raw sequencing reads were first imported into BioEdit, and the CAP3 algorithm was employed to merge overlapping regions of the forward and reverse reads, generating high-confidence consensus sequences. This approach facilitated error correction by resolving mismatches, ensuring proper alignment of overlapping regions, and removing low-quality bases at the sequence ends. The resulting assembled contigs were verified for sequence integrity before proceeding to downstream bioinformatics analyses.

### 4.2. Protein Translation and Open Reading Frame Selection

After obtaining the consensus sequences, the next step involved translating the DNA sequences into their corresponding protein sequences. Translation was performed using the Expasy Translate tool [34], which allows the conversion of nucleotide sequences into amino acid sequences based on the standard genetic code. The input comprised the finalized contig sequences from *E. coli* and *P. pastoris*. The tool was executed with default parameters to generate all six possible reading frames. Among these, the most extended open reading frame (ORF) was selected for each sequence, as it represents the most likely functional protein-coding region. This step ensured that the correct reading frame was chosen for downstream structural and functional analyses.

### 4.3. Selection of Endolysins from Literature and Sequence Retrieval

In addition to our two endolysins, a systematic search was performed to identify bacteriophage endolysins with experimentally validated antibacterial activity. Literature screening was conducted using PubMed and Google Scholar with combinations of the following keywords: “endolysin,” “phage lysin,” “bacteriophage lytic enzyme,” “peptidoglycan hydrolase,” “antimicrobial activity,” “Gram-negative bacteria,” and “Gram-positive bacteria.” Relevant information was extracted from original research articles, reviews, and experimental studies [17,35,36,37,38,39,40], and cross-verified with specialized microbial genome repositories. The inclusion criteria were (i) experimental evidence of antibacterial or lytic activity reported in the literature, (ii) availability of full-length amino acid sequences in the NCBI Protein and GenBank databases [41] or the RCSB Protein Data Bank (PDB), and (iii) representation of diverse enzymatic classes (e.g., glycosidases, amidases, and endopeptidases) to capture structural and evolutionary diversity among bacteriophage lysins active against Gram-negative bacteria (GNB). The exclusion criteria included (i) sequences without publicly deposited data in NCBI or PDB, (ii) redundant or highly similar sequences from the same phage, and (iii) mycobacteriophage endolysins, which were excluded due to their distinct host range and lack of comprehensive sequence data relevant to this analysis. As a result of this strategy, 10 endolysins were identified through literature screening with sequence data available in the NCBI Protein and GenBank databases [41]. These included Endolysin_LysH5, Endolysin_LysECD7, Endolysin_LysB4, Endolysin_LysPA26, Endolysin_LysCSA13, Endolysin_LysZX4-NCA, Endolysin_LysP108, Endolysin_LysAB-vT2, Endolysin_Ply113, and Endolysin_LysAm24.

In addition, four other endolysins were selected from a recent study by Arina G. Arakelian et al. (2024) [17], namely Endo_T5-Zn2+, Endo_T5-Zn2+/Ca2+, Endo_PlyG_EAD, and Endo_PlyG_CBD, as these endolysin binding interactions were validated via molecular docking analysis. Together with our two endolysins, this yielded a set of 16 proteins. The amino acid sequences of all selected endolysins were retrieved from the NCBI Protein database [41] or the RCSB Protein Data Bank (PDB) by searching their GenBank accession numbers, Protein IDs, or PDB IDs. A short summary of these proteins, including their names, accession numbers, and bacteriophage origin, is provided in Table 3, while the detailed table is given in Supplementary Table S1.

### 4.4. Physicochemical Properties Analysis

The physicochemical properties of the endolysin proteins were analyzed using TBtools v2.141 [42,43], a widely used bioinformatics software for sequence analysis and visualization. The Protein Parameter Calculation tool, specifically the Prot-Param-based module in TBtools, was utilized to compute a range of essential physicochemical properties of the proteins. These properties are critical for understanding the structure–function relationships of the proteins and their stability under various conditions. The Prot-Param-based tool was selected for its ability to compute multiple physicochemical parameters. This tool efficiently calculates the number of amino acids (which indicates the protein’s length), MW, theoretical isoelectric point (pI), instability index, aliphatic index, and GRAVY of the given protein sequences. Moreover, bar charts were created to enhance the visualization and understanding of these various physicochemical properties.

### 4.5. Multiple Sequence Alignment and Phylogenetic Analysis of Endolysins

The MSA was performed using CLC Sequence Viewer software [44] v8.0. The alignment aimed to identify conserved motifs, catalytic residues, and sequence variations among the selected endolysins. The ClustalW algorithm was used as the alignment method, ensuring accurate comparison by aligning homologous residues while minimizing gaps. The aligned sequences were visually inspected to confirm proper alignment and assess conserved regions across different endolysins. To further deeply visualize the degree of sequence conservation and variability within the consensus region (amino acids 522–632), sequence logos were generated using the WebLogo web-based application [45]. WebLogo graphically represents conserved residues as larger letters, highlighting key amino acids such as serine (S), lysine (K), arginine (R), aspartate (D), glutamate (E), glycine (G), proline (P), and alanine (A), which are important for enzymatic function and protein stability.

Moreover, a phylogenetic tree was constructed using the aligned sequences to further investigate the evolutionary relationships among these bacteriophage-derived lytic enzymes. The tree was generated in CLC Sequence Viewer employing the Neighbor-Joining (NJ) method with 1000 bootstrap replications to assess the statistical reliability of the clustering. The evolutionary distances between different endolysins were calculated based on Poisson correction models, and the tree topology was analyzed to determine the clustering of closely related sequences. The resulting phylogenetic tree provided insights into the divergence of endolysins, their evolutionary origins, and potential functional similarities based on clustering patterns.

### 4.6. Domain and Motif Analyses

After constructing the tree, domain analysis was conducted using InterProScan with default parameters. InterProScan provides a comprehensive functional annotation of protein sequences by identifying potential protein domains, families, and functional sites. Following the domain analysis, motif analysis was performed using MEME Suite [46], which is designed to identify conserved motifs across the given sequences. For motif identification, the Motif Site Distribution parameter was adjusted to OOPS (exactly one site per sequence), the number of motifs were set to 10, and other parameters were kept as default. The identified motifs were then visualized to understand their distribution and conservation within the sequences. To further enhance the interpretation of the results, domain and motif data were visualized using TBtools software.

### 4.7. Protein Structure Prediction and Comparison

The three-dimensional (3D) structures of all sixteen endolysins were initially predicted using the ExPASy Swiss-Model “Build Model” server [47,48,49]. For Endo_LysPALS22_Ec, three independent modeling resources were employed to assess structural consistency and reliability: Swiss-Model (Endo_LysPALS22_Ec), AlphaFold Colab (EndoLys_PALS22_Ec_AlphaFold) [50,51], and the EMBL-EBI AlphaFold Protein Structure Database (AF-A0A855GMK5-F1-model_v4). For AlphaFold-based predictions, the per-residue confidence scores (predicted Local Distance Difference Test; pLDDT) were directly obtained from the respective servers. pLDDT values greater than 90 were interpreted as very high confidence, 70–90 as high confidence, 50–70 as low confidence, and <50 as very low confidence. All three models were subjected to an extensive validation workflow to identify the most reliable structure for downstream applications. Structural assessments were performed using the SAVES v6.0 server (https://saves.mbi.ucla.edu/ accessed on 25 June 2025), which integrates multiple independent quality evaluation tools. ERRAT was applied to assess the overall reliability of non-bonded atomic interactions, with higher scores reflecting fewer errors and improved atomic environments. VERIFY3D was used to evaluate the compatibility between the 3D atomic model and its own amino acid sequence, where ≥80% of residues with an average 3D-1D score ≥0.1 indicates a reliable model. Stereochemical quality was further examined using PROCHECK, which generates Ramachandran plots to evaluate the distribution of φ (phi) and ψ (psi) backbone dihedral angles, with the proportion of residues in favored, allowed, and disallowed regions serving as an indicator of geometric accuracy. In addition, the predicted structures were validated using the MolProbity server (http://molprobity.biochem.duke.edu/, accessed on 25 June 2025), which provides a comprehensive assessment of stereochemical quality, including all-atom clashscores, MolProbity global scores, rotamer outliers, Ramachandran outliers, CaBLAM outliers, and bond/angle deviations. Percentile ranks were reported relative to high-resolution crystal structures in the Protein Data Bank (PDB). By integrating the results from ERRAT, VERIFY3D, PROCHECK Ramachandran statistics, MolProbity stereochemical parameters, and AlphaFold pLDDT scores, the Swiss-Model structure of Endo_LysPALS22_Ec was identified as the most balanced and reliable prediction. This structure was therefore selected as the reference for molecular docking analysis, while the AlphaFold Colab model (Endo_LysPALS22_Ec_AlphaFold) was used as the reference for structural comparisons with other endolysins, as it represented nearly the entire sequence of the respective protein.

Initially, the structural comparison of the model structures was performed. Subsequently, structural comparisons of the sixteen predicted endolysins, including the Endo_LysPALS22_Ec from Swiss-model and AF-A0A855GMK5-F1-model_v4, were conducted using UCSF ChimeraX (version 1.9) [52,53], with Endo_LysPALS22_Ec_AlphaFold serving as the reference structure. Default secondary structure assignments were generated automatically where PDB files lacked explicit HELIX or SHEET records. The MatchMaker utility was then employed to superimpose target chains onto the reference structure. By default, MatchMaker applies a Needleman–Wunsch sequence alignment using the BLOSUM-62 matrix, incorporates secondary structure information (SS-fraction ≈ 0.3), and refines the alignment iteratively. Poorly fitting residues were pruned to focus on the best-aligned core subset of residues, after which ChimeraX reported the number of pruned atom pairs, the corresponding Root Mean Square Deviation (RMSD), and the “all pairs” RMSD. Noticeably, structural alignments were performed separately for enzymatically active domains (EADs) and cell wall-binding domains (CBDs) rather than considering whole-protein values. Domain boundaries were defined based on Pfam annotations, and RMSD as well as alignment scores were recorded independently for each domain category. This domain-focused approach highlighted conserved catalytic cores (EADs) and structural variability among CBDs, thereby providing a more functionally meaningful comparison across the endolysin set.

### 4.8. Protein Solubility, Interaction Energy, and Charge Analysis

The solubility and sequence feature analysis of the protein “Endo_LysPALS22_Ec” was conducted using the Protein-Sol web tool (https://protein-sol.manchester.ac.uk/). The protein sequence in FASTA format was submitted to the tool, which predicted the protein’s solubility, charge, folding propensity, disorder, and other sequence-based features. The predicted scaled solubility (QuerySol) was calculated and compared to the population average (PopAvrSol), using data from Niwa et al., 2009 [54]. Additional sequence features were calculated over a sliding 21-amino-acid window, including amino acid compositions, folding propensities, disorder propensities, and hydropathy. The results were visualized through various graphical representations, including solubility values, deviations from the population average, windowed charge scores, and windowed fold propensity scores.

Furthermore, the ‘Heatmap’ tool from the Protein-Sol web tool was used to evaluate the interaction energy and charge per amino acid of the protein “Endo_LysPALS22_Ec” across varying ionic strengths (0.005 M to 0.3 M) and pH values (2.0 to 8.0). The interaction energy was calculated in Joules per amino acid (J per aa), with positive values indicating repulsive interactions and negative values indicating attractive interactions. The charge per amino acid was calculated by considering the ionization of acidic and basic residues at each pH. Furthermore, heatmaps were generated for interaction energy and charge, with ionic strength on the y-axis and pH on the x-axis. The energy heatmap was color-coded from red (higher energy) to green (lower energy), while the charge heatmap ranged from dark blue (negative charge) to red (positive charge). In addition, graphs were generated to show the charge profile of the query protein and Fab set at ionic strengths of 0.05 M, 0.15 M, and 0.3 M, with charge per amino acid plotted against pH.

### 4.9. Molecular Docking of Ligands with Endolysins

For the docking analysis, we utilized PyRx (v. 0.8) [55] (Python-based Virtual Screening Tool) in conjunction with the AutoDock Vina algorithm to dock a series of ligands with various endolysin proteins. AutoDock Vina (v. 1.5.7) employs a knowledge-based empirical scoring function that combines steric, hydrophobic, hydrogen-bond, and electrostatic terms. Complexes were ranked by the Vina affinity score (kcal mol^−^^1^), with the lowest (most negative) value as the best pose. The ligands were selected from the study of Arina G. Arakelian et al. (2024) [17]. Ligand structures were obtained from PubChem [56], including N-Acetylglucosamine (NAG; CID: 24139), N-Acetylmuramic Acid (NAM; CID: 5462244), NAG-NAM (CID: 72210857), NAM-L-alanine (NAM-LA; CID: 10970945), NAM-L-alanyl-D-isoglutamine (NAM-LADIG; CID: 451714), Pentaglycine (PG; CID: 81537), and Muramic Acid (MA; CID: 441038) (Table S12). These structures were optimized using PyRx’s structure preparation tools to ensure suitability for docking. The protein targets included Endo_LysAB-vT2, Endo_LysCSA13, Endo_LysECD7, Endo_LysP108, Endo_LysPA26, Endo_LysPALS22_Ec, Endo_LysZX4-NCA, and Endo_Ply113. These proteins were selected based on our evolutionary/phylogenetic tree result. One protein from each sister clade and the outgroup proteins were selected as targets. The protein structures were preprocessed by removing water molecules and heteroatoms, followed by protonation to prepare them for docking, and then blind docking was performed. Using AutoDock Vina, PyRx searched for optimal ligand conformations within the proteins’ active sites by maximizing binding affinity. For each pose, binding energy values were calculated. Multiple conformations were generated and ranked by binding affinity, with the conformation showing the lowest binding energy selected for further analysis. To visualize and interpret the interactions between ligands and proteins, the docking results were analyzed using Discovery Studio Visualizer v2.5.5, highlighting key interactions such as hydrogen bonds, hydrophobic contacts, and other stabilizing molecular interactions within the complexes.

### 4.10. Molecular Dynamics Simulation Analysis

All molecular dynamics (MD) simulations were carried out using GROMACS (CUDA-enabled build) with the CHARMM36 (July 2022) force field for the protein. The ligand NAM-L-alanine (NAM-LA) was parameterized with CGenFF: its MOL2 file (hydrogens added in Avogadro and bond orders corrected with sort_mol2_bonds.pl) was submitted to the CGenFF server and converted to GROMACS format with cgenff_charmm2gmx_py3.py, yielding lig.prm and lig.itp, which were incorporated into the system topology. The Endo_LysPALS22_Ec protein coordinates were prepared with pdb2gmx (CHARMM36, default protonation/patching), and the protein–ligand complex was assembled by merging the processed protein and NAM-LA coordinate files to generate complex.gro. The complex was then placed in a truncated dodecahedral periodic box with at least 1.0 nm padding from the solute and solvated with SPC216 water. Counter-ions (Na^+^/Cl^−^) were added with genion to neutralize the system charge. In addition to this, energy minimization was performed using the steepest-descent algorithm until convergence (maximum force ≤ 1000 kJ mol^−1^ nm^−1^) to remove steric clashes. Equilibration was performed in two phases with position restraints (protein heavy atoms from pdb2gmx and ligand heavy atoms from genrestr, force constant 1000 kJ mol^−1^ nm^−2^). First, an NVT ensemble equilibration was run at 300 K using a velocity-rescaling thermostat, followed by NPT equilibration at 300 K and 1 bar using the Parrinello–Rahman barostat to equilibrate system density under periodic boundary conditions. Subsequently, a 100 ns production run was performed without restraints. A 2 fs integration step was used, with all hydrogen-containing bonds constrained via LINCS. Long-range electrostatics were treated with the Particle Mesh Ewald (PME) method, and van der Waals and real-space Coulomb interactions were calculated with cutoffs consistent with CHARMM36 recommendations. Simulations were executed on GPU hardware with multi-threaded CPU support (OpenMP) for PME and bonded interactions.

Finally, the trajectory post-processing included recentering and rewrapping around the protein (trjconv-center-pbc mol-ur compact) followed by least-squares superposition onto the protein backbone (-fit rot+trans) to remove overall translation and rotation. Analyses were carried out with standard GROMACS tools on the centered, fitted trajectory: protein backbone RMSD and ligand RMSD (all-atom and heavy-atom), per-residue RMSF, radius of gyration (Rg), solvent-accessible surface area (SASA), and protein–ligand hydrogen-bond counts. Non-bonded interaction energies (short-range Coulombic and Lennard-Jones components) between protein and ligand were obtained via a rerun calculation on the production trajectory and extracted with gmx energy. All time-series are reported in nanoseconds; figures were generated from the .xvg outputs using custom Python/Matplotlib scripts to produce publication-quality plots.

### 4.11. Experimental Validation

#### 4.11.1. Bacterial Strains and Reagents

*E. coli* BL21 (DE3) competent cells and the X33 strain were obtained from Beijing Zhuangmeng International Biogene Technology Co., Ltd. (Beijing, China) Antibiotics ampicillin and kanamycin, as well as Isopropyl β-D-1-thiogalactopyranoside (IPTG), were sourced from Biotopped Company, Beijing, China. The SDS-PAGE kit, loading buffer, DNA markers, and plasmids pET-28a and pPICZαA were purchased from the Biogenomics Division of Beijing Zhuangmeng International Biogene Technology Co., Ltd. Protein markers were acquired from Thermo Fisher Scientific (Waltham, MA, USA). At the same time, OMEGA supplied DNA T4 Ligase and restriction enzymes. All bacterial strains were ATCC reference strains, widely used as quality control organisms for antimicrobial testing. These strains are antibiotic-susceptible and are not known to carry clinically relevant resistance traits (Table 4).

#### 4.11.2. Cloning and Expression of the Endolysin LysPALS22 in *E. coli* and P. pastoris

To validate the function of the LysPALS22 gene, we cloned it into two different host systems, *E. coli* and *P. pastoris*. The gene sequence of LysPALS22 (NCBI ID: OF299921) was used to design primers. Both forward and reverse primers were designed to target the N-terminal and C-terminal regions of the gene, incorporating restriction sites (NcoI/XhoI and EcoRI/NotI) and a 6x His tag at the N-terminus for recombinant expression in pET28a and a 6x His tag at the C-terminus for recombinant expression in pPICZαA for *E. coli* and *P. pastoris* transformations, respectively (Table 5). The DNA for the amplicon was extracted from the endolysin LysPALS22 of bacteriophage PALS, and PCR was performed using standard protocols, with slight modifications in the annealing temperature (58 °C).

Each amplicon was digested with its restriction enzymes, ligated into the corresponding vector, and the ligation reaction was carried out overnight at 16 °C using T4 DNA ligase. Heat shock transformed the recombinant plasmids into competent *E. coli* cells. Transformants were selected on LB-kanamycin plates (for pET28a) and low-salt LB–zeocin plates (for pPICZαA), with recombinant clones identified via colony PCR. For bacterial expression, the confirmed pET28a-*LysPALS22* plasmids were introduced into *E. coli* BL21. Cultures grown in LB medium at 37 °C were induced at an OD_600_ ≈ of 0.8 with 0.5 mM IPTG, followed by shaking (220 rpm, 14 h, 37 °C). Cells were harvested by centrifugation (8000 rpm, 20 min), washed with PBS, lysed by sonication (20 min), and clarified by centrifugation (8000 rpm, 10 min, 4 °C). The supernatant was purified and analyzed using SDS-PAGE. Meanwhile, for yeast expression, pPICZαA-LysPALS22 plasmids were linearized with SacI, ethanol-precipitated, and electroporated into competent *P. pastoris* X33 cells. Zeocin-resistant transformants were screened on YPD plates by colony PCR, grown in small-scale cultures, and induced with 1% methanol. Culture supernatants containing the secreted enzyme were purified and analyzed by SDS-PAGE.

#### 4.11.3. Protein Purification, SDS-PAGE Analysis, and Protein Quantification

His-tagged endolysin obtained from *E. coli* and *P. pastoris* was purified by nickel-affinity chromatography; SDS PAGE confirmed the molecular sizes of the resulting ultra-purified proteins. A bicinchoninic acid (BCA) assay was performed for protein quantification: a 0.5 mg mL^−1^ protein standard was prepared by diluting 10 µL of stock standard in 100 µL PBS and stored at −20 °C. In a 96-well plate, 0, 0.1, 0.25, 0.5, and 0.75 mg mL^−1^ standards and 20 µL of each endolysin sample were mixed with 200 µL BCA working reagent and incubated for 30 min at 37 °C. Absorbance at 562 nm was recorded with a microplate reader, and total protein content was calculated from the resulting standard curve.

### 4.12. PNGase F Denaturing Assay

Purified LysPALS22 expressed in *Pichia pastoris* (1–20 µg) was denatured in 1 µL of glycoprotein denaturing buffer (10X) at 100 °C for 10 min, chilled, and then centrifuged for 10 s. For PNGase F reactions, NP-40 was added to 1% (*v*/*v*) final to neutralize SDS. Then samples were incubated with PNGase F (New England Biolabs, Ipswich, MA, USA) in 1× GlycoBuffer 2 at 37 °C for 1 h. Parallel reaction with the untreated PNGase F endolysin was also included. Then the mobility shift on 12% SDS-PAGE gel was analyzed. As a reference, LysPALS22 endolysin expressed via *E. coli* was also run on the SDS gel.

### 4.13. Antibacterial Activity Assays for LysPALS22

#### 4.13.1. Well Diffusion Assay (ZOI)

Antibacterial activity of LysPALS22 was evaluated by the well diffusion method. Sample organisms included six Gram-positive bacteria (*Micrococcus luteus*, *Bacillus subtilis*, *Staphylococcus aureus*, *S. saprophyticus*, *S. pneumonia*, *S. epidermidis*), four Gram-negative bacteria (*Escherichia coli*, *Pseudomonas aeruginosa, Salmonella typhimurium, Klebsiella pneumoniae)*, one yeast (*Saccharomyces cerevisiae)*, and one fungus (*Aspergillus oryzae)*. Ciprofloxacin (10 μg/mL), Ampicillin (10 μg/mL), and miconazole (10 μg/mL) served as positive control, while deionized water was used as a negative control. The wells (6 mm) were filled with 10 μL purified LysPALS22, allowed to diffuse for 15–20 min, and incubated at 37 °C for 24–48 h. ZOIs were measured and compared with controls. All assays were performed in six replicates.

#### 4.13.2. Minimum Inhibitory Concentration (MIC) and Minimum Bactericidal/Fungicidal Concentration (MBC/MFC) Assays

A modified dilution method was used for the determination of MIC and MBC. Different concentrations of LysPALS22 were prepared in sterile nutrient broth (1 mL/tube) and inoculated with bacterial, fungal, and yeast cultures. Bacterial tubes were incubated at 37 °C for 24 h, and fungal tubes at 25 °C for 5 days, and then growth checked by turbidity. The lowest concentration showing no turbidity was further streaked onto agar plates to confirm bactericidal/fungicidal activity. Tubes were replenished with fresh broth, re-incubated under the same conditions, and both tubes and agar plates were examined for growth. All assays were performed in three replicates.

The AMI and PAI were determined using the following formula.

$$\mathrm{AMI}=\frac{\mathrm{ZOI}\;\mathrm{of}\;\mathrm{Purified}\;\mathrm{Endolysin}}{\mathrm{ZOI}\;\mathrm{of}\;\mathrm{Antibiotic}}$$

AMI values > 1 indicate recombinant endolysin is more active than the standard drug (antibiotic), while the AMI < 1 indicates recombinant endolysin is less active than the standard drug.

$$\mathrm{PAI}=\frac{\mathrm{ZOI}\;\mathrm{of}\;\mathrm{Purified}\;\mathrm{Endolysin}}{\mathrm{ZOI}\;\mathrm{of}\;\mathrm{Antibiotic}}\times 100$$

Value > 100 shows the endolysin is more potent than the antibiotic, while <100 shows the endolysin is less potent than the antibiotic.

#### 4.13.3. Turbidity Reduction and Bacterial Lysis Assay

The antibacterial activity of LysPALS22 was evaluated by monitoring the reduction in optical density (OD600) of bacterial suspensions and calculating percent lysis. Bacterial strains (*S. aureus*, *M. luteus*, *E. coli*, and *K. pneumoniae*) were grown to mid-log phase, harvested by centrifugation, washed, and finally resuspended in PBS (OD_600_ ≈ 0.5; ≈10^7^ CFU/mL). For the assay, aliquots (180–200 µL) were dispensed into 96-well plates, and purified LysPALS22 was added to final concentrations of 25, 50, 100, and 150 µg/mL. PBS was used as negative control. OD_600_ was measured at 0, 30, 60, 90, 120, and 150 min at 37 °C using a microplate reader. Bacterial lysis was expressed both as a decrease in turbidity (OD_600_) over time and as percent lysis relative to the untreated control using the standard formula:

$$\%\;\mathrm{Lysis}=\frac{\left({\mathrm{OD}}_{\mathrm{control}}-\;{\mathrm{OD}}_{\mathrm{treated}}\right)}{{\mathrm{OD}}_{\mathrm{control}}}\times 100$$

All assays were performed in biological triplicate and data were presented as the mean ± SD. Statistical analyses and graphs were generated using GraphPad Prism 10 (GraphPad Software, La Jolla, CA, USA) in accordance with standard practices.

## 5. Conclusions

This study successfully combined computational and experimental methods to characterize the LysPALS22 endolysin expressed in *E. coli* and *P. pastoris*. Structural modeling and docking analyses revealed strong binding affinity to peptidoglycan-derived ligands, while experimental expression confirmed protein production and post-translational modifications. These findings demonstrate the potential of LysPALS22 as a candidate antimicrobial agent and provide a foundation for future optimization and functional studies. Further investigations will focus on in vivo efficacy and molecular dynamics to fully elucidate its therapeutic potential. While this study does not include direct antimicrobial activity or in vivo validation, it establishes a foundational understanding of LysPALS22’s structural and binding properties and demonstrates successful expression in two heterologous systems. Future work will focus on detailed antimicrobial efficacy assays and in vivo studies to evaluate the therapeutic potential of these recombinant endolysins.

## Figures and Tables

**Table 1:** Structural alignment results between Endo_PALS22_Ec_AlphaFold_EAD (reference) and various target endolysins (EADs), showing sequence alignment scores, pruned pairs, and RMSD values for both pruned and all pairs.

Reference	Target Protein	Chain	Sequence Alignment Score	Pruned Atom Pairs	RMSD Pruned (Å)	RMSD All Pairs (Å)
Endo_PALS22_Ec_AlphaFold_EAD	Endo_LysAB-vT2_EAD	D	23.2	4	1.112	16.823
Endo_PALS22_Ec_AlphaFold_EAD	Endo_LysAm24_EAD	A	20.9	6	1.549	20.871
Endo_PALS22_Ec_AlphaFold_EAD	Endo_LysCSA13_EAD	A	31.7	4	0.677	15.02
Endo_PALS22_Ec_AlphaFold_EAD	Endo_LysECD7_EAD	A	34.6	8	1.215	11.834
Endo_PALS22_Ec_AlphaFold_EAD	Endo_LysH5_EAD1	A	14.3	4	0.731	14.053
Endo_PALS22_Ec_AlphaFold_EAD	Endo_LysH5_EAD2	A	177.7	69	0.973	4.165
Endo_PALS22_Ec_AlphaFold_EAD	Endo_LysPA26_EAD	A	24.4	23	0.965	20.343
Endo_PALS22_Ec_AlphaFold_EAD	Endo_LysPALS22_Pp_EAD	A	748.5	138	0.509	0.63
Endo_PALS22_Ec_AlphaFold_EAD	Endo_LysZX4-NCA_EAD	A	24.9	7	1.473	16.224
Endo_PALS22_Ec_AlphaFold_EAD	Endo_Ply113_EAD	C	14.6	5	0.987	12.155
Endo_PALS22_Ec_AlphaFold_EAD	Endo_T5-Zn2+_EAD	A	32.8	5	1.188	14.828
Endo_PALS22_Ec_AlphaFold_EAD	Endo_LysB4_EAD	A	17.6	7	0.983	14.942
Endo_PALS22_Ec_AlphaFold_EAD	Endo_LysP108_EAD	A	149.8	65	1.121	4.796
Endo_PALS22_Ec_AlphaFold_EAD	Endo_PlyG_EAD_EAD	A	227.5	97	1.116	2.746

**Table 2:** Antimicrobial activity with ZOI, MICs, and MBCs of the purified LysPALS22 against the Gram-positive bacteria, Gram-negative bacteria, yeast, and fungi.

Microorganism	CIP	Amp	Lys-PALS22	MIC	MBC/ MFC	AMI (CIP)	PAI (CIP)	AMI (Amp)	PAI (Amp)
	ZOI (mm)	ZOI (mm)	ZOI (mm)	(μg/mL)	(μg/mL)				
*M. luteus* ATCC 4698	20	21	22	30	30	1.1	110	1.04	104
*B. subtilis* ATCC 6051	18	20	19	44	44	1.05	105	0.95	95
*S. aureus* ATCC 25923	19	21	21	50	50	1.1	110	1.0	100
*S. epidermidis* ATCC 12228	18.5	22	19	55	55	1.03	103	0.86	86
*S. saprophyticus* ATCC 15305	19	22.5	18	63	63	0.95	95	0.80	80
*S. pneumoniae* ATCC 6303	17.5	22	17	69	69	0.97	97	0.77	77
*E. coli* ATCC 25922	17	19	16	84	84	0.94	94	0.84	84
*P. aeruginosa* ATCC 9027	16.5	18	15	101	101	0.91	91	0.83	83
*K. pneumoniae* ATCC 13883	16	17	14	107	107	0.88	88	0.82	82
*S. typhimurium* ATCC 14028	14	15	13	111	111	0.93	93	0.86	86
*S. cerevisiae*	18	19	15	140	140	0.83	83	0.78	78
*A. oryzae*	15	16	14	105	105	0.93	93	0.87	87

Note: Control/antibiotic for bacteria was ciprofloxacin and ampicillin, and for yeast and fungi was miconazole.

**Table 3:** List of endolysins analyzed in this study, including their corresponding protein names, accession IDs, and bacteriophage origins. (*GB: GenBank).

Protein Name	GB* Accession	Bacteriophage Origin
Endo_LysAB-vT2	QHJ75684.1	*Acinetobacter phage vB_AbaM_PhT2*
Endo_LysAm24	APD20282.1	*Acinetobacter phage AM24*
Endo_LysB4	AFF27501.1	*Bacillus phage B4*
Endo_LysCSA13	AWD93110.1	*Staphylococcus phage CSA13*
Endo_LysECD7	ASJ80195.1	*Escherichia phage ECD7*
Endo_LysH5	ACE77796.1	*Staphylococcus phage phiH5*
Endo_LysP108	AIK69635.1	*Staphylococcus phage P108*
Endo_LysPA26	ARB16052.1	*Bacteriophage sp. isolate*
Endo_LysZX4-NCA	QTH79953.1	*Klebsiella phage vB_KpnS_ZX4*
Endo_Ply113	QVW54600.1	*Enterococcus phage 113*
Endo_PlyG_CBD	PDB: 2L48_A	*Bacillus phage Gamma*
Endo_PlyG_EAD	PDB: 2L47_A	*Bacillus phage Gamma*
Endo_T5-Zn2^+^	AAS19387.1 (PDB: 2MXZ)	*Enterobacteria phage T5*
Endo_T5-Zn2^+^/Ca2^+^	AAX11973.1 (PDB: 8P3A)	*Enterobacteria phage T5*

**Table 4:** Resistance/susceptibility profile of microorganisms.

Microorganism	ATCC No.	Resistance/Susceptibility Pattern
*Micrococcus luteus*	4698	Antibiotic-susceptible reference strain
*Bacillus subtilis*	6051	Antibiotic-susceptible reference strain
*Staphylococcus aureus*	25923	CLSI quality control strain; antibiotic-susceptible
*Staphylococcus epidermidis*	12228	Antibiotic-susceptible laboratory strain
*Staphylococcus saprophyticus*	15305	Antibiotic-susceptible reference strain
*Streptococcus pneumoniae*	6303	Antibiotic-susceptible reference strain
*Escherichia coli*	25922	CLSI quality control strain; antibiotic-susceptible
*Pseudomonas aeruginosa*	9027	Non-multidrug-resistant reference strain
*Klebsiella pneumoniae*	13883	Antibiotic-susceptible reference strain
*Salmonella Typhimurium*	14028	Antibiotic-susceptible reference strain
*Saccharomyces cerevisiae*	–	Laboratory yeast strain; not antibiotic-resistant
*Aspergillus oryzae*	–	Laboratory mold strain; not antibiotic-resistant

**Table 5:** Primers sequences with restriction sites (underlined) of both recombinant plasmids pET28a–*LysPALS22* and pPICZαA–*LysPALS22*.

Primer	Sequences	Restriction Enzymes	Plasmids
Sense-Eco	CATGCCATGGATGCATCATCATCATCATCACGCGAAAAAAC	NcoI	pET28a—*LysPALS22*
Antisense-Eco	GGCCTCGAGTTAAGAGAAGGTACCCCA	XhoI	
Primer1-F	CCGGAATTCGCTAAGAAGCATATTGG	EcoRI	pPICZαA—*LysPALS22*
Primer1-R	ATTTGCGGCCGCTTAATGATGATGATGATGATGGGAGAAAGTACCCC	NotI	

## Data Availability

The original contributions presented in this study are included in the article/Supplementary Material. Further inquiries can be directed to the corresponding authors.

## Supplementary Materials

The following supporting information can be downloaded at: https://www.mdpi.com/article/10.3390/ijms26178579/s1.

## Abbreviations

The following abbreviations are used in this manuscript:

EAD	Enzymatically Active Domain
CBD	Cell Wall Binding Domain
CAP	Contig Assembly Program
ORF	Open Reading Frame
PDB	Protein Data Base
GRAVY	grand average of hydropathicity
MSA	multiple sequence alignment
NJ	Neighbor-Joining
VADAR	Volume, Area, Dihedral Angle Reporter
RMSD	Root Mean Square Deviation
SDS-PAGE	Sodium Dodecyl Sulfate Polyacrylamide Gel Electrophoresis
IPTG	Isopropyl β-D-1-thiogalactopyranoside
BCA	Bicinchoninic acid
