# Supplementary material for: Integrative In Silico and Experimental Characterization of Endolysin LysPALS22: Structural Diversity, Ligand Binding Affinity, and Heterologous Expression
Source: Int J Mol Sci. 2025 Sep 3;26(17):8579. doi: 10.3390/ijms26178579 (PMC12429052; doi:10.3390/ijms26178579)
Supplement: Supplementary file 1 [file ijms-26-08579-s001.zip › ijms-3815293-supplementary files/ijms-3815293-supplementary files.pptx]

## Slide 1
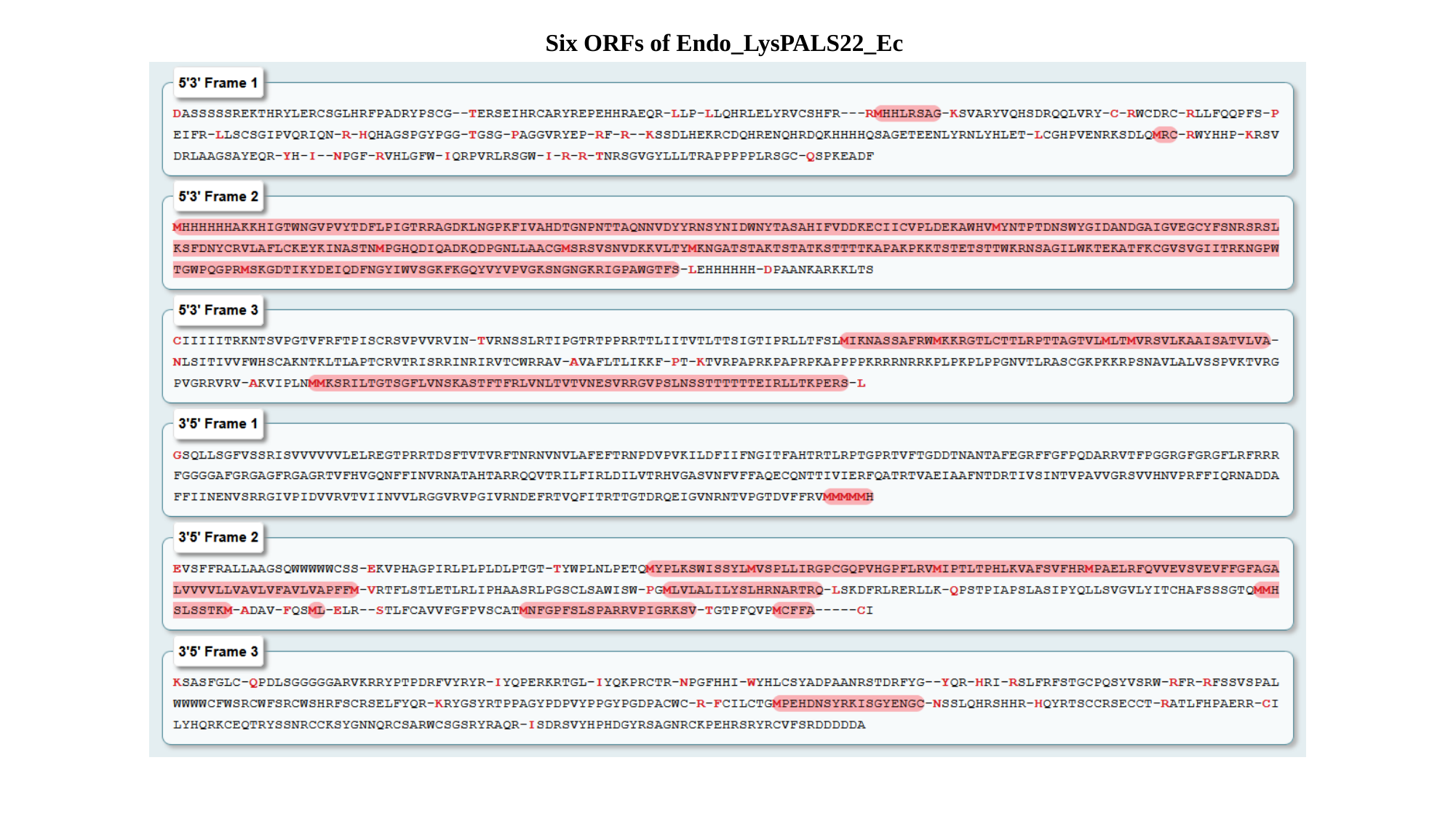

Six ORFs of Endo_LysPALS22_Ec

## Slide 2
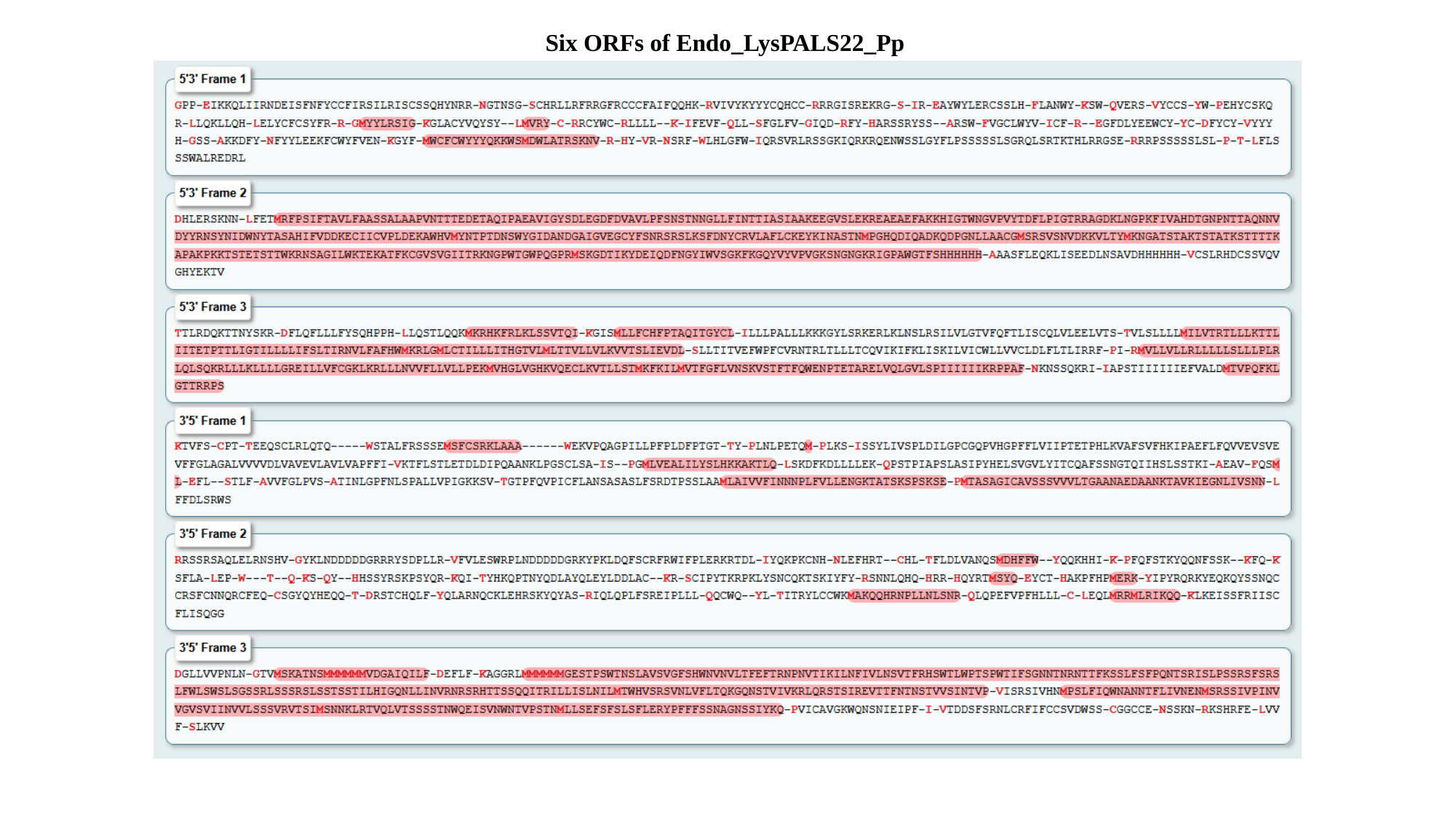

Six ORFs of Endo_LysPALS22_Pp

## Slide 3
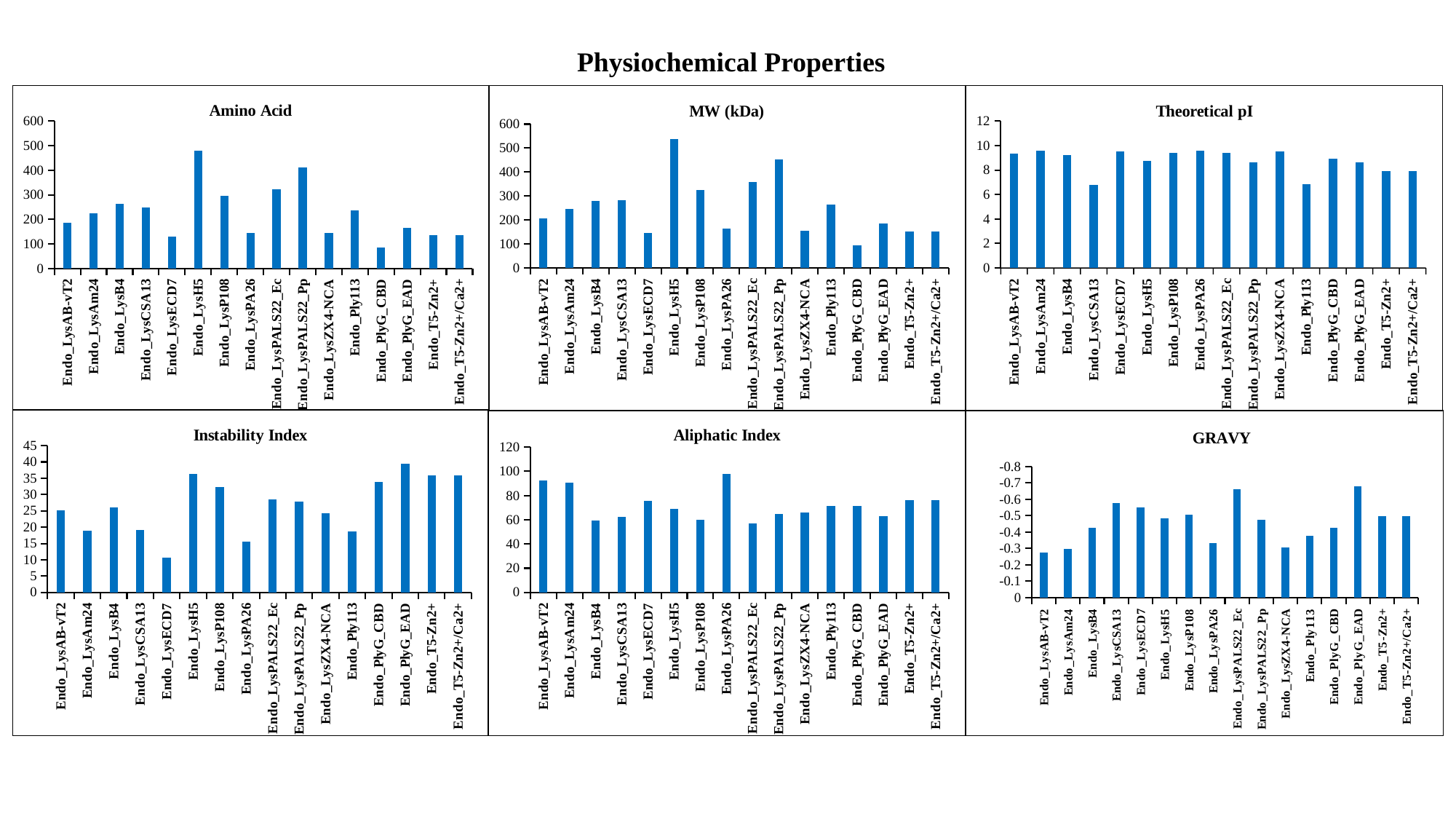

Physiochemical Properties
### Chart:
| Category | MW (kDa) |
|---|---|
| Endo_LysAB-vT2 | 207.8489 |
| Endo_LysAm24 | 245.6716 |
| Endo_LysB4 | 278.8297 |
| Endo_LysCSA13 | 283.6258 |
| Endo_LysECD7 | 147.41989999999998 |
| Endo_LysH5 | 537.6555999999999 |
| Endo_LysP108 | 324.135 |
| Endo_LysPA26 | 163.37879999999998 |
| Endo_LysPALS22_Ec | 357.8423 |
| Endo_LysPALS22_Pp | 452.5072 |
| Endo_LysZX4-NCA | 156.30870000000002 |
| Endo_Ply113 | 263.1782 |
| Endo_PlyG_CBD | 94.3776 |
| Endo_PlyG_EAD | 185.3087 |
| Endo_T5-Zn2+ | 152.6423 |
| Endo_T5-Zn2+/Ca2+ | 152.6423 |
### Chart:
| Category | Theoretical pI |
|---|---|
| Endo_LysAB-vT2 | 9.32 |
| Endo_LysAm24 | 9.61 |
| Endo_LysB4 | 9.21 |
| Endo_LysCSA13 | 6.81 |
| Endo_LysECD7 | 9.52 |
| Endo_LysH5 | 8.74 |
| Endo_LysP108 | 9.38 |
| Endo_LysPA26 | 9.6 |
| Endo_LysPALS22_Ec | 9.4 |
| Endo_LysPALS22_Pp | 8.63 |
| Endo_LysZX4-NCA | 9.54 |
| Endo_Ply113 | 6.82 |
| Endo_PlyG_CBD | 8.94 |
| Endo_PlyG_EAD | 8.6 |
| Endo_T5-Zn2+ | 7.91 |
| Endo_T5-Zn2+/Ca2+ | 7.91 |
### Chart:
| Category | Amino Acid |
|---|---|
| Endo_LysAB-vT2 | 187.0 |
| Endo_LysAm24 | 224.0 |
| Endo_LysB4 | 262.0 |
| Endo_LysCSA13 | 249.0 |
| Endo_LysECD7 | 129.0 |
| Endo_LysH5 | 481.0 |
| Endo_LysP108 | 295.0 |
| Endo_LysPA26 | 145.0 |
| Endo_LysPALS22_Ec | 322.0 |
| Endo_LysPALS22_Pp | 412.0 |
| Endo_LysZX4-NCA | 145.0 |
| Endo_Ply113 | 237.0 |
| Endo_PlyG_CBD | 85.0 |
| Endo_PlyG_EAD | 165.0 |
| Endo_T5-Zn2+ | 137.0 |
| Endo_T5-Zn2+/Ca2+ | 137.0 |
### Chart:
| Category | Instability Index |
|---|---|
| Endo_LysAB-vT2 | 25.12 |
| Endo_LysAm24 | 18.87 |
| Endo_LysB4 | 26.01 |
| Endo_LysCSA13 | 19.09 |
| Endo_LysECD7 | 10.63 |
| Endo_LysH5 | 36.37 |
| Endo_LysP108 | 32.29 |
| Endo_LysPA26 | 15.66 |
| Endo_LysPALS22_Ec | 28.57 |
| Endo_LysPALS22_Pp | 27.9 |
| Endo_LysZX4-NCA | 24.18 |
| Endo_Ply113 | 18.61 |
| Endo_PlyG_CBD | 33.81 |
| Endo_PlyG_EAD | 39.49 |
| Endo_T5-Zn2+ | 35.78 |
| Endo_T5-Zn2+/Ca2+ | 35.78 |
### Chart:
| Category | Aliphatic Index |
|---|---|
| Endo_LysAB-vT2 | 92.25 |
| Endo_LysAm24 | 90.49 |
| Endo_LysB4 | 59.58 |
| Endo_LysCSA13 | 62.57 |
| Endo_LysECD7 | 75.43 |
| Endo_LysH5 | 68.75 |
| Endo_LysP108 | 59.86 |
| Endo_LysPA26 | 98.07 |
| Endo_LysPALS22_Ec | 56.96 |
| Endo_LysPALS22_Pp | 64.68 |
| Endo_LysZX4-NCA | 66.0 |
| Endo_Ply113 | 71.56 |
| Endo_PlyG_CBD | 71.18 |
| Endo_PlyG_EAD | 63.21 |
| Endo_T5-Zn2+ | 76.28 |
| Endo_T5-Zn2+/Ca2+ | 76.28 |
### Chart:
| Category | GRAVY |
|---|---|
| Endo_LysAB-vT2 | -0.276 |
| Endo_LysAm24 | -0.298 |
| Endo_LysB4 | -0.427 |
| Endo_LysCSA13 | -0.576 |
| Endo_LysECD7 | -0.552 |
| Endo_LysH5 | -0.483 |
| Endo_LysP108 | -0.506 |
| Endo_LysPA26 | -0.332 |
| Endo_LysPALS22_Ec | -0.663 |
| Endo_LysPALS22_Pp | -0.476 |
| Endo_LysZX4-NCA | -0.308 |
| Endo_Ply113 | -0.378 |
| Endo_PlyG_CBD | -0.428 |
| Endo_PlyG_EAD | -0.678 |
| Endo_T5-Zn2+ | -0.495 |
| Endo_T5-Zn2+/Ca2+ | -0.495 |

## Slide 4
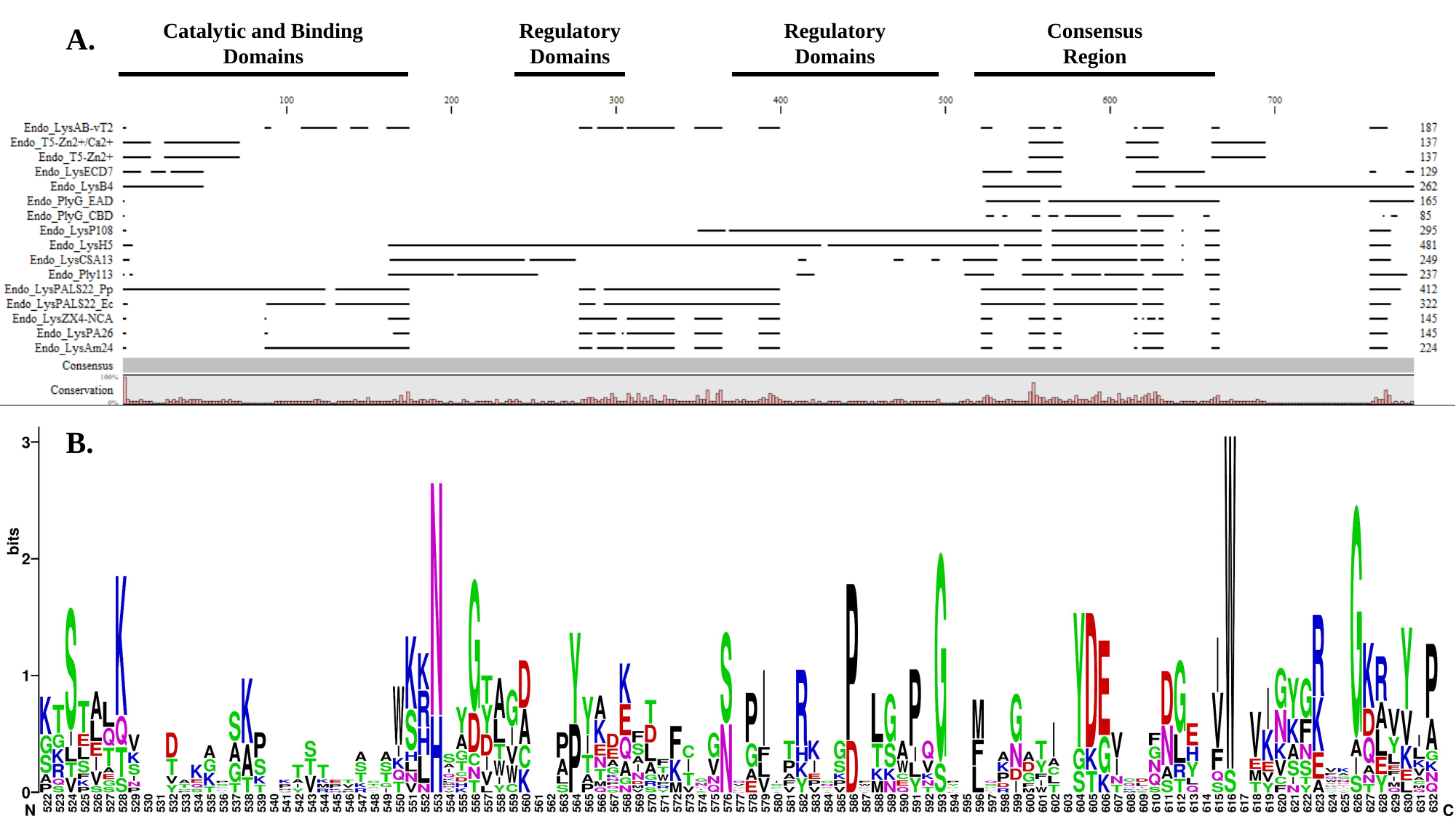

PlyG-BD
PGBD
Hh/DD-Pep
CysProt
CHAP
PGRP
Amidase family
SH3
SH3
Consensus Region
Regulatory Domains
Regulatory Domains
Catalytic and Binding Domains
A.
B.

## Slide 5
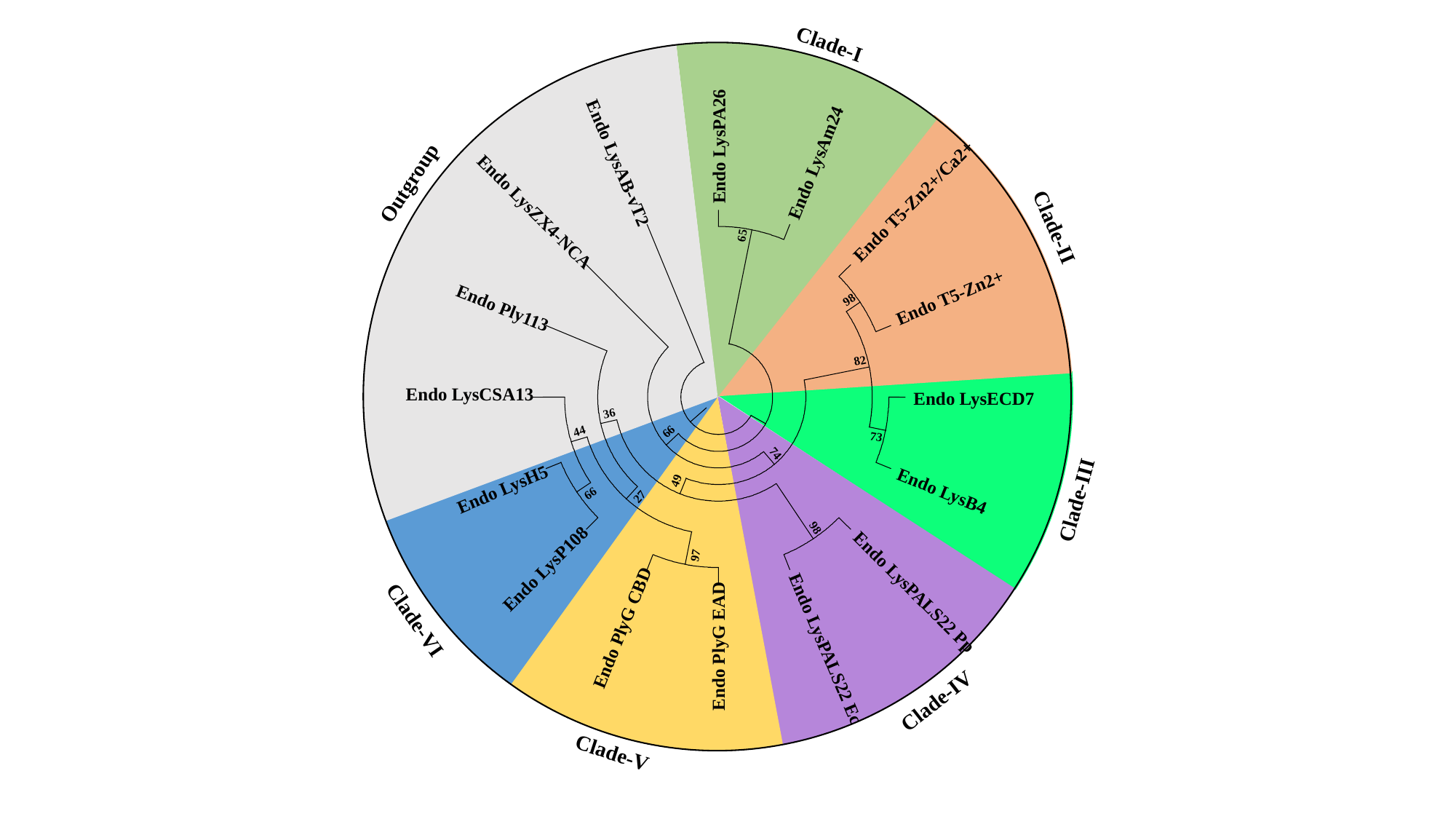

Clade-I
Endo LysPA26
Endo LysAm24
Endo LysAB-vT2
Endo T5-Zn2+/Ca2+
Endo LysZX4-NCA
65
Endo T5-Zn2+
98
Endo Ply113
82
Endo LysCSA13
Endo LysECD7
36
44
66
73
74
49
Endo LysH5
Endo LysB4
66
27
98
97
Endo LysP108
Endo LysPALS22 Pp
Endo PlyG CBD
Endo PlyG EAD
Endo LysPALS22 Ec
Outgroup
Clade-II
Clade-III
Clade-VI
Clade-IV
Clade-V

## Slide 6
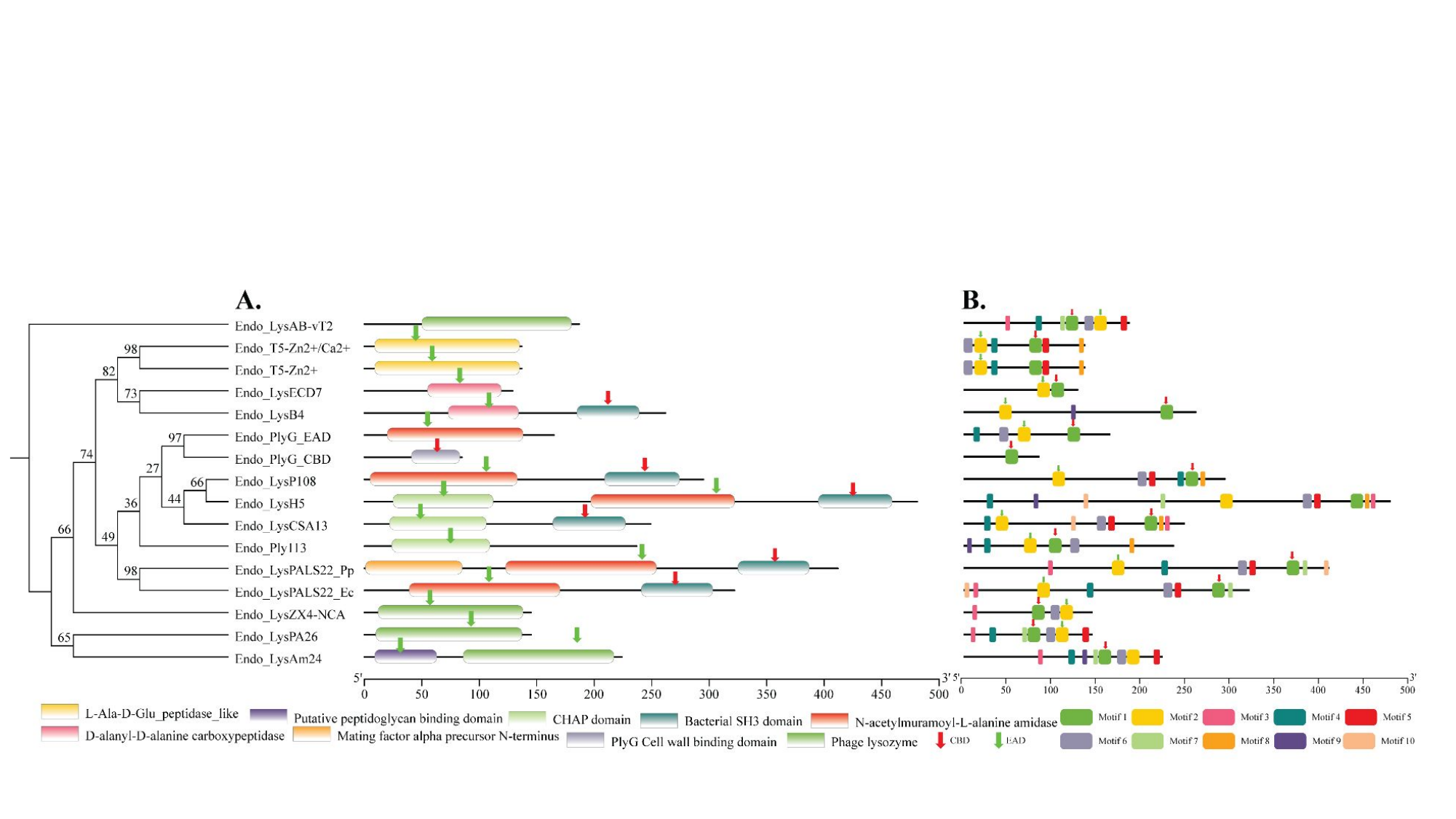

## Slide 7
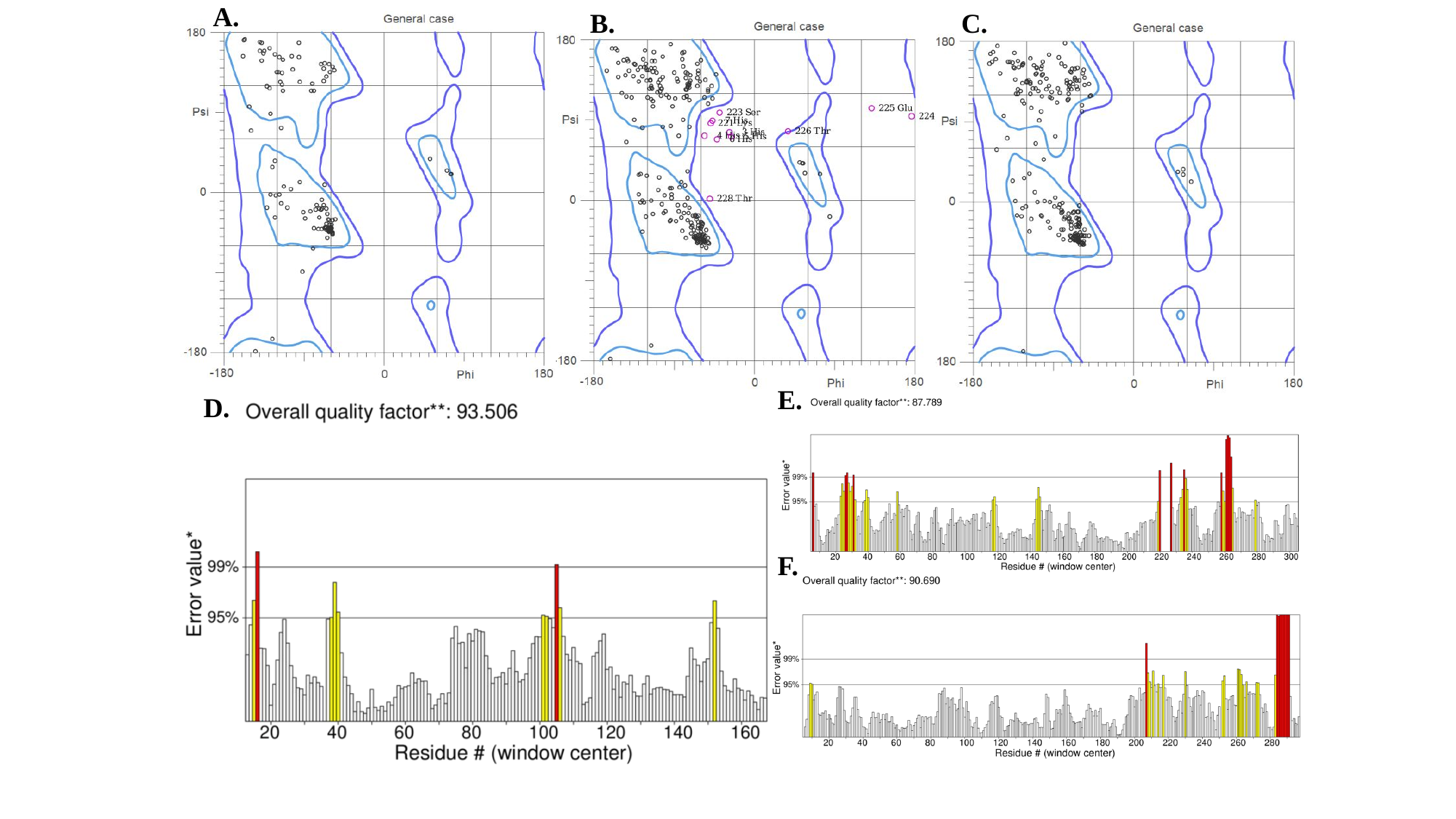

A.
B.
C.
E.
D.
F.

## Slide 8
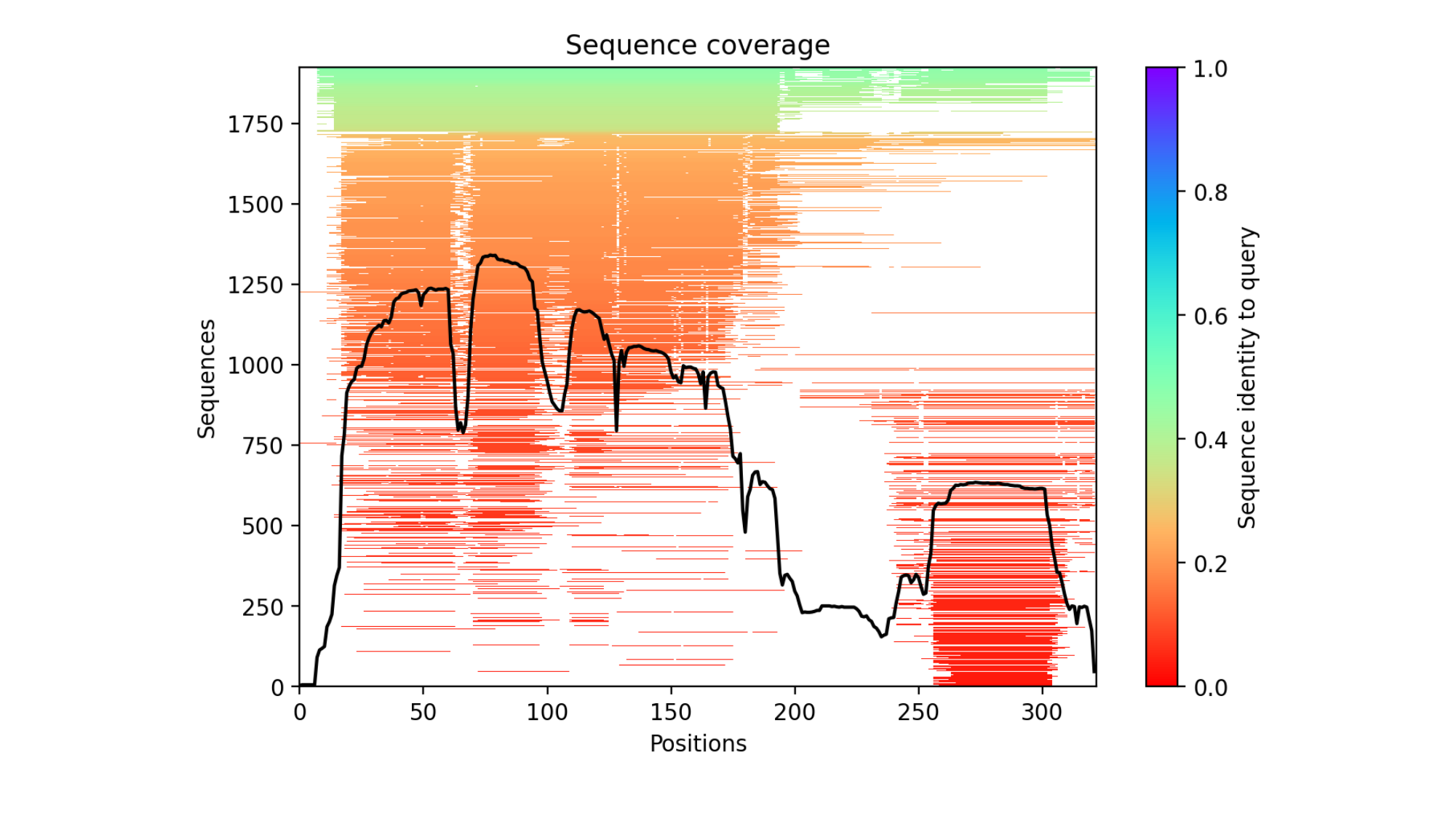

## Slide 9
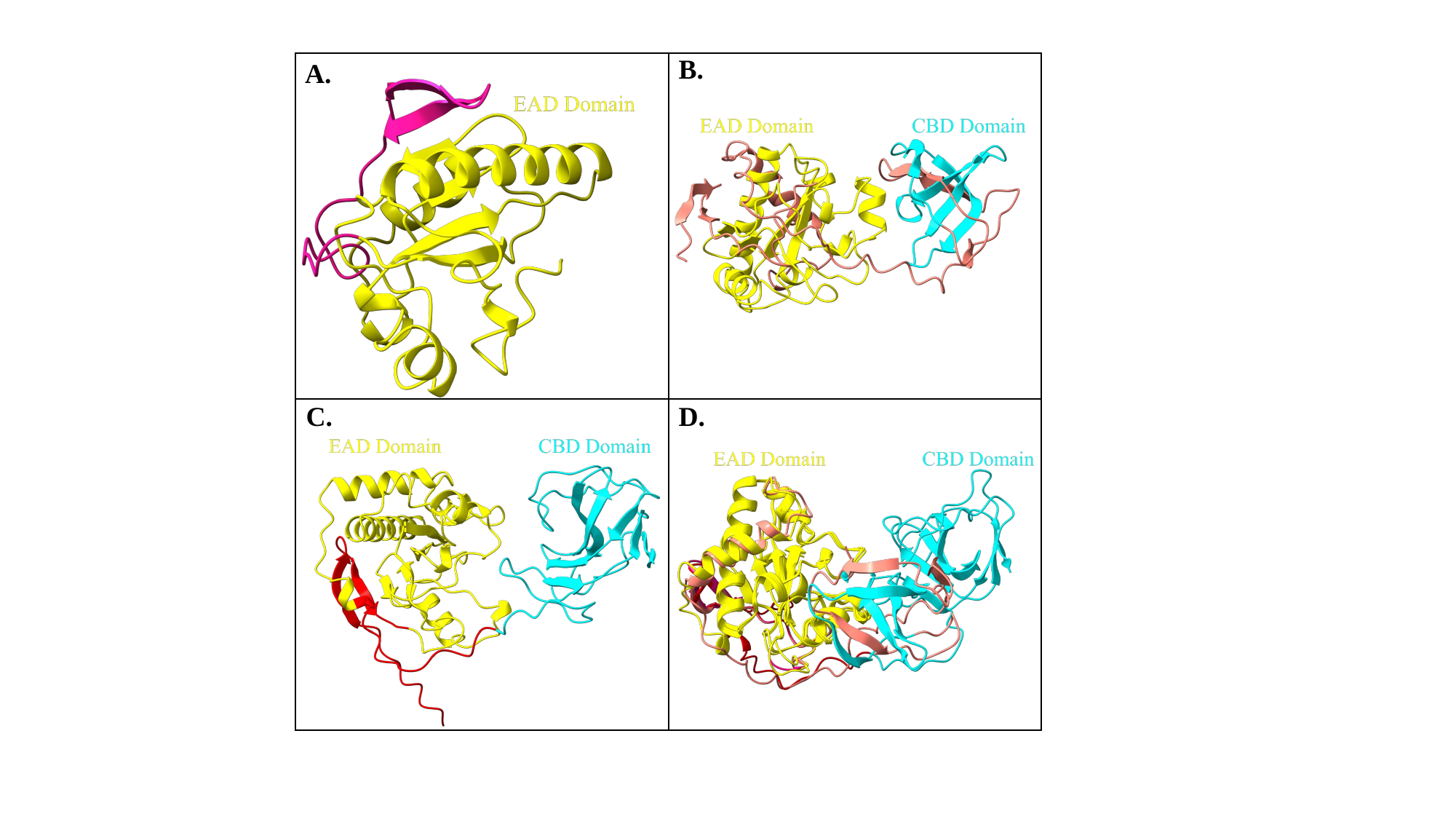

B.
A.
C.
D.

## Slide 10
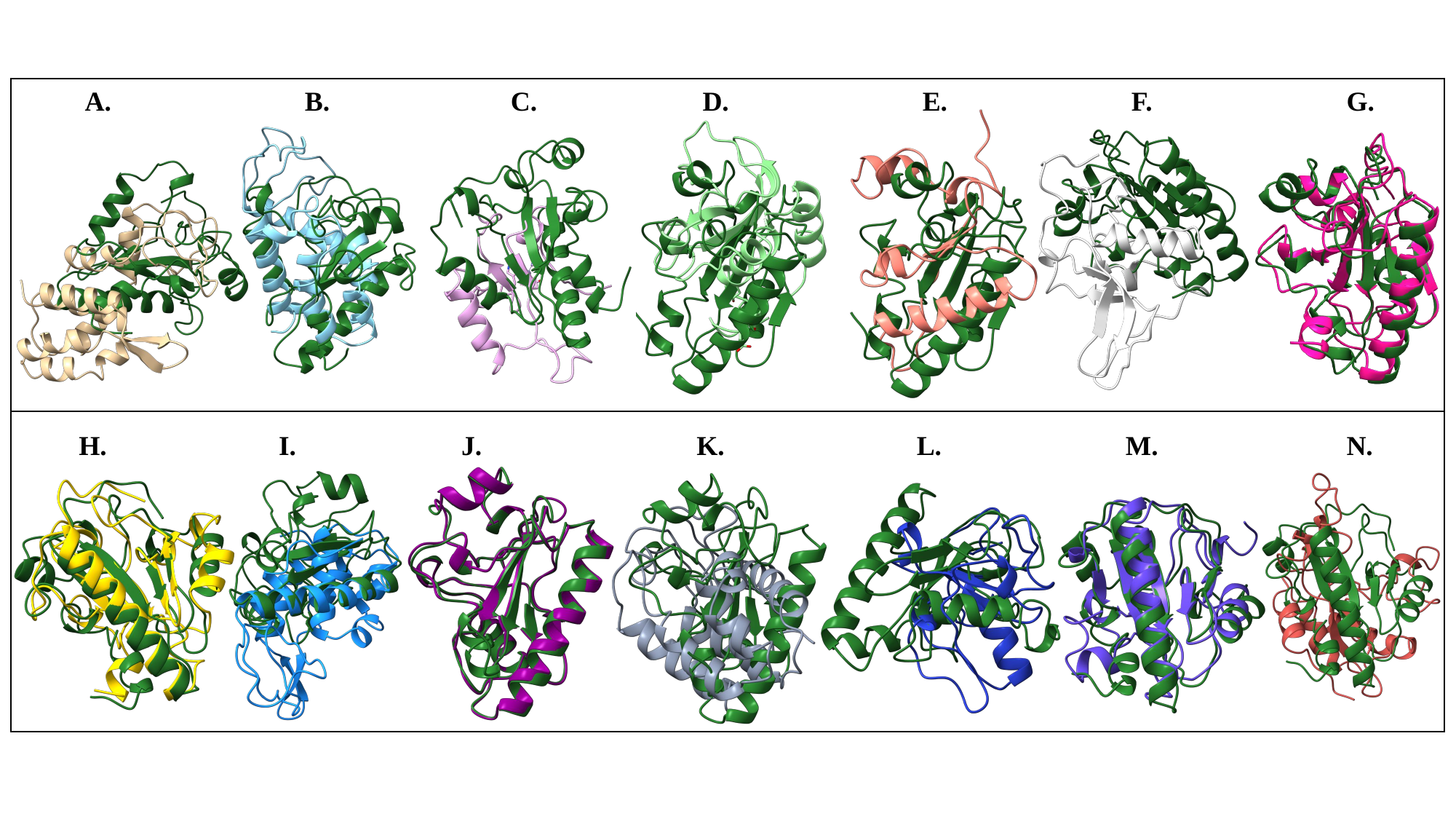

A.
B.
C.
D.
E.
F.
G.
H.
I.
J.
K.
L.
M.
N.

## Slide 11
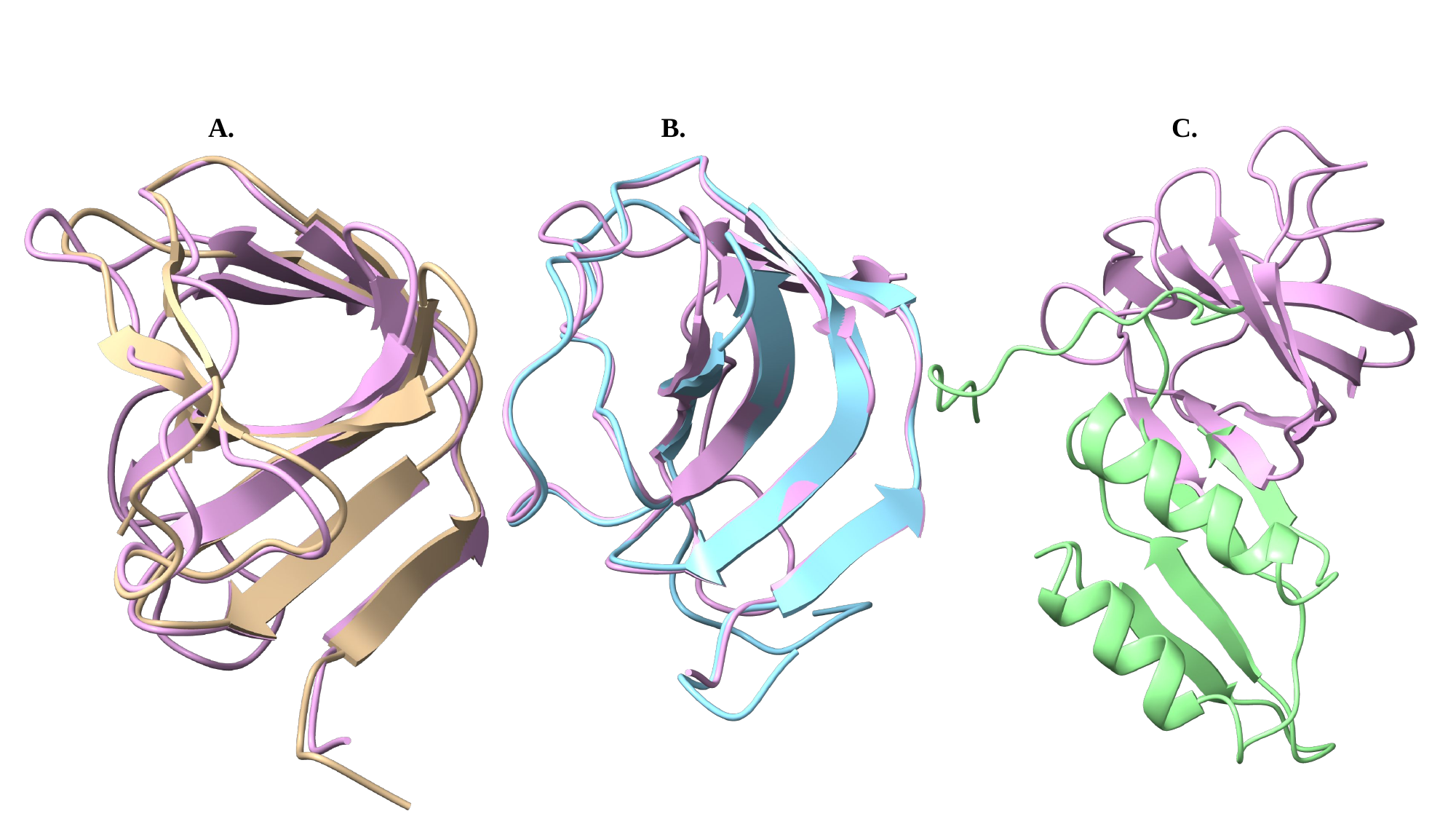

A.
B.
C.

## Slide 12
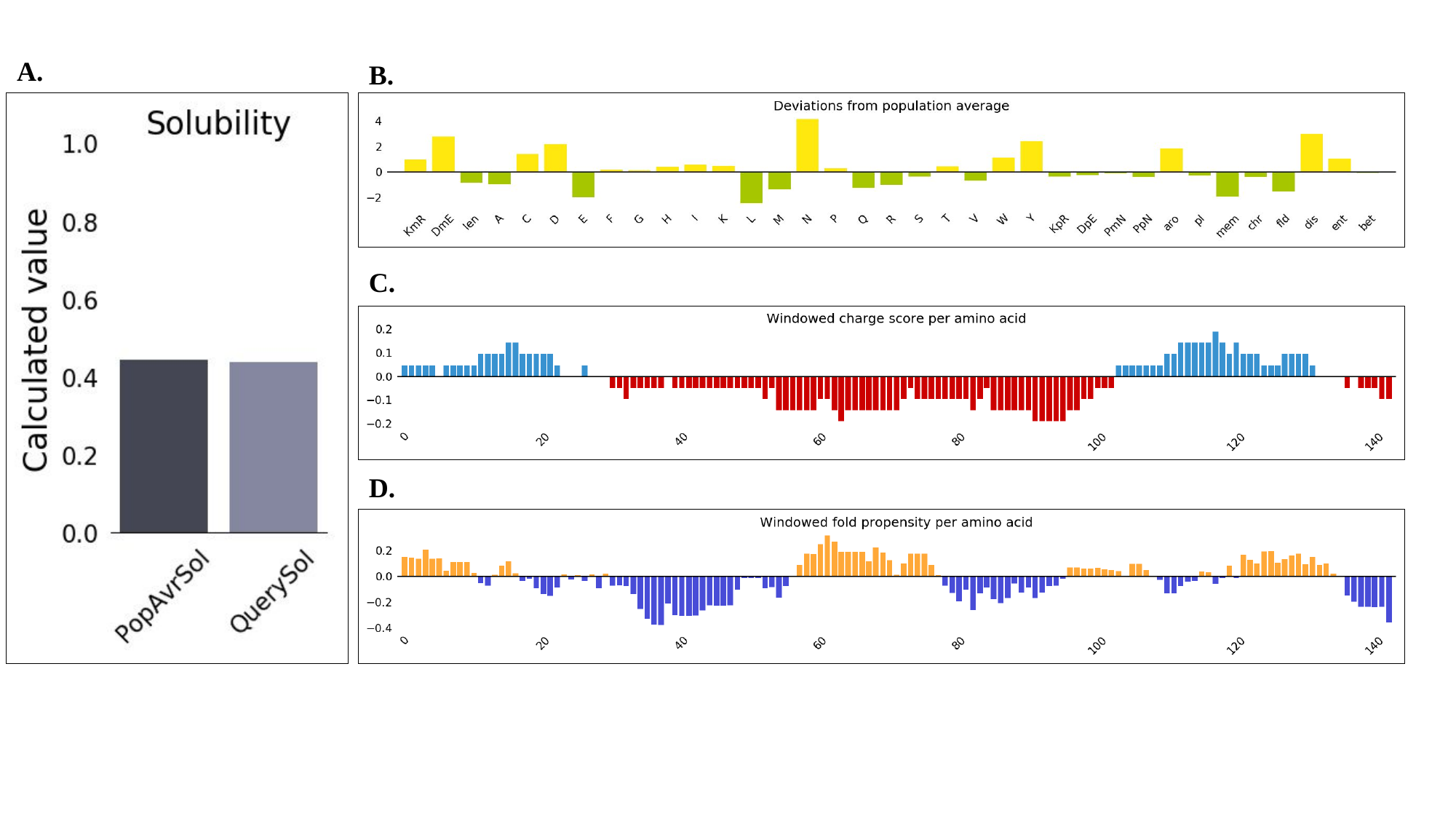

A.
B.
C.
D.

## Slide 13
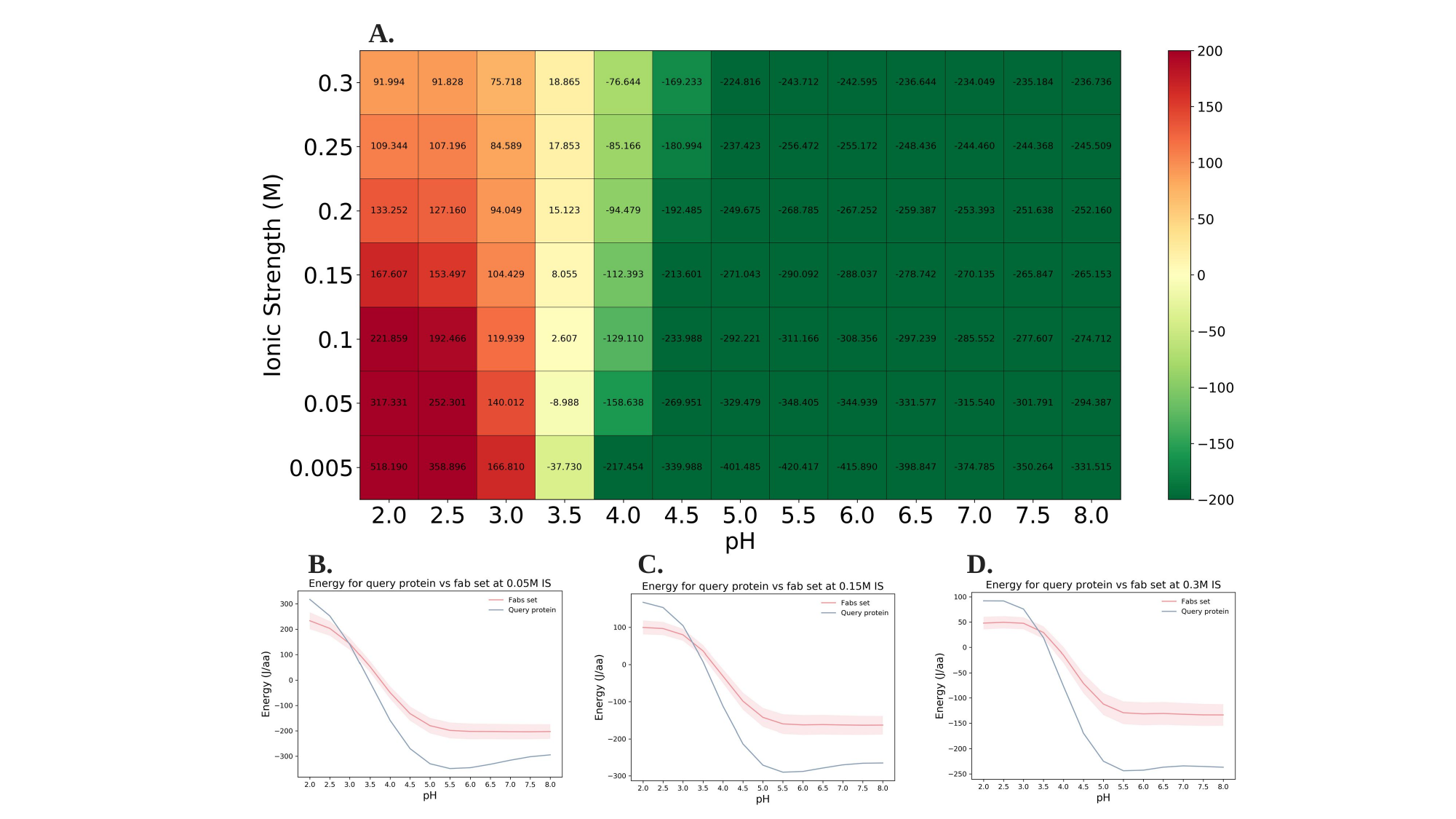

ENERGY HEATMAP
(J per aa)
A.
B.
C.
D.

## Slide 14
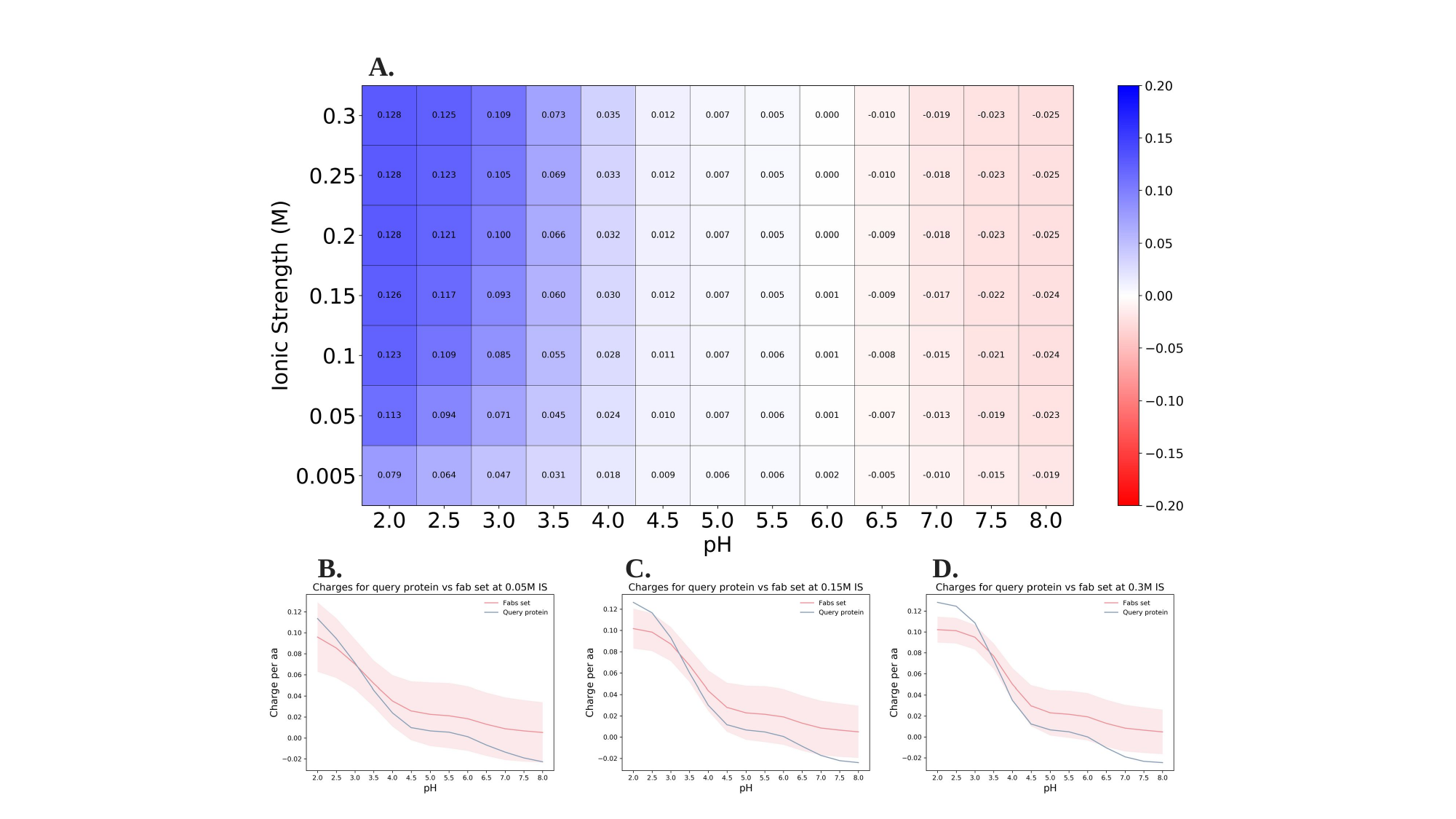

CHARGE HEATMAP
(e per aa)
A.
B.
C.
D.

## Slide 15
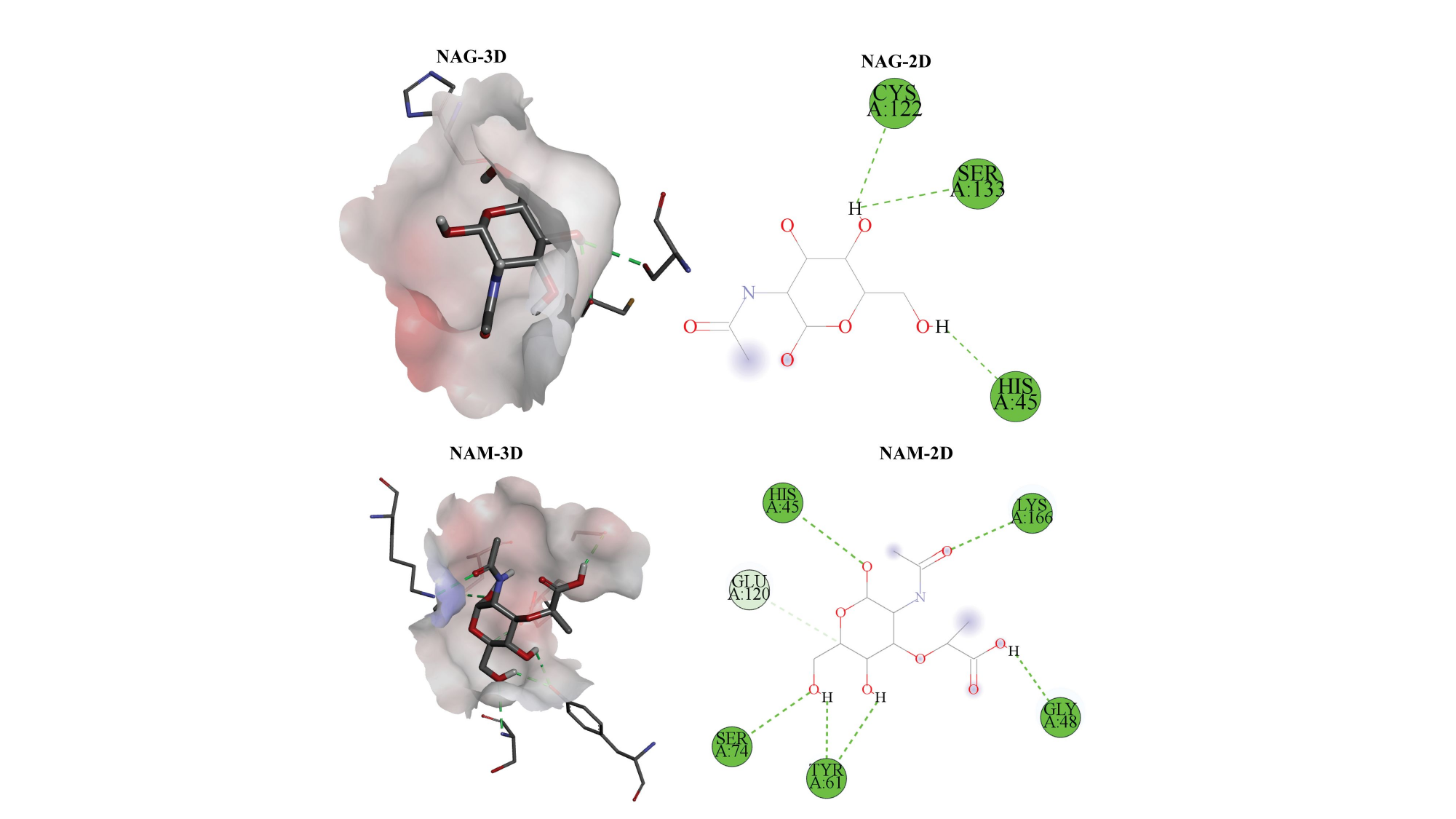

## Slide 16
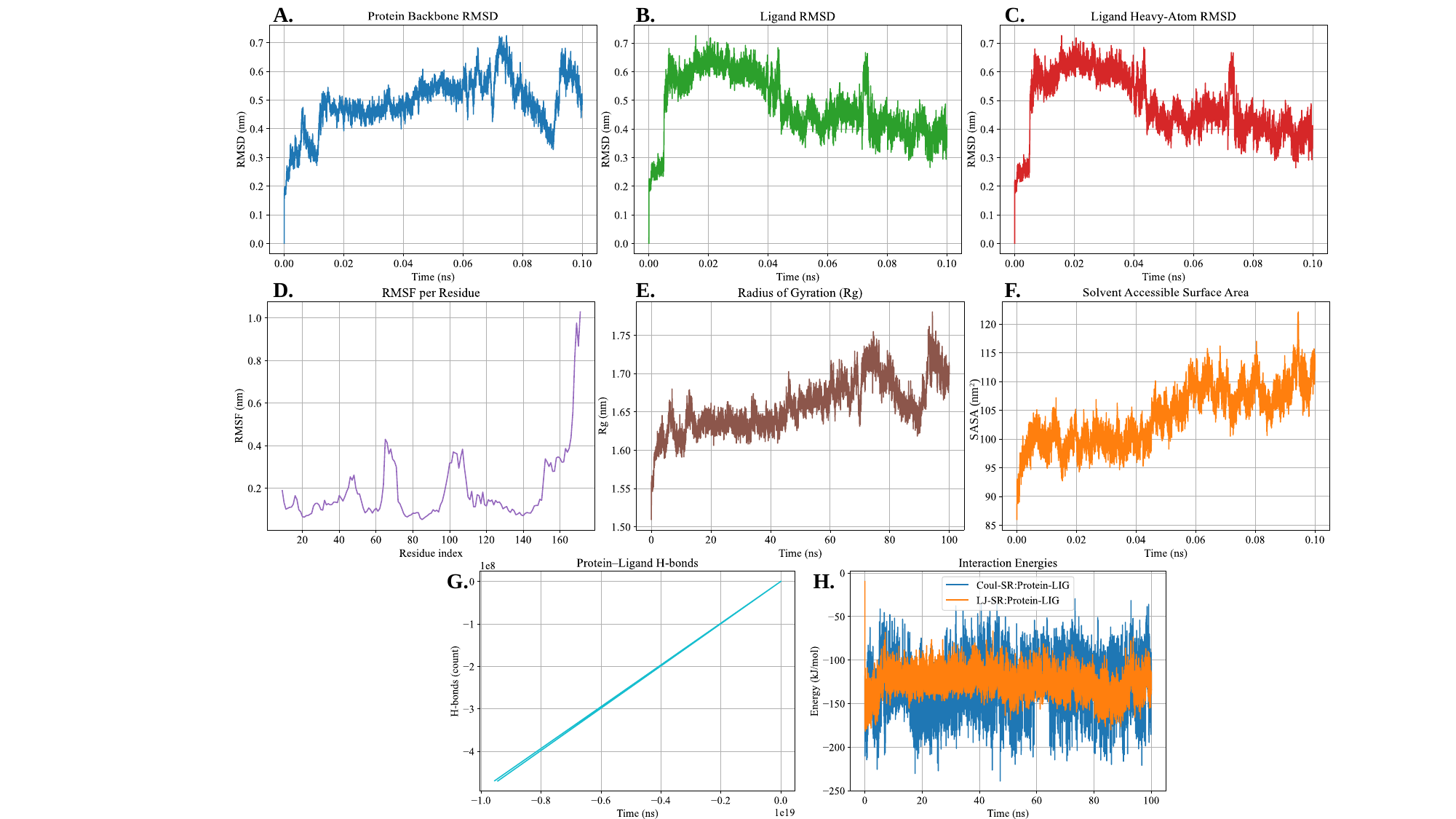

A.
B.
C.
D.
E.
F.
SASA (nm2)
G.
H.

## Slide 17
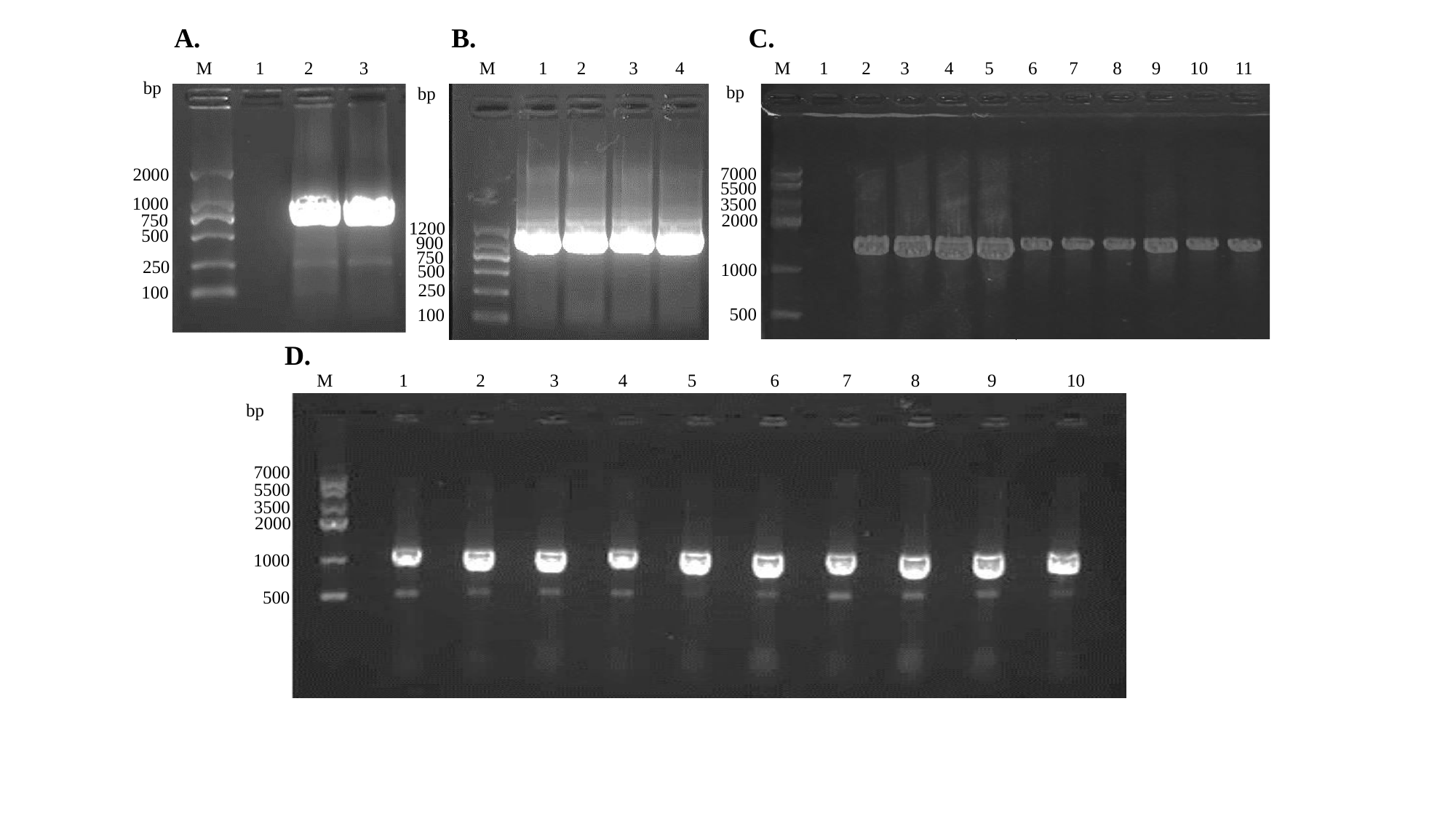

A.
B.
C.
4
3
M
1
2
bp
1200
900
750
500
250
100
11
M
1
2
3
4
5
6
7
8
9
10
bp
7000
5500
3500
2000
1000
500
M
1
2
3
bp
2000
1000
750
500
250
100
D.
M
1
2
3
4
5
6
7
8
9
10
bp
7000
5500
3500
2000
1000
500

## Slide 18
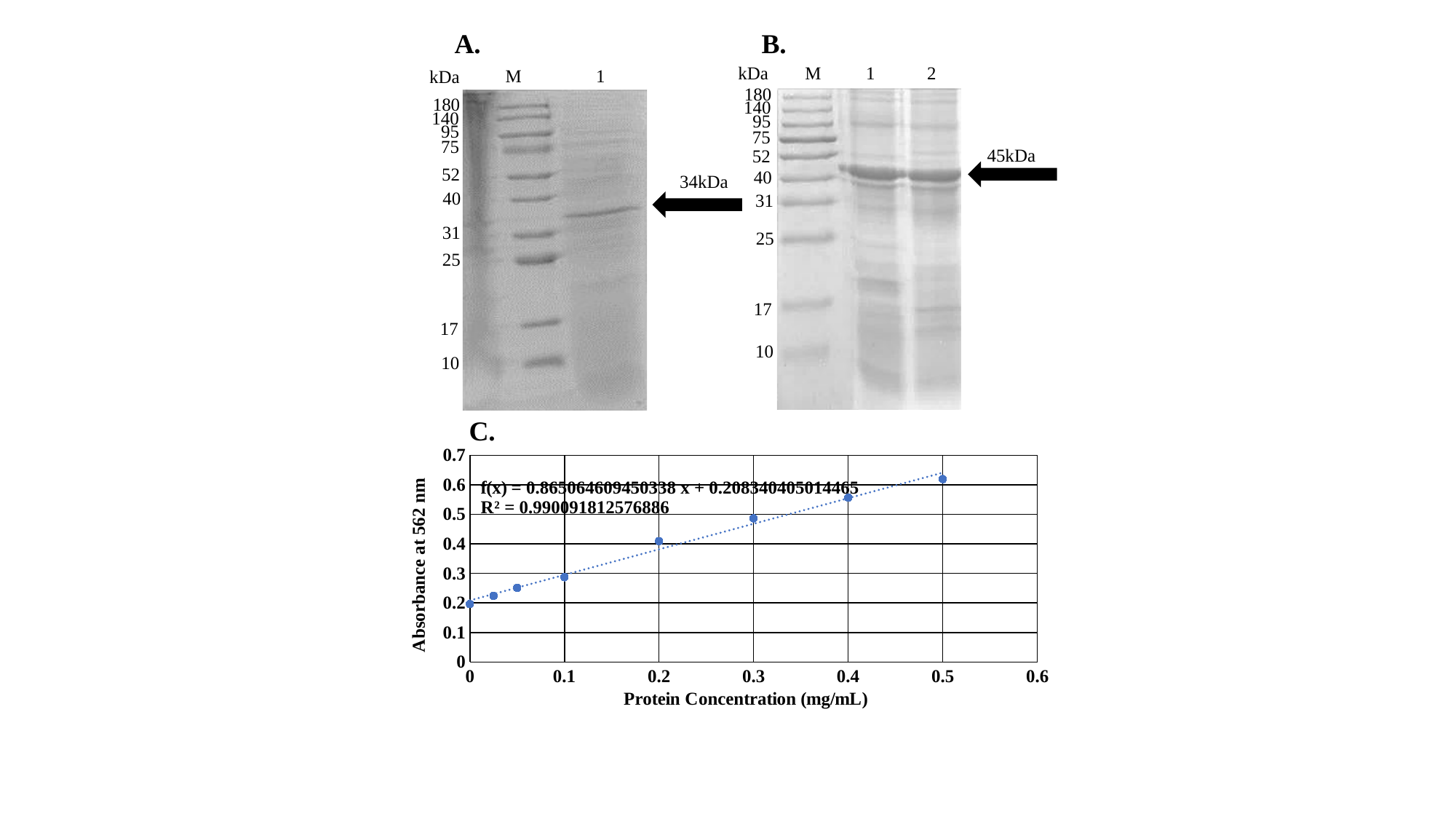

A.
B.
kDa
M
1
2
180
140
95
75
45kDa
52
40
31
25
17
10
M
1
kDa
180
140
95
75
34kDa
52
40
31
25
17
10
### Chart
| Category | OD reading |
|---|---|C.

## Slide 19
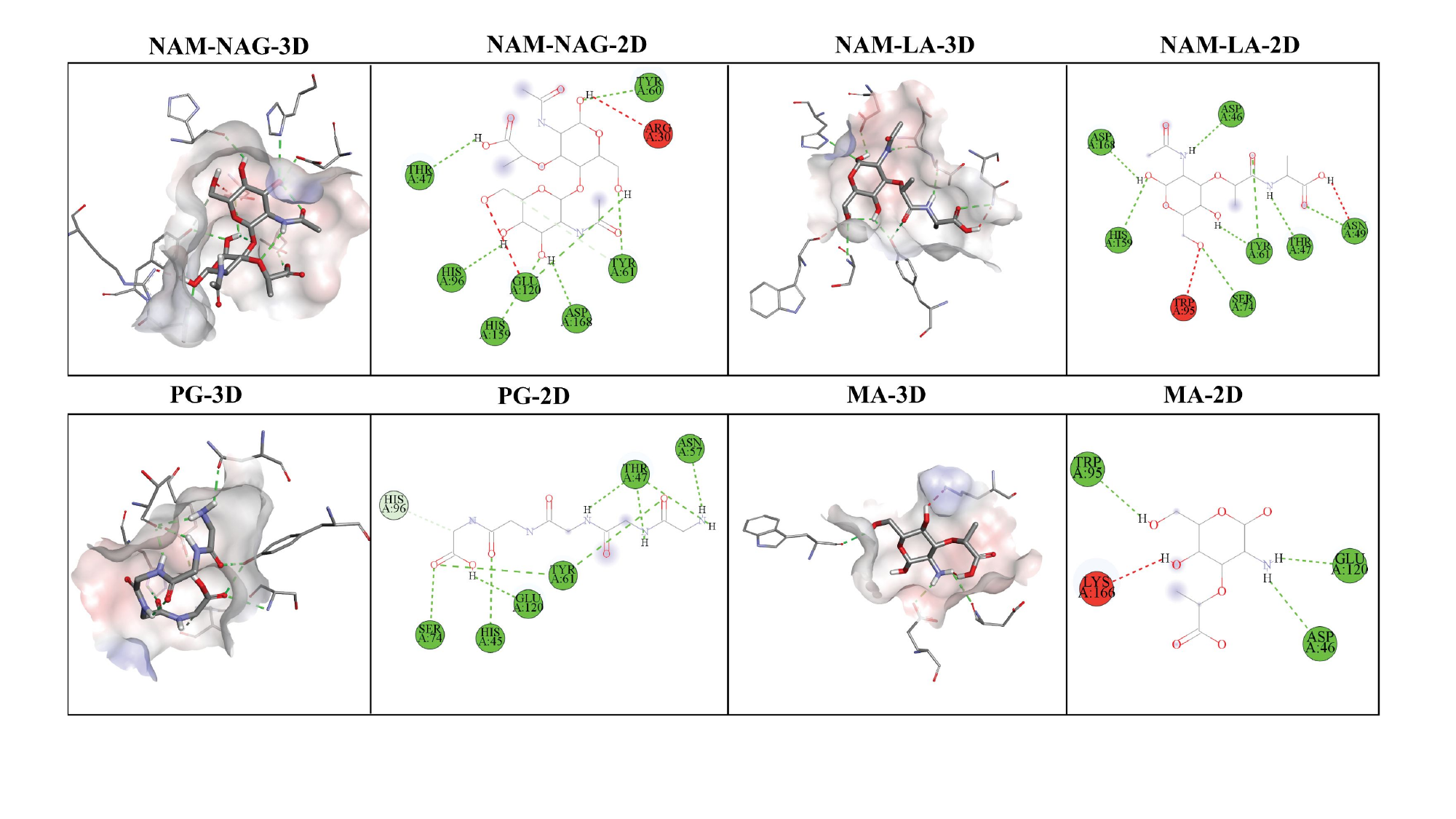

## Slide 20
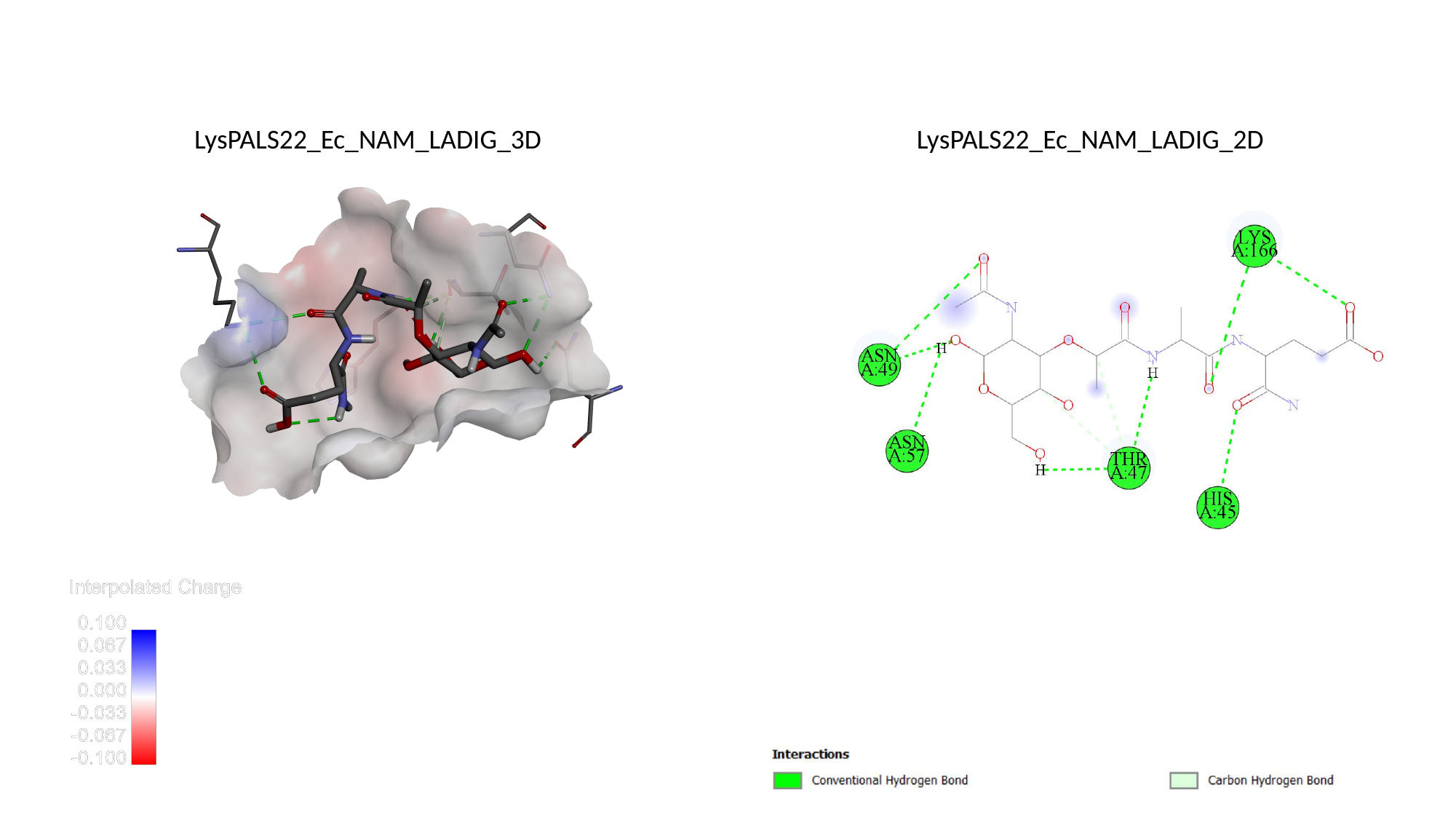

LysPALS22_Ec_NAM_LADIG_3D
LysPALS22_Ec_NAM_LADIG_2D

## Slide 21
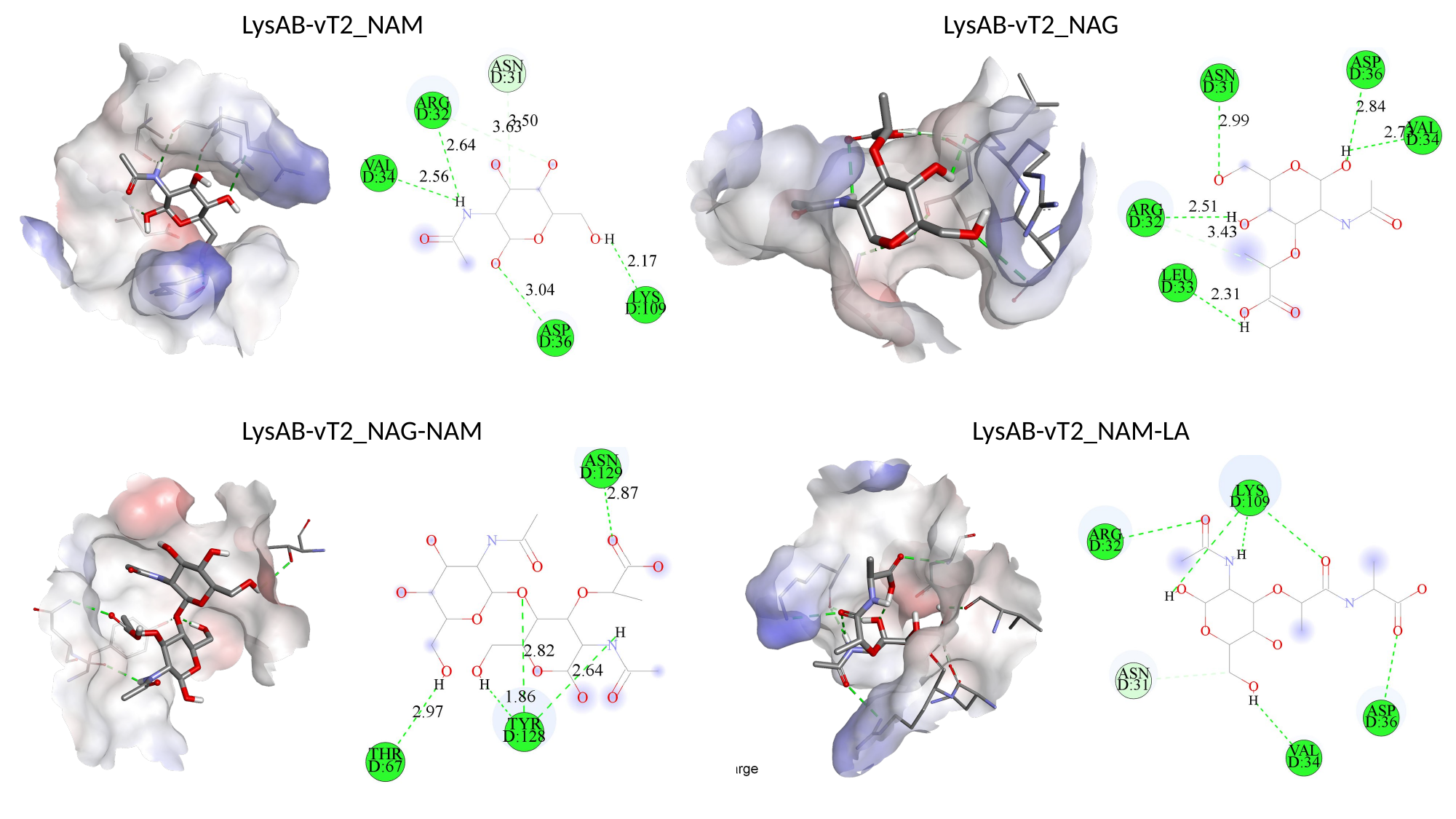

LysAB-vT2_NAM
LysAB-vT2_NAG
LysAB-vT2_NAG-NAM
LysAB-vT2_NAM-LA

## Slide 22
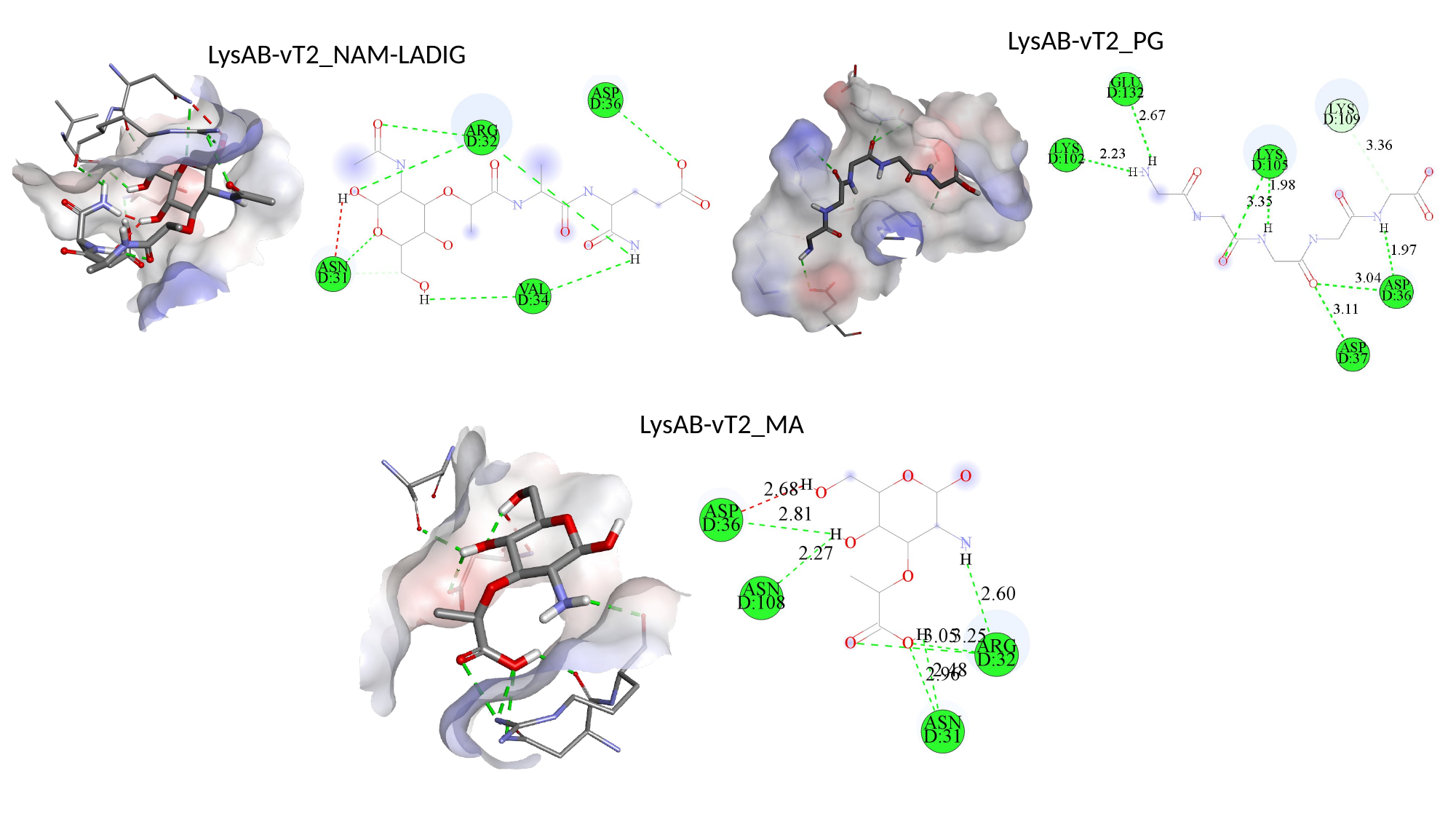

LysAB-vT2_PG
LysAB-vT2_NAM-LADIG
LysAB-vT2_‏MA

## Slide 23
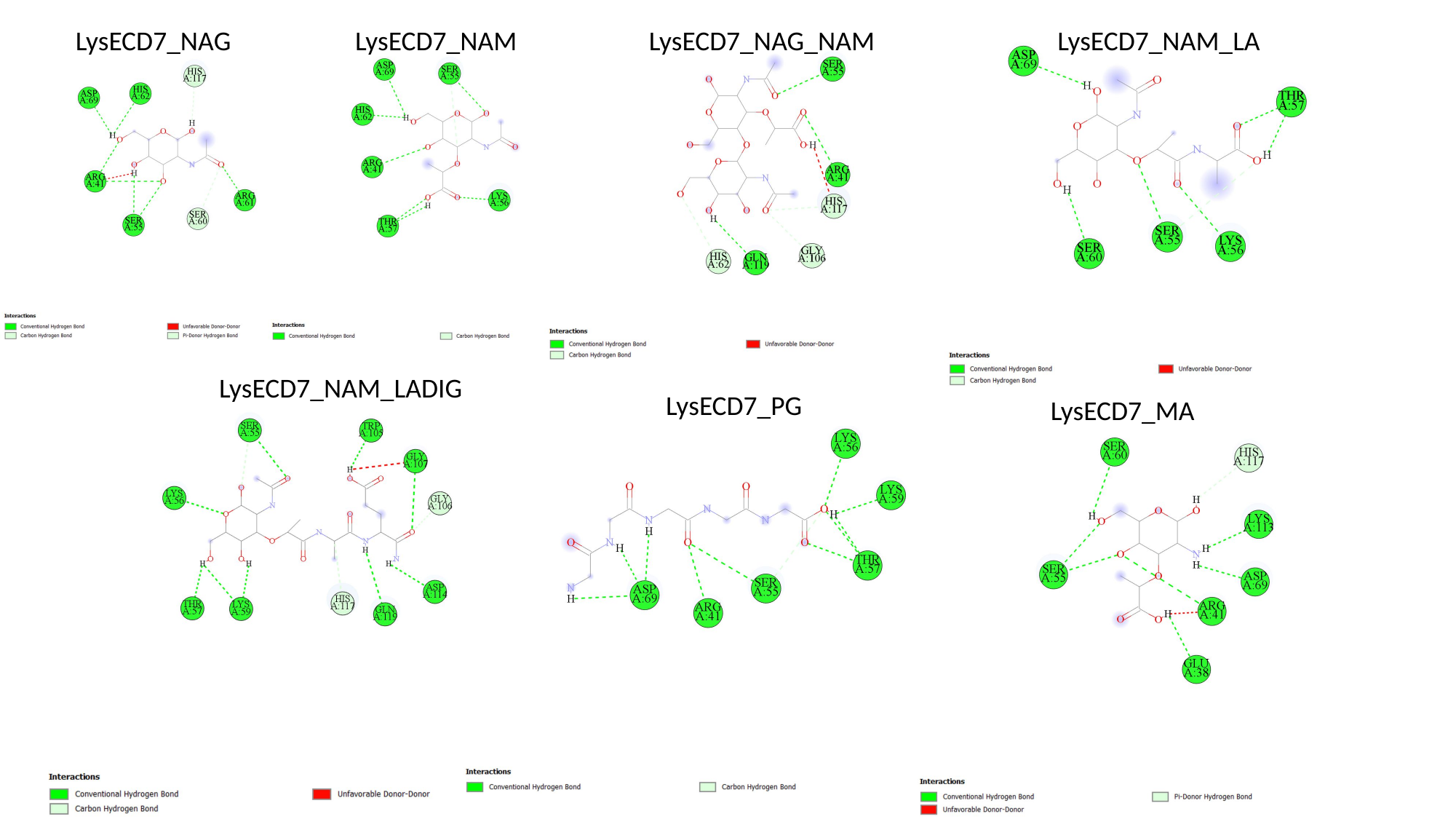

LysECD7_NAG
LysECD7_NAM
LysECD7_NAG_NAM
LysECD7_NAM_LA
LysECD7_NAM_LADIG
LysECD7_PG
LysECD7_MA

## Slide 24
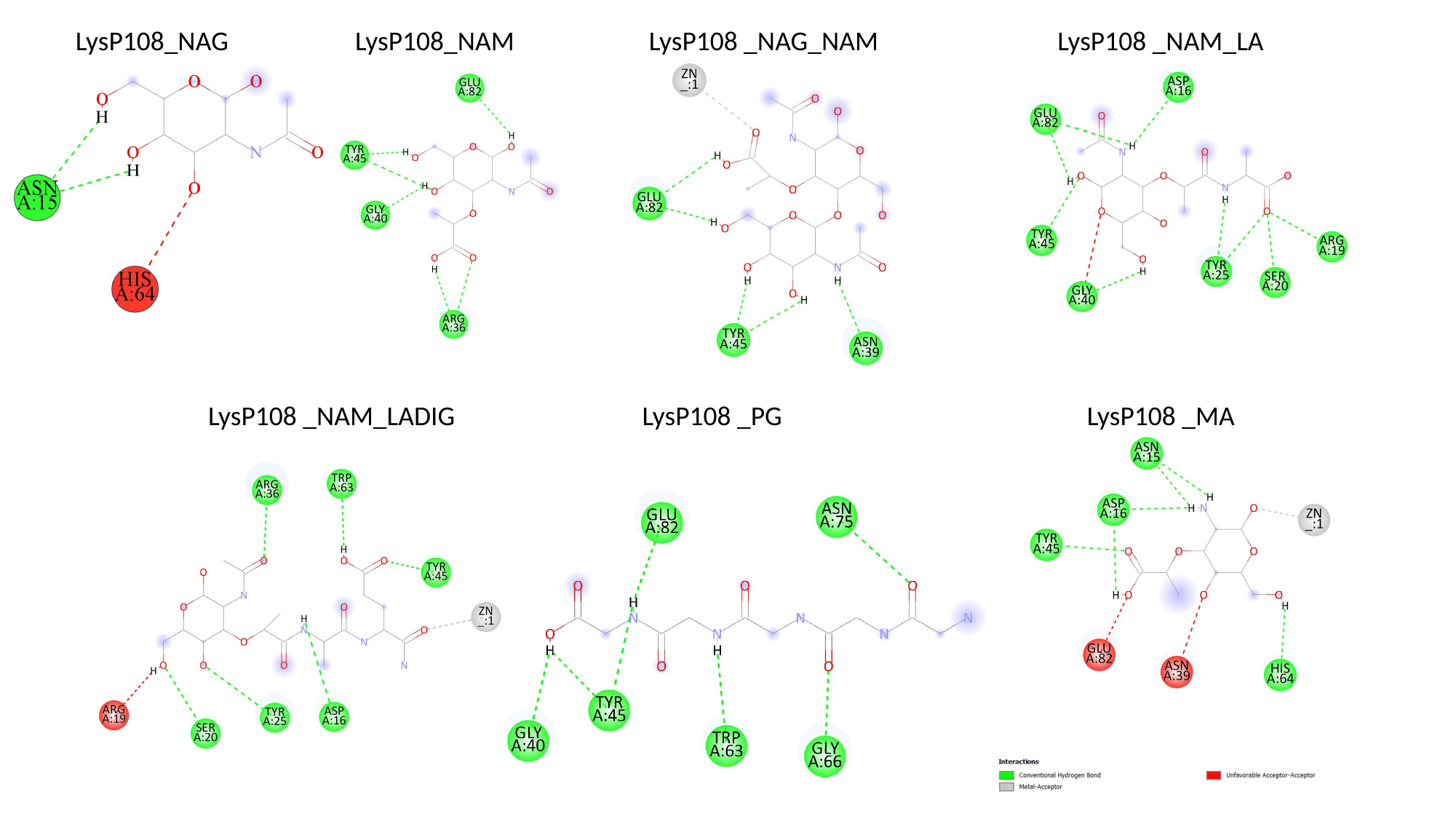

LysP108_NAG
LysP108_NAM
LysP108 _NAG_NAM
LysP108 _NAM_LA
LysP108 _NAM_LADIG
LysP108 _PG
LysP108 _MA

## Slide 25
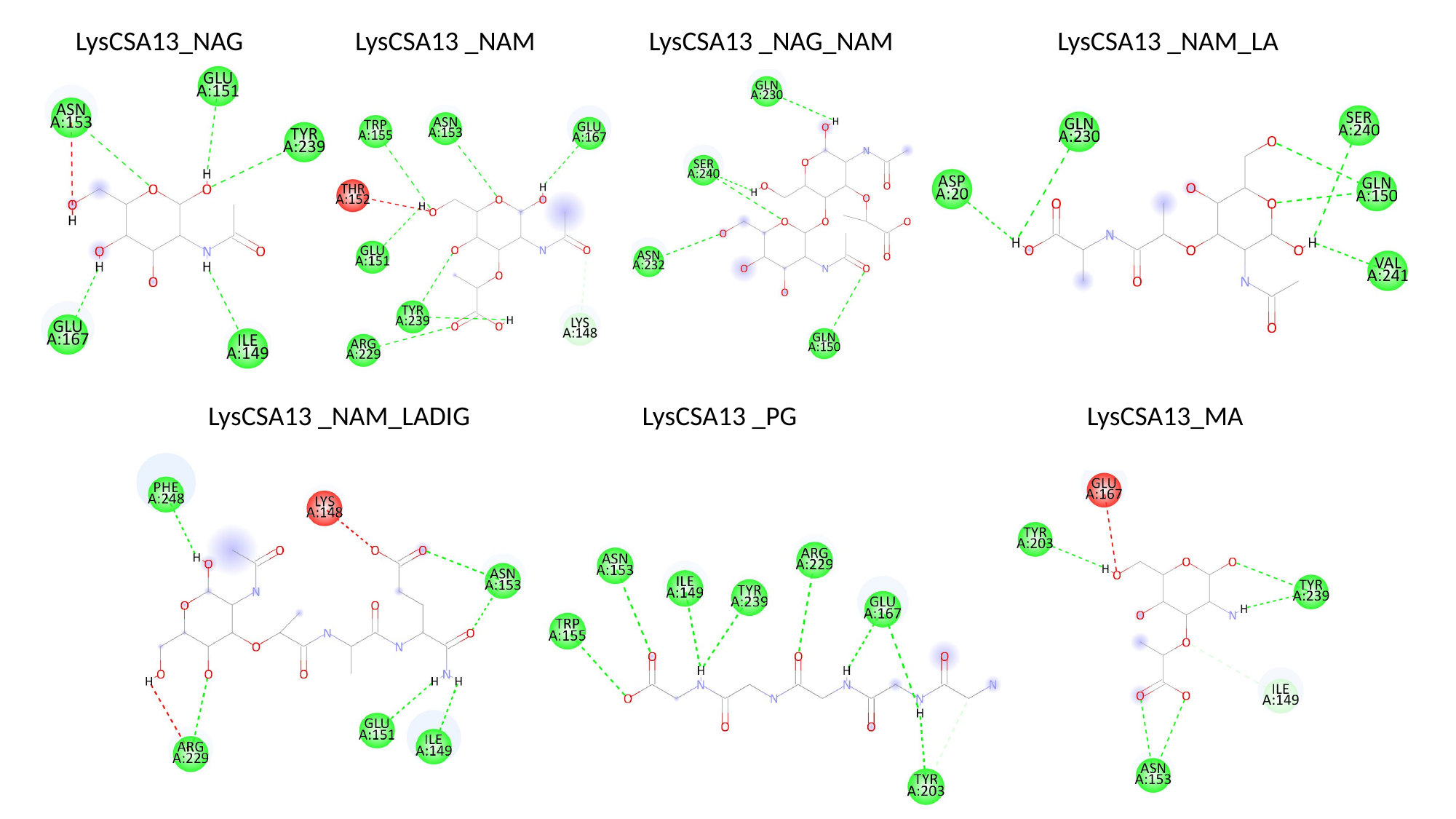

LysCSA13_NAG
LysCSA13 _NAM
LysCSA13 _NAG_NAM
LysCSA13 _NAM_LA
LysCSA13 _NAM_LADIG
LysCSA13 _PG
LysCSA13_MA

## Slide 26
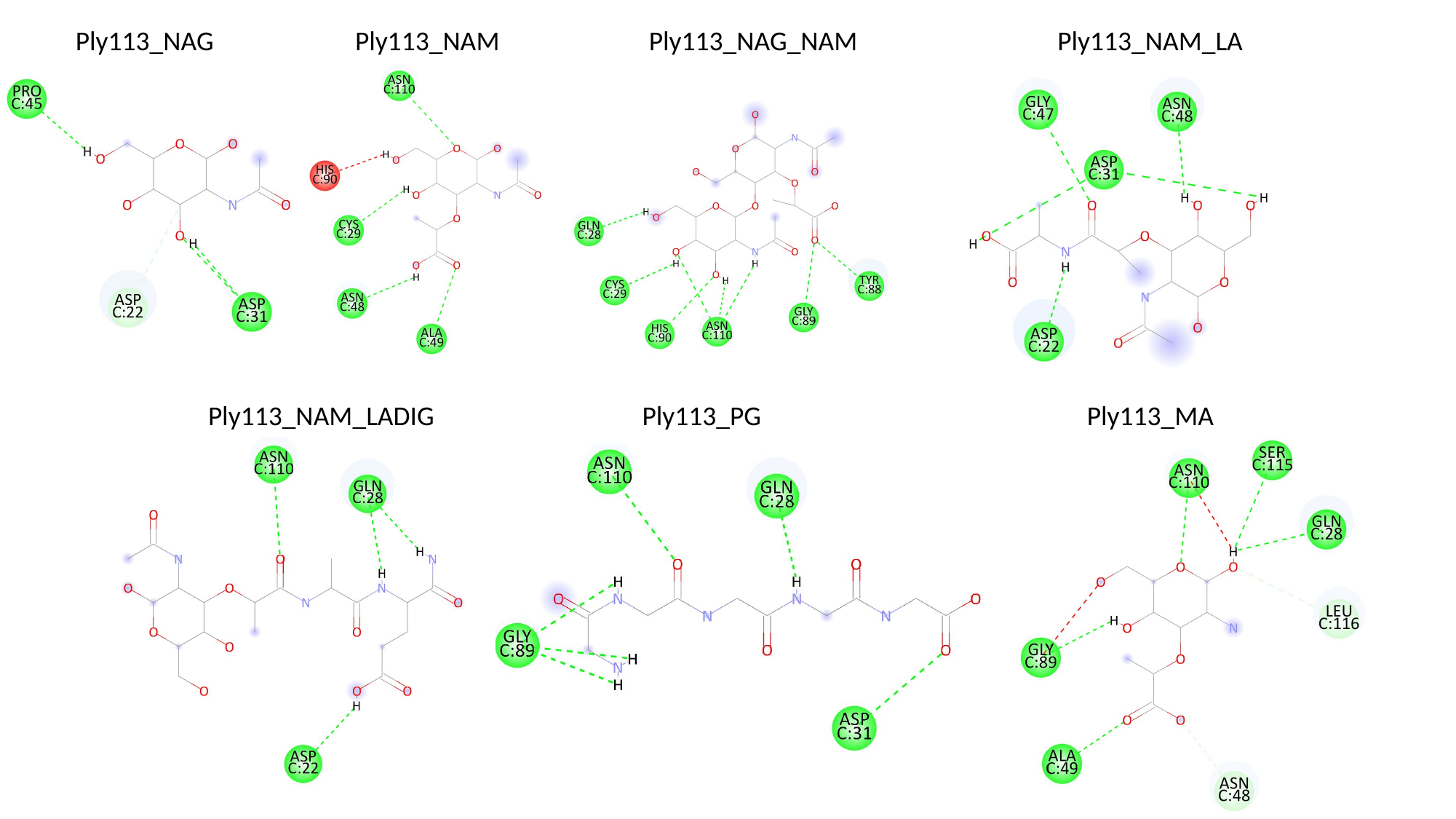

Ply113_NAG
Ply113_NAM
Ply113_NAG_NAM
Ply113_NAM_LA
Ply113_NAM_LADIG
Ply113_PG
Ply113_MA

## Slide 27
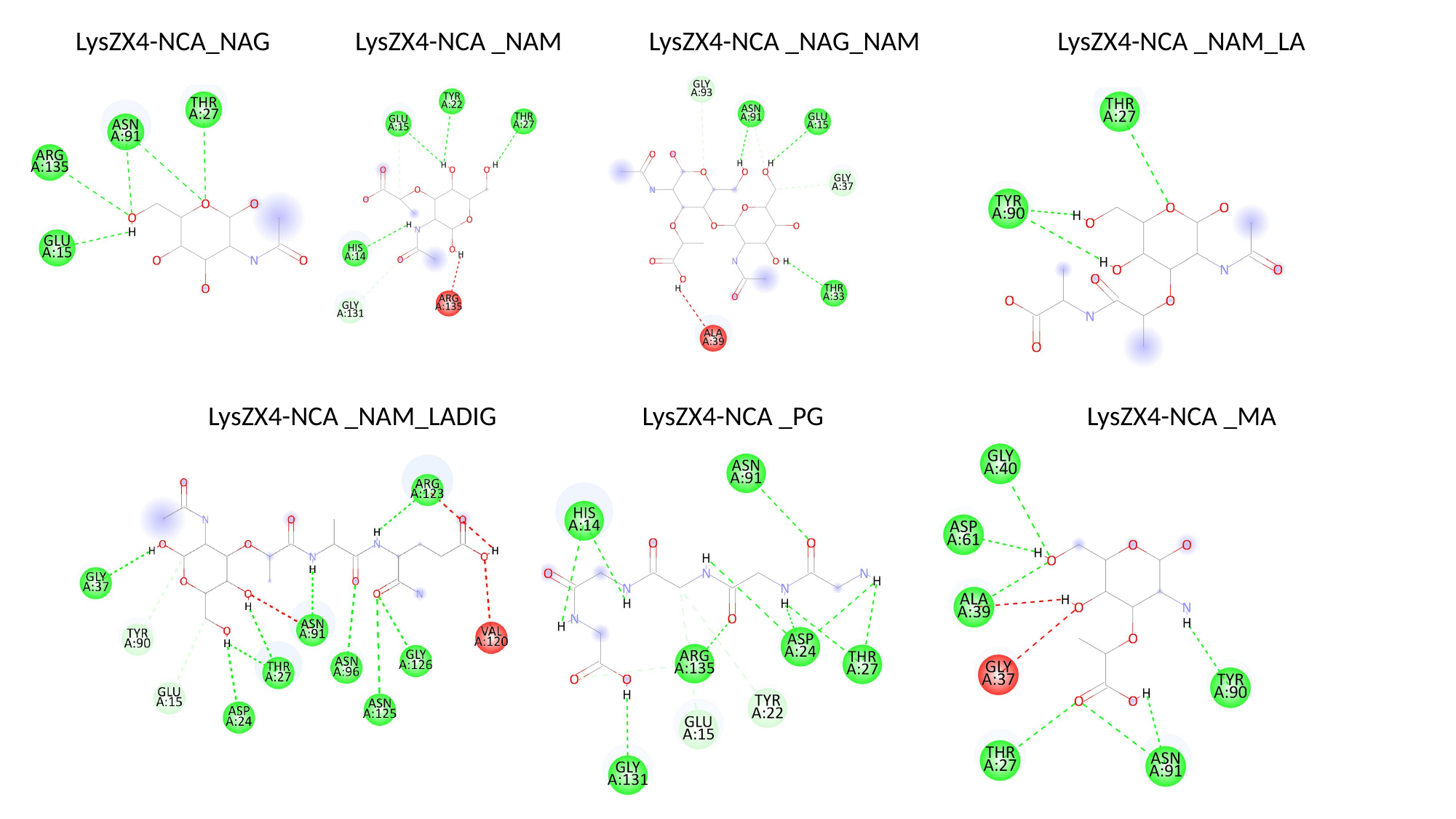

LysZX4-NCA_NAG
LysZX4-NCA _NAM
LysZX4-NCA _NAG_NAM
LysZX4-NCA _NAM_LA
LysZX4-NCA _NAM_LADIG
LysZX4-NCA _PG
LysZX4-NCA _MA

## Slide 28
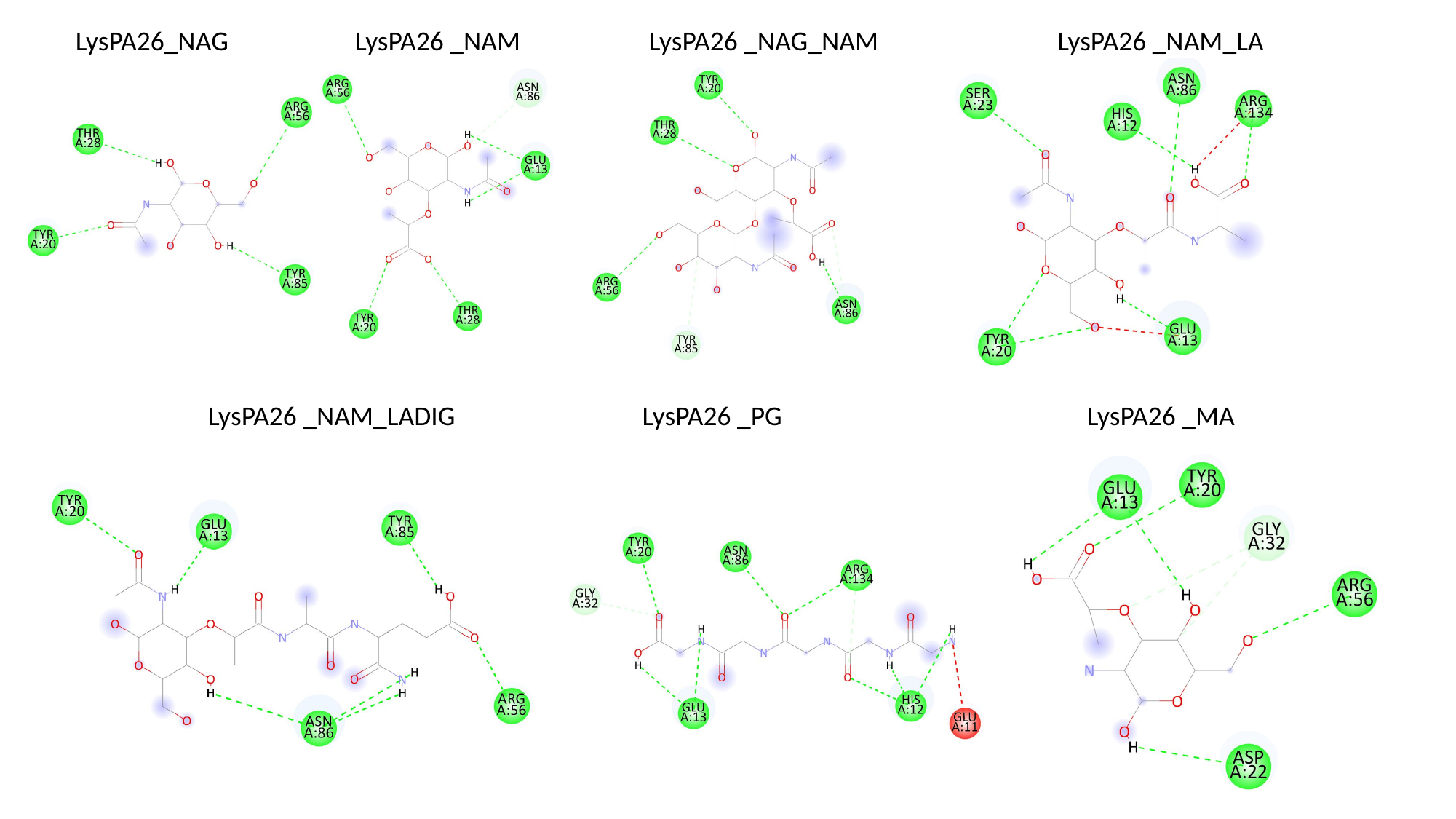

LysPA26_NAG
LysPA26 _NAM
LysPA26 _NAG_NAM
LysPA26 _NAM_LA
LysPA26 _NAM_LADIG
LysPA26 _PG
LysPA26 _MA

## Slide 29
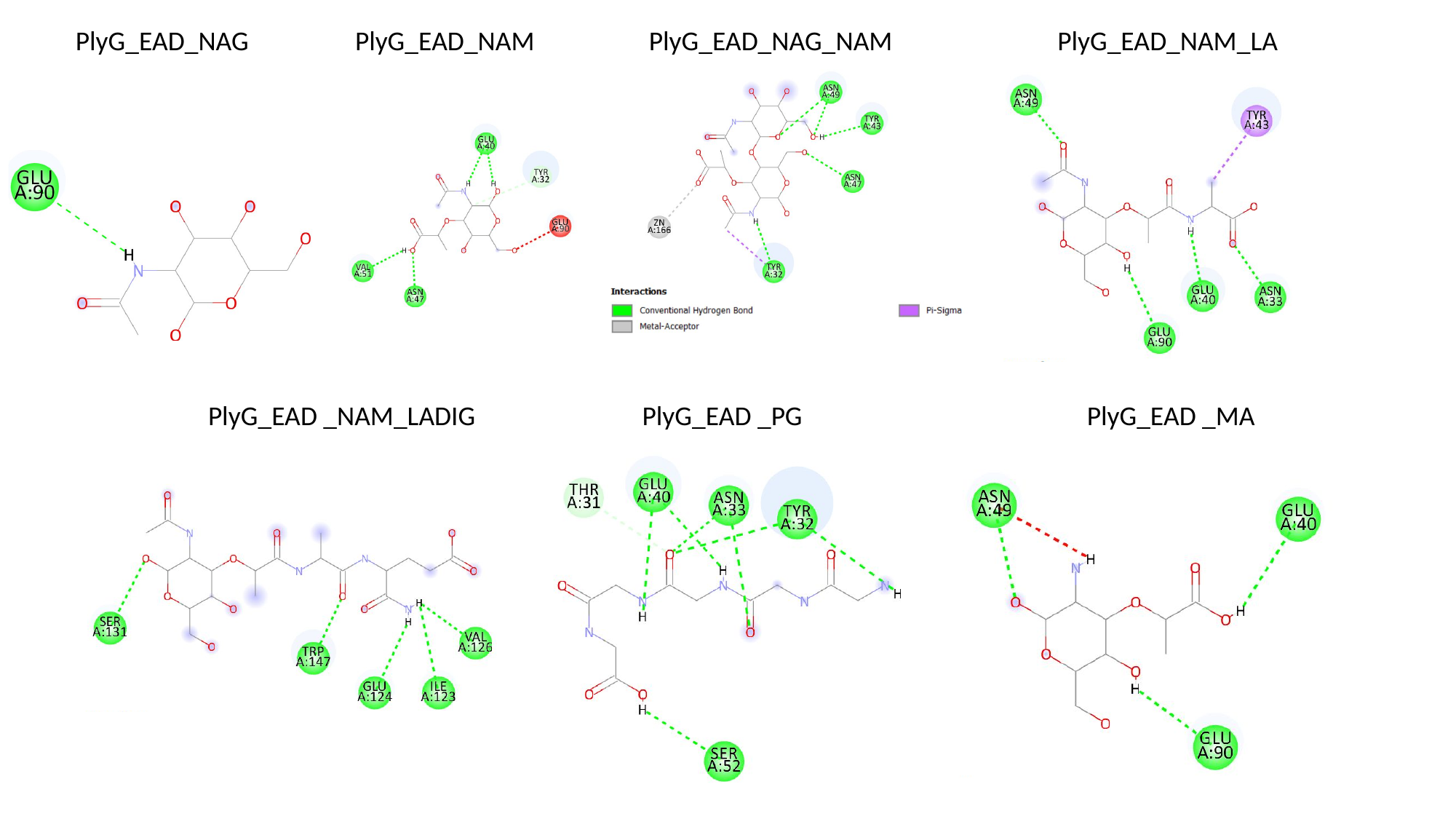

PlyG_EAD_NAG
PlyG_EAD_NAM
PlyG_EAD_NAG_NAM
PlyG_EAD_NAM_LA
PlyG_EAD _NAM_LADIG
PlyG_EAD _PG
PlyG_EAD _MA

## Slide 30
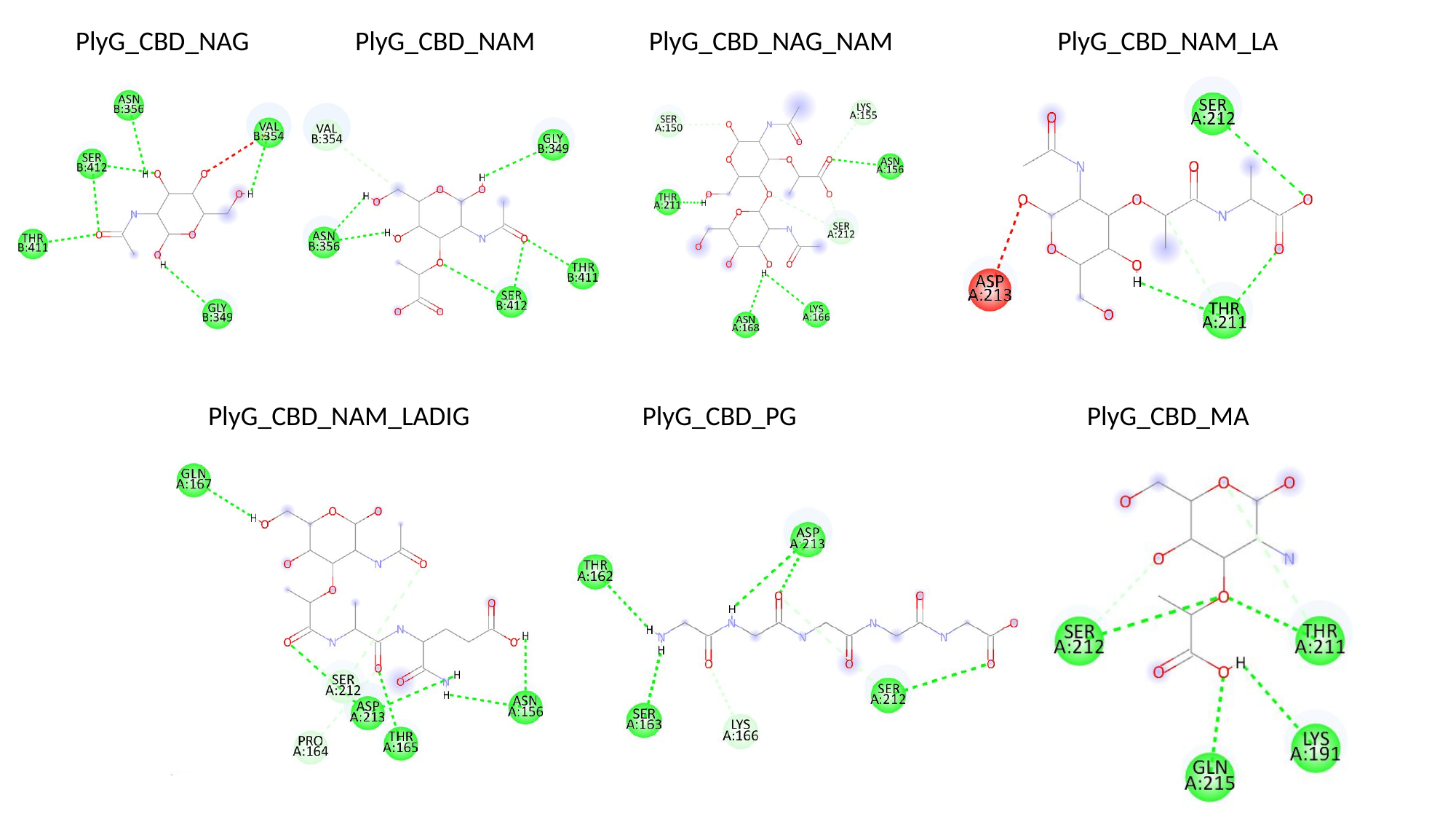

PlyG_CBD_NAG
PlyG_CBD_NAM
PlyG_CBD_NAG_NAM
PlyG_CBD_NAM_LA
PlyG_CBD_NAM_LADIG
PlyG_CBD_PG
PlyG_CBD_MA

## Slide 31
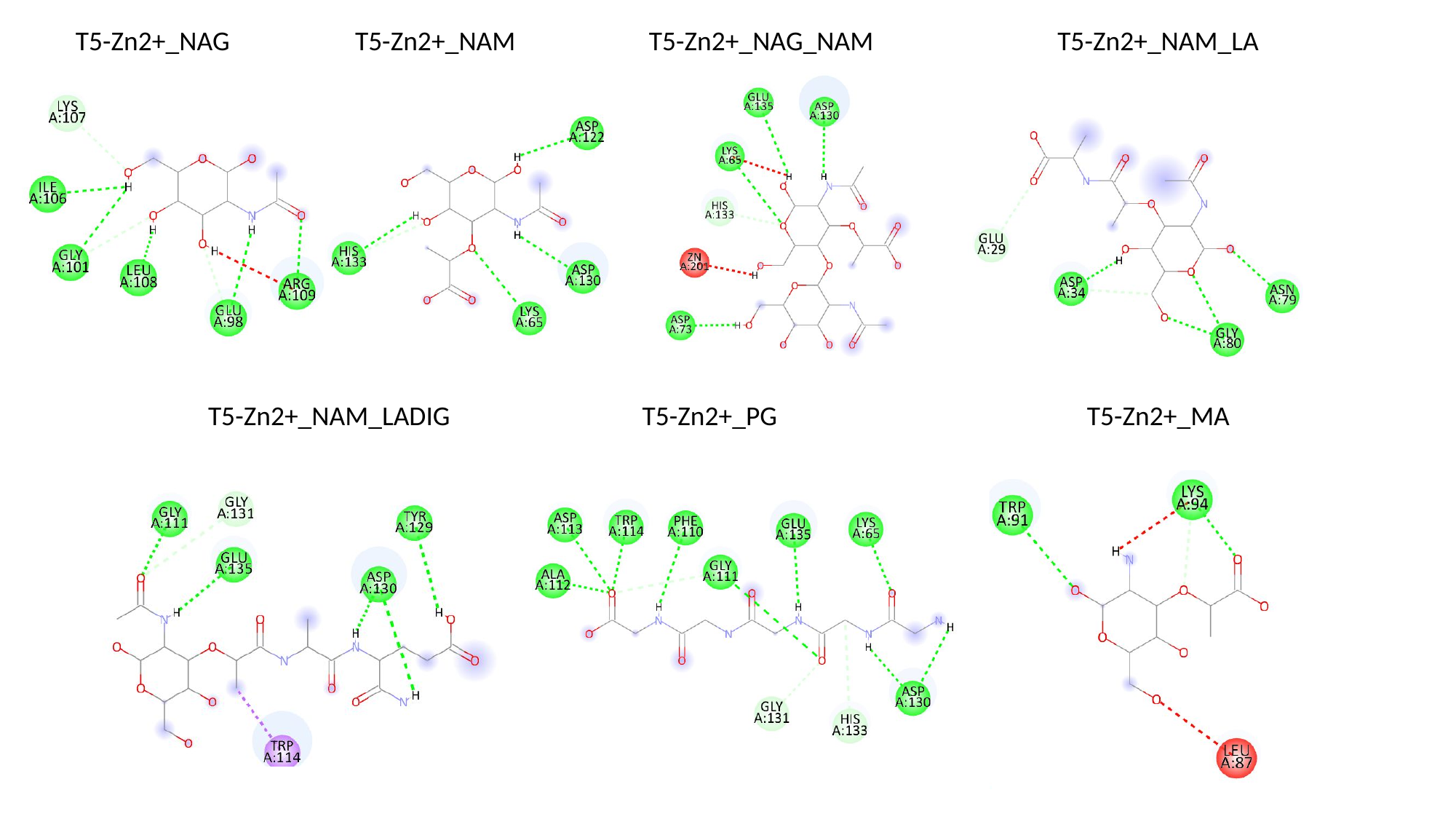

T5-Zn2+_NAG
T5-Zn2+_NAM
T5-Zn2+_NAG_NAM
T5-Zn2+_NAM_LA
T5-Zn2+_NAM_LADIG
T5-Zn2+_PG
T5-Zn2+_MA

## Slide 32
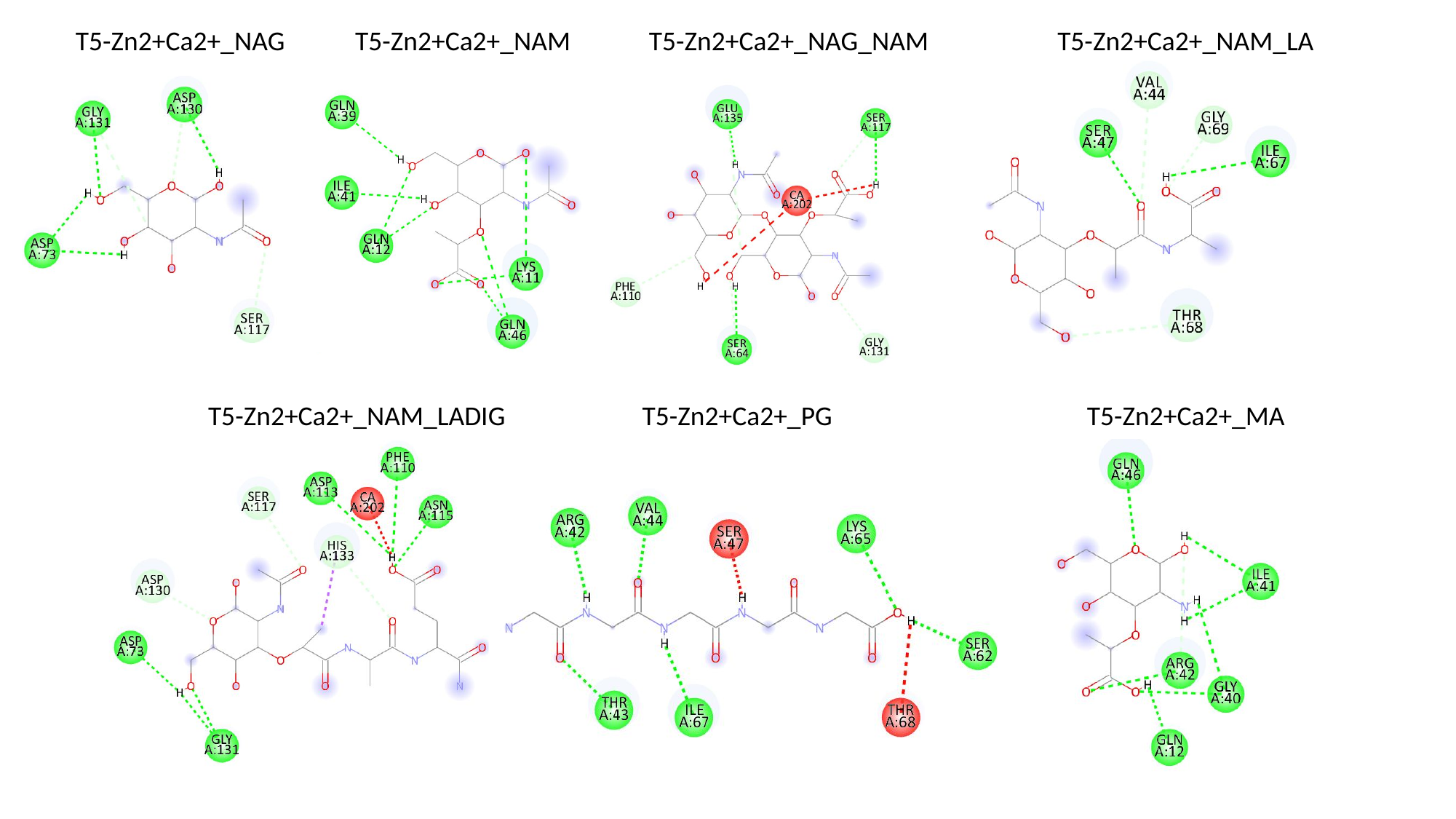

T5-Zn2+Ca2+_NAG
T5-Zn2+Ca2+_NAM
T5-Zn2+Ca2+_NAG_NAM
T5-Zn2+Ca2+_NAM_LA
T5-Zn2+Ca2+_NAM_LADIG
T5-Zn2+Ca2+_PG
T5-Zn2+Ca2+_MA
